# Across established non-invasive neuromodulation and emerging deep-targeting strategies: a comparative bibliometric analysis of TI, TMS, tDCS, and tFUS/TUS/LIFU

**DOI:** 10.3389/fnins.2026.1922512

**Published:** 2026-10-13

**Authors:** Guodong Wang, Shuang Wu

**Affiliations:** 1 Department of Rehabilitation Medicine, Affiliated Hospital of Guizhou Medical University, Guiyang, Guizhou, China; 2 School of Clinical Medicine, Guizhou Medical University, Guiyang, Guizhou, China

**Keywords:** bibliometric analysis, low-intensity focused ultrasound, non-invasive brain stimulation, temporal interference stimulation, transcranial direct current stimulation, transcranial focused ultrasound, transcranial magnetic stimulation

## Abstract

Non-invasive brain stimulation (NIBS) is expanding from conventional cortical modulation toward approaches capable of engaging deeper brain targets, but the literature structures of major modalities have rarely been compared within a common framework. We conducted a comparative bibliometric mapping review of temporal interference stimulation (TI), transcranial magnetic stimulation (TMS), transcranial direct current stimulation (tDCS), and transcranial focused ultrasound and related low-intensity ultrasound stimulation (tFUS/TUS/LIFU), using Web of Science Core Collection and Scopus records indexed from January 2017 to June 2026. After deduplication, relevance screening, targeted re-auditing, and corpus quality control, 32,017 records were retained (162 TI, 20,179 TMS, 10,576 tDCS, and 1,100 tFUS/TUS/LIFU). A strict ultrasound-neuromodulation core set (*n* = 843) was used for modality-specific comparisons, giving a comparative dataset of 31,760 records. TI had the fastest relative growth from 2017 to 2025 (CAGR 64.6%) but the smallest absolute increase. In 10,000 size- and year-matched resamples, TI showed lower author-keyword Shannon diversity and higher Top-10 keyword concentration than matched TMS, tDCS, and tFUS/TUS/LIFU samples after joint Benjamini–Hochberg correction. The thematic pattern was also preserved when comparator corpora were expanded after record-level adjudication, when inclusion was restricted to title-defined records, and when the historical title-term procedure was applied to the contemporary datasets. The six thematic comparisons also remained significant under two-sided empirical testing. In contrast, when each modality was aligned to its own first 9 years, early TMS and tDCS were more thematically concentrated than TI, whereas early tFUS was less concentrated; this pattern persisted after broadening the historical tFUS corpus. Recent TI research activity also includes direct human deep-target validation and patient studies, alongside several registered clinical trials. These developments indicate translational activity beyond modeling and early proof-of-concept work. Together, these findings do not support a simple monotonic relationship between field age and thematic concentration across NIBS modalities, indicating that contemporary thematic concentration should not be interpreted as a direct proxy for bibliometric maturity.

## 1 Introduction

Non-invasive brain stimulation (NIBS) has evolved from a set of tools primarily used to modulate cortical excitability into a broader technological framework spanning cognitive neuroscience, neuropsychiatric treatment, rehabilitation, and deep-targeted neuromodulation. With the development of transcranial magnetic stimulation (TMS), transcranial direct current stimulation (tDCS), transcranial focused ultrasound and related low-intensity ultrasound stimulation (tFUS/TUS/LIFU), and temporal interference stimulation (TI), the field is no longer characterized by the expansion of a single technical pathway. Instead, it now comprises a multilayered landscape in which established clinical tools coexist with emerging strategies for deep brain modulation.

Among the four modalities examined here, TMS and tDCS have the largest and most established literature bases. Since Barker et al. (1985) first demonstrated non-invasive magnetic stimulation of the human motor cortex, TMS has been widely used to induce cortical activation through electromagnetic induction and has accumulated extensive clinical evidence, including evidence-based guidelines and safety recommendations for indications such as depression (Rossi et al., 2009, 2021; Lefaucheur et al., 2020). tDCS modulates neuronal resting membrane potentials through weak direct current stimulation (Nitsche and Paulus, 2000). Owing to its simplicity, low cost, and ease of implementation, tDCS has been widely applied in cognitive research and rehabilitation, with corresponding therapeutic and safety guidelines now available (Antal et al., 2017; Lefaucheur et al., 2017). Together, TMS and tDCS constitute major components of both clinical and basic NIBS research (Polanía et al., 2018).

Despite their broad use in basic and clinical research, conventional NIBS techniques face a fundamental physical trade-off between stimulation depth and focality. Stimulating deeper brain regions often requires broader or stronger superficial field exposure, whereas improving spatial focality may limit effective stimulation of deep targets (Deng et al., 2013). Therefore, achieving relatively focal modulation of deep brain regions while maintaining non-invasiveness has become an important direction in recent NIBS development. This physical constraint also provides the rationale for the emergence of deep-targeting strategies such as tFUS and TI.

At the same time, two emerging classes of techniques are extending the boundaries of deep-targeted neuromodulation. Transcranial focused ultrasound and related low-intensity ultrasound stimulation (tFUS/TUS/LIFU) exploit the favorable penetration and focusing properties of acoustic energy to achieve millimeter-scale targeting of deep brain structures (Legon et al., 2014; Fomenko et al., 2018; Blackmore et al., 2019; Folloni et al., 2019; Darmani et al., 2022). This line of research includes both ultrasound-based neuromodulation aimed at regulating neural activity and translational applications related to brain-targeted delivery and blood–brain barrier opening (Rezai et al., 2020). These non-neuromodulation applications were considered part of the broader focused-ultrasound field but were excluded from the strict tFUS/TUS/LIFU comparative dataset used in the present analyses. Temporal interference stimulation (TI), by contrast, has been proposed as a non-invasive deep-targeting strategy in which overlapping kilohertz electric fields generate an amplitude-modulated interference pattern. Its mechanisms of action, neural sensitivity to kilohertz stimulation, dose–response relationships, and the degree of deep-target selectivity achievable *in vivo* remain active areas of investigation. Since Grossman et al. (2017) introduced the approach, TI research has expanded from computational and animal studies to human studies targeting deep brain structures, including the hippocampus and striatum (Acerbo et al., 2022; Violante et al., 2023; Wessel et al., 2023; Vassiliadis et al., 2024). More recent studies have extended this work into patient populations, including Parkinson's disease and epilepsy, with direct evaluation of deep targets and, in some studies, intracranial or depth-electrode recordings (Lamoš et al., 2025; Yang et al., 2025; Yang et al., 2026; Missey et al., 2026; Xu et al., 2026). These developments indicate that TI remains an emerging modality but is no longer confined to modeling and initial healthy-volunteer proof-of-concept work.

Although these four modalities differ markedly in mechanism, application scope, historical depth, and contemporary literature size, systematic cross-modality comparisons of their publication trajectories, geographic patterns, thematic organization, and knowledge bases remain limited. Here, we use the term bibliometric knowledge structure to describe the accumulation, breadth, thematic organization, collaboration patterns, and reference-based organization of a modality-specific literature. These features are not treated as direct measures of clinical efficacy, safety, regulatory status, or a unidimensional stage of technological maturity. Instead, the four modalities are compared as a focused, non-exhaustive framework for examining a bibliometric knowledge-structure spectrum across established and emerging NIBS technologies.

The four-modality framework was intentionally focused rather than exhaustive. TMS and tDCS were selected as established electromagnetic and direct-current electrical comparators, respectively; tFUS/TUS/LIFU was included as an acoustic deep-targeting comparator; and TI represented the focal emerging technology of interest. tDCS was used as the representative direct-current transcranial electrical stimulation comparator, and tACS was therefore not introduced as an additional fifth modality. Under the prespecified relevance Codebook, records in which tACS rather than the target modality constituted the substantive intervention were classified as other-modality records. Because TI is mechanistically and terminologically related to alternating-current stimulation, however, TI records containing prominent tACS terminology were re-audited during revision to ensure that substantively TI-focused studies were not excluded solely because of their indexed terminology. Accordingly, the present framework should not be interpreted as a census of all NIBS or transcranial electrical stimulation technologies.

The present study had four objectives:

(1) to compare publication volume and relative and absolute growth across TI, TMS, tDCS, and tFUS/TUS/LIFU;
(2) to characterize their geographic participation and international collaboration structures;
(3) to compare thematic diversity, thematic concentration, and reference-based knowledge structures; and
(4) to evaluate the robustness of cross-modality differences using complementary sensitivity analyses addressing corpus definition, sample-size and annual-composition imbalance, outcome measurement, statistical-test specification, and field age.

## 2 Materials and methods

### 2.1 Study design and reporting framework

This study was designed as a comparative bibliometric mapping review. The workflow followed established bibliometric procedures, including multi-database retrieval, cross-database deduplication, relevance screening, performance analysis, science mapping, strict-core sensitivity analysis, matched resampling, and a complementary field-age-aligned historical sensitivity analysis. A PRISMA-style flow diagram was used to transparently document record identification, screening, and inclusion. Because the study mapped bibliographic and knowledge structures rather than synthesizing intervention effects, study-level risk-of-bias assessment was not performed.

### 2.2 Data sources and search strategy

The Web of Science Core Collection (WoS) and Scopus were used as the primary data sources in this study. Publications related to the four NIBS modalities published between January 2017 and June 2026 were retrieved. The year 2017 was selected because TI was introduced as a non-invasive deep-targeting strategy in that year. Applying a common starting year allowed all four modalities to be compared within the same contemporary observation window. However, this design does not capture the earlier historical development and accumulated knowledge bases of TMS, tDCS, and tFUS/TUS/LIFU before 2017. To address this temporal asymmetry directly, we conducted a separate field-age-aligned historical retrieval as a complementary sensitivity analysis (Section 2.10; Supplementary method S10). This historical analysis used modality-specific 9-year windows and was analyzed independently from the main 2017–2026 dataset; it did not replace or modify the primary contemporary bibliometric corpus. The WoS and PubMed searches were completed on June 20, 2026, and the Scopus search was completed on June 21, 2026. For each modality, a modality-specific main search strategy was constructed using standardized technical terms and commonly used abbreviations as core search terms. Additional terms related to the brain, neural systems, cortex, and neuromodulation were used to restrict the search to a neuroscience context. The complete main and sensitivity search strategies, together with the PubMed gap-check strings and corresponding record counts, are provided in Supplementary Table S1. For the Web of Science and Scopus main and sensitivity searches, document types were limited to original research articles and reviews. PubMed was used only as an independent biomedical gap-check source and was not subject to the same document-type restriction as the primary analytic databases.

To evaluate the sensitivity of the main search strategies, expanded searches were additionally conducted using broader abbreviations and variant terms. For TMS, tDCS, and tFUS/TUS/LIFU, these searches did not alter the primary corpora. For an additional robustness analysis, however, sensitivity-only TMS and tFUS/TUS/LIFU candidates were adjudicated at the record level and used to construct expanded comparator corpora for a separate robustness analysis (Section 2.9; Supplementary method S14; Supplementary Table S19). For TI, the expanded search recovered additional records using abbreviations and variant terms such as tTIS and TIS. These records underwent record-level manual adjudication, and substantively TI-relevant records that were not already present in the main corpus were incorporated into the revised TI dataset after DOI- and normalized-title-level deduplication. A separate targeted re-audit was also performed for records containing prominent tACS-related terminology to ensure that substantively TI-focused studies had not been excluded solely because of terminology overlap with alternating-current stimulation. PubMed records were not pooled with the WoS–Scopus analytic corpus.

### 2.3 Data cleaning and integration

WoS records exported in plain-text format and Scopus records exported in CSV format were imported into R using the convert2df function of the bibliometrix package (Aria and Cuccurullo, 2017). Cross-database deduplication was first performed based on DOI and title matching, yielding 33,026 unique records. Relevance screening was then conducted by requiring prespecified modality terms to appear in the title or author keywords (DE field), in order to exclude records primarily dominated by other modalities or records that did not substantively address the target modality. Title or author-keyword occurrence was selected as the primary relevance criterion to ensure that the target modality constituted a substantive focus of the record rather than an incidental mention in the abstract. This approach prioritized specificity and cross-modality comparability. Potential sensitivity loss resulting from this criterion was evaluated through expanded searches and independent PubMed gap-checking.

In the pre-revision common relevance screening, 606 records were excluded, accounting for 1.8% of the deduplicated dataset; 564 of these records (93%) were classified as cross-modality contamination dominated by another technology (Supplementary Table S3). After this initial screening, the pre-revision contemporary corpus comprised 32,420 records: 152 TI, 20,297 TMS, 10,871 tDCS, and 1,100 tFUS/TUS/LIFU. During revision, targeted corpus quality-control procedures were applied to the modality-specific datasets. For TI, additional relevant records identified through the expanded search and targeted tACS-related re-audit were incorporated after record-level adjudication and deduplication, yielding a final TI corpus of 162 records. For TMS and tDCS, targeted false-positive terminology and duplicate/republication audits removed ineligible terminology matches and duplicate or republication records, resulting in final corpora of 20,179 TMS and 10,576 tDCS records. The broad tFUS/TUS/LIFU corpus remained unchanged at 1,100 records. The resulting broad-scope contemporary dataset therefore comprised 32,017 records. After application of the strict ultrasound-neuromodulation definition, the comparative dataset contained 31,760 records, including 843 strict-core tFUS/TUS/LIFU records.

To evaluate the potential dependence introduced by using author keywords as part of the primary inclusion rule and as a thematic outcome, a title-only inclusion sensitivity analysis was performed. The same modality-specific title-matching logic used in the main screening workflow was applied to the final contemporary corpora, but author-keyword occurrence was not permitted to satisfy the inclusion criterion. This yielded title-only subsets of 121 TI, 7,835 TMS, 5,050 tDCS, and 473 strict-core tFUS/TUS/LIFU records. The size- and year-matched resampling analysis of author-keyword Shannon diversity and Top-10 keyword concentration was then repeated using these title-only subsets.

### 2.4 Manual relevance audit of modality-specific datasets

To evaluate residual scope heterogeneity in the TI, TMS, and tDCS datasets after the common title/author-keyword relevance screening, we conducted a publication-year-stratified manual relevance audit of 500 records. The audit sample comprised 100 TI records, 200 TMS records, and 200 tDCS records. Within each modality, the sample was allocated proportionally across publication-year strata and records were sampled without replacement. The tFUS/TUS/LIFU dataset was not resampled because all 1,100 ultrasound records had already undergone complete strict classification using prespecified rule-based criteria, with manual verification of borderline and ambiguous records, yielding a strict ultrasound-neuromodulation core set of 843 records. This audit was conducted on the pre-revision corpora generated after the common title/author-keyword screening and was retained as a diagnostic assessment of residual scope heterogeneity at that screening stage.

Records were classified as modality-core or non-core using a prespecified Codebook v2.0. A record was considered modality-core when the target modality constituted a primary or secondary intervention, an independent comparator, a substantive component of a multimodal intervention, a measurement or analytical method generating study variables, or an explicit object of mechanistic, methodological, safety, modeling, guideline, protocol, or evidence-synthesis research. Records were classified as non-core when the target modality was mentioned only incidentally, represented a different stimulation modality, reflected a terminology or abbreviation mismatch, or fell outside the predefined neuromodulation scope. Records initially classified as non-core or considered potentially ambiguous were further verified using the abstract, full text, or publisher record.

For each modality, publication-year-specific core proportions were combined using the corresponding population stratum weights. Design-weighted core proportions were calculated as ${\hat{p}}_{w}=\underset{h}{\sum }W_{h}{\hat{p}}_{h}$, where $W_{h}=\frac{N_{h}}{N}$ represented the population weight of publication-year stratum *h*, and ${\hat{p}}_{h}=\frac{x_{h}}{n_{h}}$ represented the observed core proportion within that stratum. Variance estimates incorporated the finite population correction for sampling without replacement. For strata containing only one audited record, the variance contribution was set to zero according to the prespecified analysis rule; a conservative sensitivity analysis using the maximum binary variance produced only a negligible change in the TI confidence interval. Design-weighted proportions and their 95% confidence intervals were treated as the primary audit estimates, whereas unweighted sample proportions and Wilson 95% confidence intervals were reported descriptively. Because the modality-specific audit samples had different sampling fractions, no pooled design-weighted core proportion was calculated across the three modalities.

To evaluate inter-reviewer reproducibility, a stratified 90-record subset comprising 30 TI, 30 TMS, and 30 tDCS records was independently classified by a second reviewer using the final Codebook v2.0. Overall agreement and Cohen's κ were calculated from the reviewers' original independent classifications before consensus discussion. Disagreements were subsequently resolved through discussion after review of the available abstracts, full texts, or publisher records, but the consensus classifications were not used to recalculate κ. Detailed sampling strata, classification results, exclusion reasons, and agreement statistics are provided in Supplementary Table S13.

### 2.5 Strict ultrasound-neuromodulation screening

Because the broad-scope search for tFUS/TUS/LIFU captured a substantial number of non-neuromodulation records—including blood–brain barrier opening, drug delivery, tumor treatment, high-intensity focused ultrasound (HIFU) ablation, and imaging or engineering studies not directly related to neuromodulation—an additional strict neuromodulation screening was applied to this modality. The 1,100 tFUS/TUS/LIFU records were first classified using an inclusion/exclusion term dictionary (rule-based classification), after which all borderline records and ambiguous core records were manually verified. All rule-based classification, relevance screening, and manual verification of borderline or ambiguous ultrasound records were performed by GW using prespecified operational criteria. The classification definitions and record counts are reported in Supplementary Table S9. This yielded a strict core set of 843 records, 39 core-adjacent records (excluded from the strict comparative analyses but retained in the broad-scope corpus), and 218 non-core records (843 + 39 + 218 = 1,100). This additional strict screening was applied specifically to the ultrasound modality because its terminology overlaps extensively with non-neuromodulation applications such as blood–brain barrier opening, drug delivery, thermal ablation, tumor treatment, and imaging. The other three modalities were retained after the common title/author-keyword relevance screening because they did not show an equivalent systematic overlap with a distinct non-neuromodulation application field. We acknowledge that this modality-specific procedure resulted in a more intensive record-level screening process for tFUS/TUS/LIFU than for the other three modalities. To assess whether this asymmetry influenced the field-age comparison, we therefore conducted an additional historical tFUS sensitivity analysis using a broader corpus that included both strict-core and core-adjacent ultrasound-neuromodulation records. The resulting thematic pattern was compared with that obtained from the strict historical tFUS corpus. Except for record identification and overall dataset-size reporting, all tFUS/TUS/LIFU-specific comparative analyses within the primary 2017–2026 dataset were defined from the 843-record strict-core set. For reference co-citation analysis, the Scopus-linked subset of this strict-core dataset was used, as detailed in Section 2.7. This resulted in two dataset definitions used for the primary contemporary analyses: a broad-scope analytic dataset (*n* = 32,017) and a strict comparative dataset using strict-core tFUS/TUS/LIFU (*n* = 31,760).

### 2.6 Bibliometric and visualization analyses

Bibliometric analyses and visualizations were performed using R, the bibliometrix package, and VOSviewer (van Eck and Waltman, 2010). Annual publication trends and normalized growth were calculated using 2017 as the baseline year. The compound annual growth rate (CAGR) was calculated over complete publication years from 2017 to 2025, whereas 2026 was treated as an incomplete year and reported separately. Absolute growth was calculated as the difference between annual publication output in 2025 and that in 2017. This measure was analyzed alongside CAGR to distinguish absolute field expansion from relative growth affected by the initial publication baseline. In the geographic analyses, national publication output was counted according to the corresponding author's country. The number of contributing countries and the multiple-country publication ratio (MCP) were calculated from all authors' country information. MCP was defined as the proportion of publications involving authors from two or more countries among records with available country information; records lacking country information were excluded from the MCP denominator. International collaboration and author-keyword co-occurrence networks were constructed in R and exported for visualization in VOSviewer. Reference co-citation networks were exported as VOSviewer map and network files, with node weights and Louvain community assignments supplied from the R-based analysis. The primary contemporary bibliometric analyses were performed in R version 4.5.2 (R Foundation for Statistical Computing, Vienna, Austria) using the bibliometrix package version 5.2.1, with RStudio 2026.05.1 Build 225 as the integrated development environment. Network visualization was performed using VOSviewer version 1.6.20 (Centre for Science and Technology Studies, Leiden University, Leiden, The Netherlands).

Before keyword analysis, author keywords were standardized for case, abbreviations, and synonymous expressions. Broad document-type terms and highly generic methodological labels were removed. For author keyword co-occurrence networks, highly redundant modality self-referential terms and overly broad terms were primarily removed to better highlight research themes related to disease applications, mechanisms, targets, and technical parameters. For annual keyword heatmaps, selected modality-related terms were retained to capture the co-evolution of terminology use and research themes over time. The keyword standardization and exclusion rules are summarized in Supplementary Table S8.

For the highly cited source-document analysis, representative documents were identified from the Web of Science-indexed subset of each modality-specific dataset and ranked exclusively using the Web of Science Core Collection TC field. Web of Science and Scopus citation counts were not combined or summed. For each modality, only documents classified as modality-core were retained in the main ranking; core-adjacent highly cited documents were reported separately in Supplementary Table S7.

### 2.7 Data basis for reference co-citation analysis

Reference co-citation analysis was conducted exclusively using Scopus cited-reference data. For TI, TMS, and tDCS, the corresponding modality-specific Scopus main datasets were used. For tFUS/TUS/LIFU, the 843-record strict ultrasound-neuromodulation core set was linked back to the broad-scope Scopus dataset using hierarchical record matching. EID/UT was used first, followed by DOI matching; a one-to-one normalized title-and-publication-year fallback was applied only to unmatched strict-core records without a DOI. This procedure uniquely matched 784 strict-core records to Scopus, of which 779 (99.4%) contained structured cited-reference data. Web of Science and Scopus cited-reference fields were not combined because differences in reference-string formatting could split the same cited document into multiple network nodes.

Before network construction, cited-reference strings were canonicalized to reduce fragmentation caused by author-list truncation, punctuation differences, electronic-page formatting, journal-title abbreviations, and other bibliographic variations. Each cited reference was assigned a canonical key based on the normalized first-author surname, publication year, and standardized cited title. Reference strings sharing the same canonical key were merged into a single node, and the most complete bibliographic string was retained as the display label. References for which title information could not be extracted reliably were retained using the normalized complete reference string rather than being automatically merged. This conservative procedure reduced reference fragmentation while limiting the risk of false-positive consolidation.

A binary source-document-by-cited-reference matrix was constructed for each modality. The cross-product of this matrix yielded the cited-reference co-citation matrix. Its diagonal represented cited-reference occurrence, defined as the number of source documents citing a given reference, whereas off-diagonal values represented weighted pairwise co-citation frequencies. Nodes were retained using modality-specific lower, main, and higher minimum cited-reference occurrence thresholds. The lower, main, and higher minimum cited-reference occurrence thresholds were 3, 5, and 8 for TI; 80, 120, and 200 for TMS; 80, 120, and 180 for tDCS; and 11, 22, and 44 for strict-core tFUS/TUS/LIFU. These modality-specific thresholds were used as operational cutoffs to obtain interpretable reference networks despite the marked differences in corpus size. Cross-modality interpretation was not based on absolute community counts or modularity values, and robustness was evaluated using the lower and higher threshold settings. Isolated nodes were removed after thresholding. Each Figure 1 panel displays the complete main-threshold network rather than a fixed-size Top-N subset.

**Figure 1:**
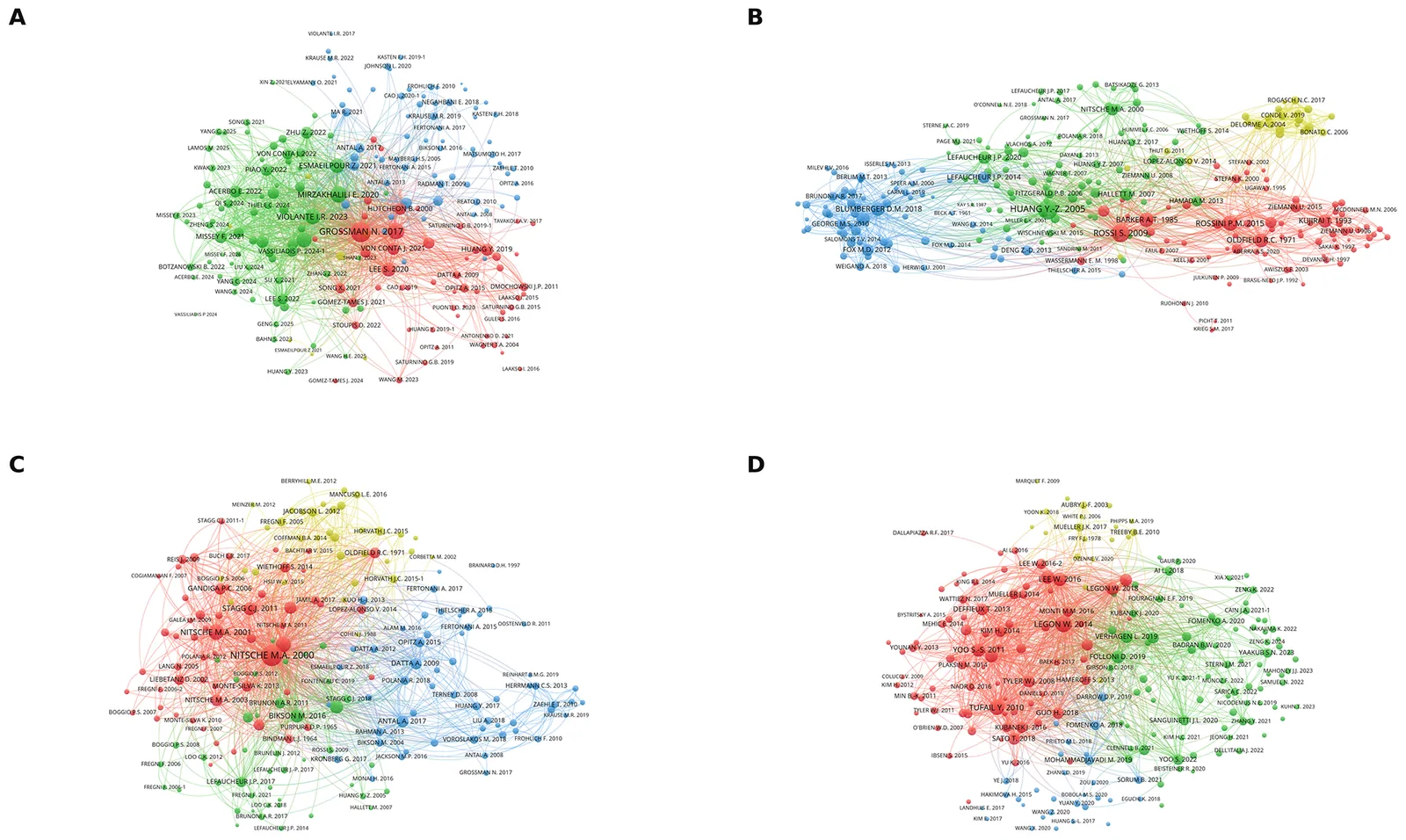
Reference co-citation networks for **(A)** TI, **(B)** TMS, **(C)** tDCS, and **(D)** strict-core tFUS/TUS/LIFU. Networks were constructed exclusively from Scopus cited-reference data after cited-reference canonicalization. Each panel displays the complete main-threshold network after removal of isolated nodes. The minimum cited-reference occurrence thresholds were 5 for TI, 120 for TMS, 120 for tDCS, and 22 for strict-core tFUS/TUS/LIFU, yielding 206, 253, 192, and 212 nodes and 12,078, 26,448, 17,578, and 20,273 weighted co-citation edges, respectively. Nodes represent cited references, node size indicates cited-reference occurrence, and edge weight indicates weighted pairwise co-citation frequency. Node colors represent the selected Louvain communities. All four main-threshold networks contained four communities. Because modularity values were modest (0.0974–0.2450), the detected communities were interpreted as coarse descriptive partitions rather than sharply separated intellectual modules. Lower- and higher-threshold results are reported in Supplementary Table S5.

To reduce dependence on vertex ordering, Louvain community detection was evaluated across 100 random vertex-permutation restarts for each thresholded network. For each restart, vertex order was randomly permuted before community detection, and the resulting community memberships were mapped back to the original graph. Mapping accuracy was verified by confirming that modularity was identical before and after reverse mapping. The partition with the highest modularity was retained. When multiple partitions shared the same maximum modularity, the solution corresponding to the most frequent community count among the tied maximum-modularity partitions was selected. Total co-citation link strength was calculated as the sum of weighted co-citation links incident on each node. Network layouts were rendered in VOSviewer, while community colors were derived from the selected igraph Louvain partitions.

### 2.8 Size- and year-matched resampling

To assess whether the observed cross-modality differences in thematic and geographic structure were driven by unequal sample sizes and differing annual compositions, we applied a size- and year-matched resampling procedure. For each comparison modality—TMS, tDCS, and the strict-core tFUS/TUS/LIFU dataset−162 records were sampled without replacement within publication-year strata to reproduce the year-by-year distribution of the 162 TI records. The procedure was repeated independently 10,000 times using a fixed random seed [set.seed(20260724)]. The analysis was repeated using the final revised contemporary corpora after completion of all targeted corpus quality-control procedures.

Six indicators were computed for each resample: the number of unique contributing countries, the multiple-country publication ratio, the mean number of authors per publication, the mean number of author keywords per publication, the Shannon diversity of standardized author keywords, and the concentration of the ten most frequent standardized author keywords. Keyword Shannon diversity was calculated from the relative frequency distribution of standardized author-keyword occurrences. Top-10 keyword concentration was defined as the proportion of all standardized author-keyword occurrences accounted for by the ten most frequent keywords. Accordingly, “thematic diversity” and “thematic concentration” are operationalized here as the diversity and concentration of standardized bibliographic vocabularies and should be interpreted as proxies for thematic structure rather than direct measurements of latent research topics. Author-keyword field availability and the rule-based standardization procedures used across the full and matched datasets are summarized in Supplementary Table S11.

The observed TI value for each indicator was compared with the median and 2.5th−97.5th percentile empirical interval of the 10,000 resamples. One-sided directional tests were used for the prespecified hypotheses that TI would show lower keyword diversity, higher Top-10 keyword concentration, and fewer contributing countries. Two-sided tests were used for the MCP ratio, authors per publication, and author keywords per publication.

Empirical *P* values were calculated using the add-one correction. For one-sided tests, P=(b+1)/(B+1), where b was the number of resampled values at least as extreme as the observed TI value in the prespecified direction and *B* = 10,000. For two-sided tests, the corrected probability in the smaller empirical tail was doubled and capped at 1. The 18 primary *P* values arising from six indicators across three comparison modalities were adjusted jointly using the Benjamini–Hochberg false discovery rate procedure. This procedure controls differences in corpus size and annual publication composition within the common 2017–2026 observation window but does not remove differences in pre-2017 field age. Field age was therefore examined separately in the historical sensitivity analysis described in Section 2.10.

### 2.9 Other sensitivity and robustness analyses

In addition to strict ultrasound-neuromodulation screening and the primary size- and year-matched resampling analysis, several complementary robustness analyses were conducted. First, expanded searches and targeted record-level adjudication were used to assess potential retrieval omissions. For TI, substantively relevant records identified through expanded-search adjudication and targeted re-audit were incorporated into the revised corpus after deduplication. For TMS and tFUS/TUS/LIFU, adjudicated sensitivity-only records were used to construct expanded comparator corpora for a separate matched-resampling analysis; tDCS remained unchanged.

Second, the thematic comparisons were repeated under alternative inclusion and measurement specifications: title-only inclusion, modality-family keyword harmonization, and a title-term representation using the same preprocessing framework as the historical analysis. These analyses assessed potential dependence on author-keyword-based inclusion, modality terminology, and thematic measurement instrument.

Third, the field-age analysis was repeated using a broader historical tFUS corpus including core-adjacent records, and the primary and expanded-comparator thematic comparisons were recalculated using two-sided empirical *P* values. Detailed procedures and results are provided in Supplementary methods S11–S15 and Supplementary Tables S12, S16–S20.

### 2.10 Field-age-aligned sensitivity analysis

To examine whether cross-modality differences in thematic structure could be explained by unequal field age, we conducted a complementary field-age-aligned sensitivity analysis using nine consecutive calendar-year windows anchored to a field-defining modern introduction year for each modality: TMS, 1985–1993; tDCS, 2000–2008; tFUS, 2010–2018; and TI, 2017–2025. A 9-year window was selected because TI contributed nine complete publication years from its introduction in 2017 through 2025; the same window length was then applied to each modality, while the incomplete 2026 publication year was excluded. The historical Web of Science and Scopus searches were conducted on August 17, 2026, with document types restricted to Articles and Reviews. The field-age windows were anchored to field-defining modern introductions of TMS, tDCS, tFUS, and TI (Barker et al., 1985; Nitsche and Paulus, 2000; Tufail et al., 2010; Grossman et al., 2017), corresponding to 1985–1993, 2000–2008, 2010–2018, and 2017–2025, respectively. These anchors defined the field-age windows rather than the first record meeting the analytic document-type criteria. Accordingly, the Barker et al. report, classified as a Letter, was used as the TMS temporal anchor but was not included in the Article/Review analytic corpus. Historically expanded modality terminology was used for retrieval, followed by complete record-level relevance assessment under Codebook v2.0. Complete historical search strings and record-flow counts are provided in Supplementary Table S15. The final field-age-aligned corpora comprised 439 TMS, 158 tDCS, 76 strict-core tFUS, and 99 TI records. For brevity, the strict-core historical ultrasound-neuromodulation corpus is denoted as tFUS in the field-age analysis, while retaining the same tFUS/TUS/LIFU scope definition used elsewhere in the study.

Thematic structure was quantified from standardized English title terms because title coverage was complete across the historical corpora, whereas author-keyword availability varied substantially by modality and publication era. Trailing translated or bilingual title duplicates were removed before tokenization. A prespecified cross-modality stopword dictionary removed standard English stopwords, modality self-identifiers, and non-thematic reporting and design terms. Inflectional variants were normalized using English Snowball stemming, with two additional high-confidence concept merges (cortical → cortex and neuroplasticity → plasticity). Each standardized term was counted at most once per article (document–term incidence). Shannon diversity was calculated as H = –Σp_i_ln(p_i_), where p_i_ represents the proportion of standardized document–term incidences contributed by term i. Top-10 concentration (C10) was defined as the summed document frequency of the 10 most frequent standardized terms divided by all standardized document–term incidences. To reduce sample-size dependence, 10,000 simple random subsamples without replacement were drawn at *n* = 76, corresponding to the smallest field-age corpus. A fixed random seed was used for reproducibility [set.seed(20260818)]. TI, TMS, and tDCS were resampled to *n* = 76, whereas the complete tFUS corpus already contained 76 records and therefore served as the fixed reference. Medians and 2.5th−97.5th percentile resampling ranges were reported as descriptive sensitivity estimates rather than formal confidence intervals or hypothesis-test *P* values. To allow direct comparison of the thematic measurement instrument across timeframes, the same prespecified title-term preprocessing, synonym mapping, and document-term incidence rules were also applied to the final contemporary corpora in a separate sensitivity analysis described in Section 2.9. The field-age historical workflow was performed in R version 4.5.2 using bibliometrix 5.4.1 and the text-processing and resampling packages detailed in Supplementary method S8.

## 3 Results

### 3.1 Dataset construction and relevance screening

Using the main search strategies in the Web of Science Core Collection and Scopus, a total of 53,009 records were imported for cross-database integration, including 23,219 records from WoS and 29,790 records from Scopus. After DOI- and title-based deduplication, 33,026 unique records remained. The initial common relevance screening yielded a pre-revision contemporary corpus of 32,420 records, comprising 152 TI, 20,297 TMS, 10,871 tDCS, and 1,100 tFUS/TUS/LIFU records.

Targeted corpus quality control performed during revision resulted in modality-specific changes. For TI, record-level adjudication of expanded-search candidates and a targeted re-audit of records with prominent tACS-related terminology identified additional substantively TI-relevant records that were not duplicates of the existing corpus. Of the 12 records excluded during the original TI relevance screening, six contained prominent tACS-related terminology and were specifically re-audited; five were judged substantively TI-relevant and restored to the corpus, whereas one remained excluded. In addition, five TI-relevant records identified through expanded-search adjudication were newly incorporated after deduplication; these records were distinct from the five records restored through the tACS-related re-audit. The final TI corpus therefore increased from 152 to 162 records. Targeted false-positive terminology and duplicate/republication audits reduced the TMS corpus to 20,179 records and the tDCS corpus to 10,576 records. The broad tFUS/TUS/LIFU corpus remained unchanged at 1,100 records. The final broad-scope contemporary dataset consequently comprised 32,017 records.

For tFUS/TUS/LIFU, strict neuromodulation screening retained 843 records as strict core, while 39 core-adjacent and 218 non-core records were excluded from the primary modality-specific comparisons. The final strict comparative dataset therefore comprised 31,760 records (162 TI, 20,179 TMS, 10,576 tDCS, and 843 strict-core tFUS/TUS/LIFU). PubMed remained an independent gap-check source and was not pooled with the WoS–Scopus analytic corpus (Figure 2; Supplementary Tables S1–S3).

**Figure 2:**
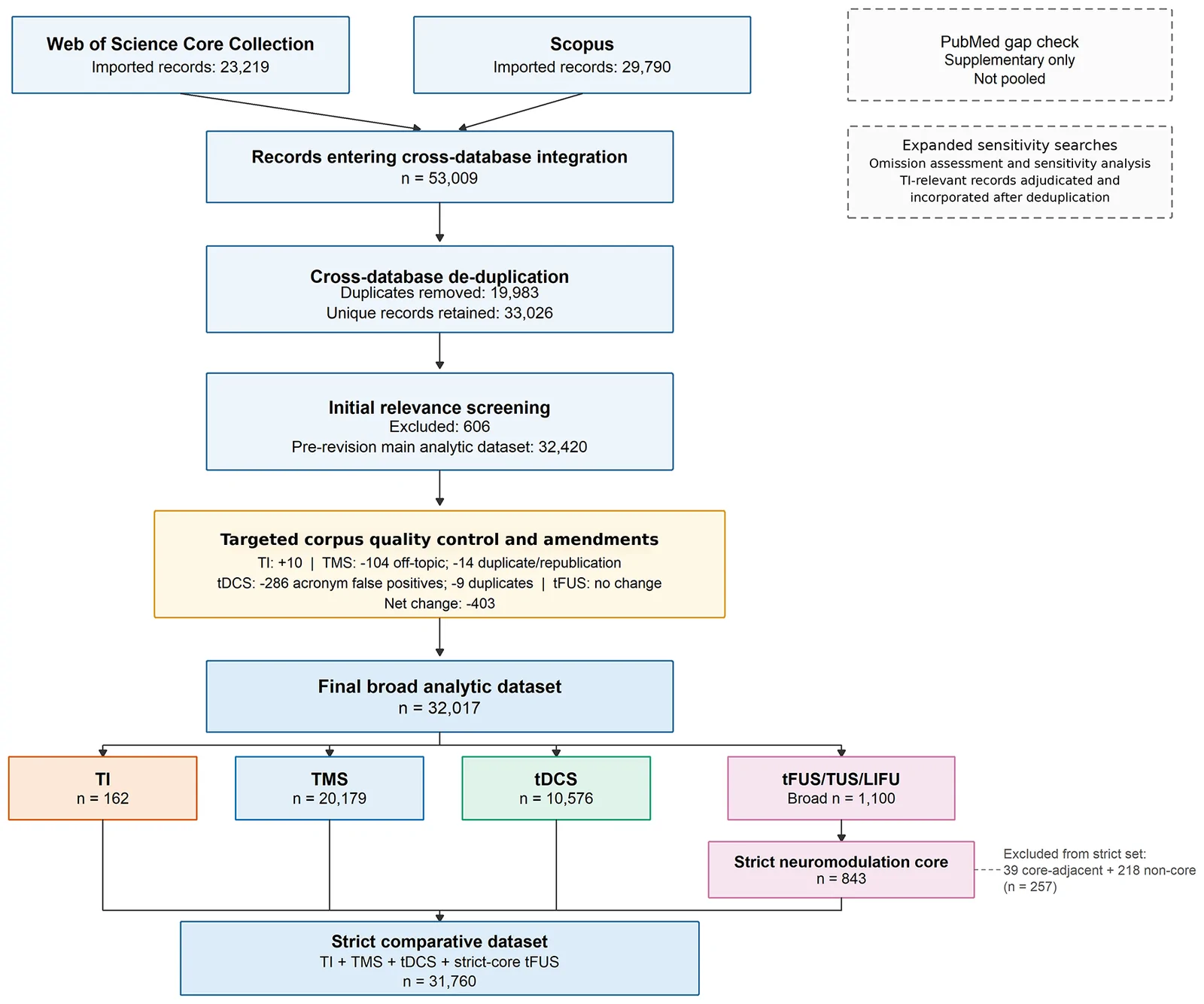
Flow diagram of literature retrieval, cross-database deduplication, relevance screening, targeted corpus quality control, and construction of the final comparative dataset. Web of Science Core Collection and Scopus records were merged and deduplicated by DOI and normalized title. PubMed was used as an independent gap-check source and was not pooled into the analytic corpus. Expanded sensitivity searches were used for omission assessment and sensitivity analyses. For TI, substantively relevant sensitivity-only records were incorporated after record-level adjudication and deduplication. For TMS and tFUS/TUS/LIFU, sensitivity-only records identified through the expanded searches were adjudicated for the expanded-comparator robustness analysis but did not alter the primary corpus and are therefore not shown as additions in this flow diagram. The final broad-scope contemporary dataset contained 32,017 records: 162 TI, 20,179 TMS, 10,576 tDCS, and 1,100 tFUS/TUS/LIFU. The ultrasound subset underwent strict neuromodulation screening, yielding 843 strict-core, 39 core-adjacent, and 218 non-core records. The final strict comparative dataset therefore contained 31,760 records.

### 3.2 Annual publication trends reveal contrasting field sizes and growth trajectories

Across the complete publication years from 2017 to 2025, the four modalities showed marked differences in publication volume and growth. TMS had the largest annual output, increasing from 1,547 records in 2017 to 2,740 in 2025. tDCS increased from 721 to 1,443 records over the same period. TI and strict-core tFUS/TUS/LIFU remained substantially smaller in absolute volume but showed faster relative growth: TI increased from 1 record in 2017 to 54 in 2025, whereas tFUS/TUS/LIFU increased from 16 to 176 records (Figure 3; Table 1).

**Figure 3:**
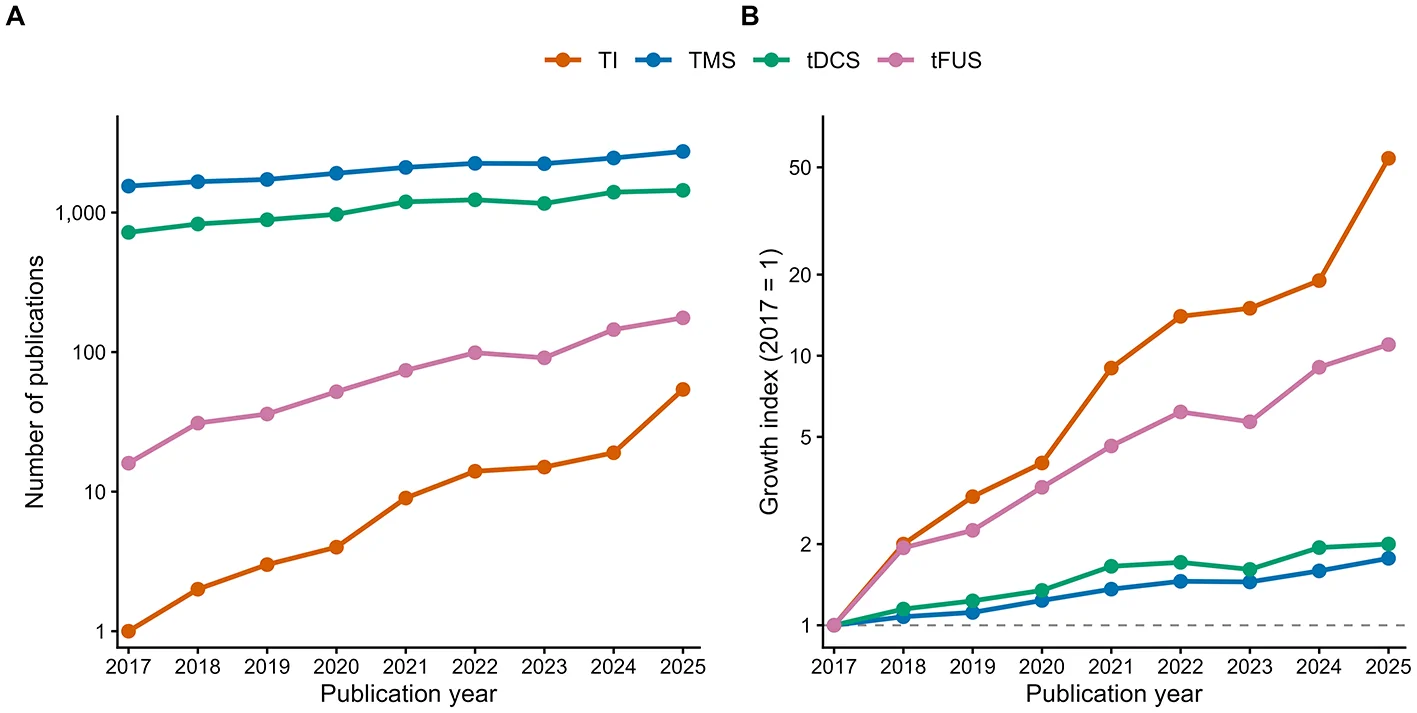
Annual publication trends from 2017 to 2025. **(A)** Absolute number of publications per year for each modality. **(B)** Growth trends normalized to 2017 as the baseline year. The tFUS/TUS/LIFU series was calculated using the strict neuromodulation core set (*n* = 843). The y-axis in both panels is logarithmic. The 2026 partial year was excluded from growth calculations.

**Table 1:** Overview of the four neuromodulation datasets from January 2017 to June 2026.

Modality	Records	2017	2025	CAGR (2017–2025)	Contributing countries	International collaboration (MCP)	Absolute increase, 2017–2025
TI	162	1	54	64.6%	29	25.6%	53
TMS	20,179	1,547	2,740	7.4%	112	24.0%	1,193
tDCS	10,576	721	1,443	9.1%	102	20.3%	722
tFUS/TUS/LIFU	843	16	176	35.0%	41	21.0%	160

Summary of core bibliometric indicators for each modality. CAGR indicates the compound annual growth rate calculated over complete publication years from 2017 to 2025. MCP indicates the multiple-country publication ratio among records with available country information and was calculated from all-author affiliation countries. Contributing-country counts were likewise derived from all-author affiliations. Because the baseline publication count for TI in 2017 was one record, its CAGR should be interpreted with caution. For tFUS/TUS/LIFU, all values are based on the strict neuromodulation core set (n = 843).

Over the complete 2017–2025 interval, TI had the highest compound annual growth rate (CAGR, 64.6%), followed by tFUS/TUS/LIFU (35.0%), tDCS (9.1%), and TMS (7.4%). Because the TI baseline in 2017 consisted of only one record, its normalized growth and CAGR remain sensitive to the small-base effect. The publication trajectories therefore indicate rapid proportional expansion of TI and tFUS/TUS/LIFU from comparatively small literature bases, whereas TMS and tDCS continued to expand from much larger established corpora.

### 3.3 Absolute and relative growth reveal contrasting trajectories

Absolute and relative growth showed different cross-modality patterns (Figure 4). Between 2017 and 2025, TMS and tDCS showed the largest absolute increases, adding 1,193 and 722 annual publications, respectively. The corresponding increases were 160 for strict-core tFUS/TUS/LIFU and 53 for TI. In relative terms, however, TI had the highest CAGR (64.6%), followed by tFUS/TUS/LIFU (35.0%), tDCS (9.1%), and TMS (7.4%). Thus, TI combined the fastest proportional growth with the smallest absolute increase, whereas TMS showed the largest absolute expansion despite a much lower relative growth rate. This contrast emphasizes the influence of baseline field size when interpreting growth indicators.

**Figure 4:**
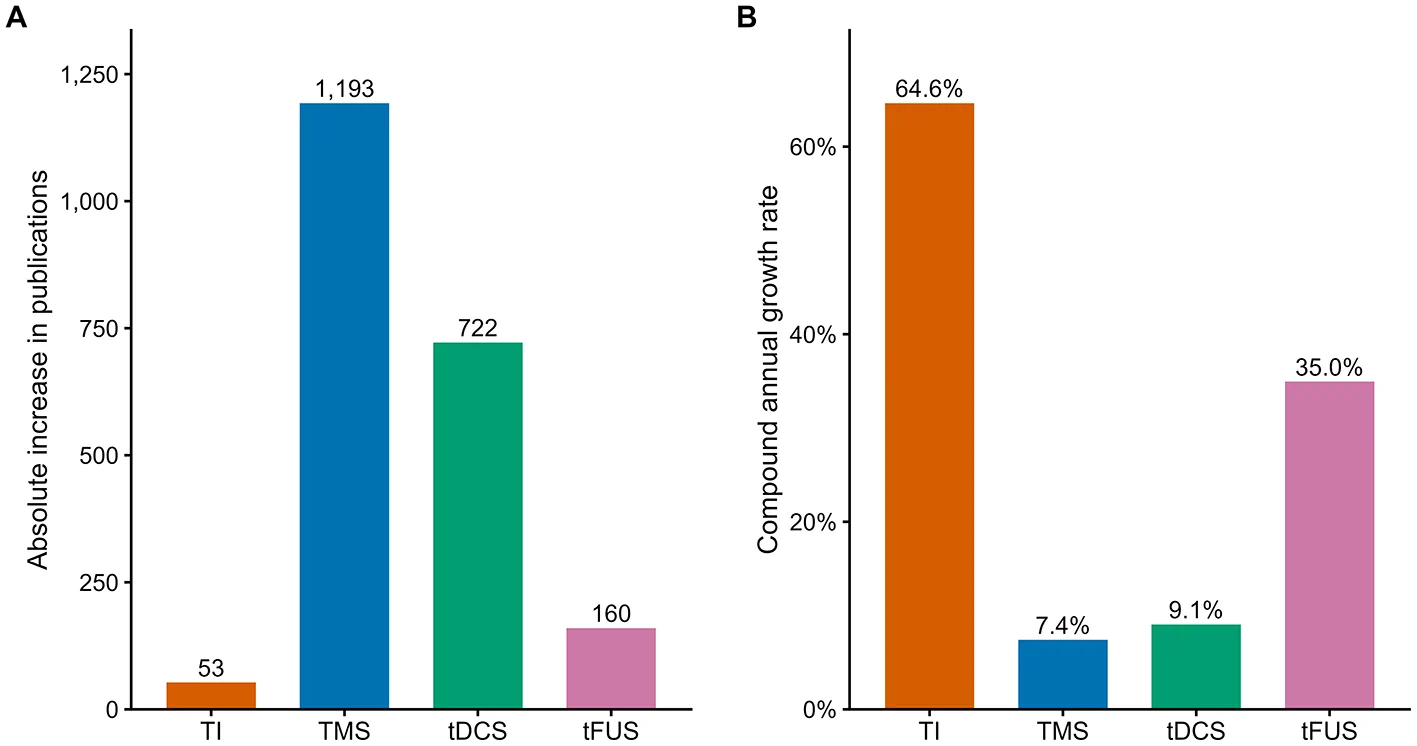
Absolute vs. relative growth of the four modalities from 2017 to 2025. **(A)** Absolute increase in annual publications between 2017 and 2025. **(B)** Compound annual growth rate (CAGR). TI showed the highest relative growth (64.6%) but the smallest absolute increase (53 publications), whereas TMS showed the largest absolute increase (1,193 publications) with a lower CAGR (7.4%). The CAGR of TI should be interpreted cautiously because its 2017 baseline consisted of only one record.

### 3.4 Geographic coverage and international collaboration structure

Geographic coverage differed substantially across the four modalities. TMS and tDCS involved the largest numbers of contributing countries, with 112 and 102 countries, respectively. Strict-core tFUS/TUS/LIFU involved 41 countries, whereas TI involved 29. Because absolute country counts are sensitive to corpus size, these raw differences were interpreted together with the size- and year-matched analysis (Figure 5; Table 1).

**Figure 5:**
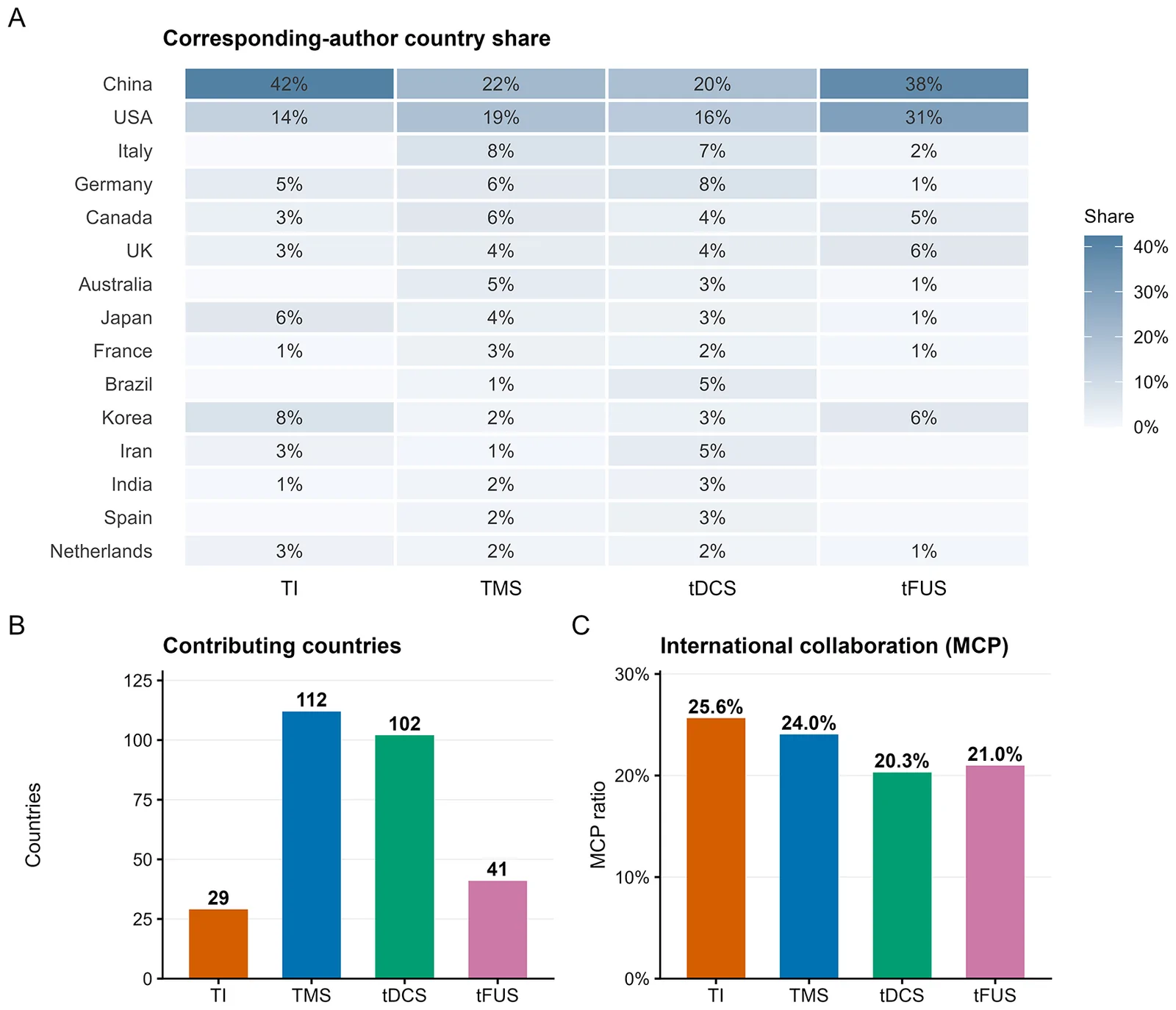
Geographic distribution and international collaboration indicators. **(A)** Shares of modality-specific publication output attributed to the corresponding author's country for the 15 leading countries. **(B)** Number of contributing countries identified from all authors' affiliation countries. **(C)** Multiple-country publication ratio (MCP), calculated among records with available country information as the proportion involving authors from at least two countries. The tFUS/TUS/LIFU values were calculated from the strict ultrasound-neuromodulation core set (*n* = 843). Because absolute country counts are sensitive to corpus size, these results were interpreted together with the size- and year-matched analysis in Figure 8.

The multiple-country publication ratio (MCP) was 25.6% for TI, 24.0% for TMS, 21.0% for strict-core tFUS/TUS/LIFU, and 20.3% for tDCS. Thus, despite its narrower geographic coverage in absolute terms, TI showed a multinational collaboration proportion comparable to or slightly higher than those of the larger comparison corpora. The distinction between country coverage and collaboration proportion is important: the larger TMS and tDCS literatures involved more countries overall, whereas TI did not show a correspondingly lower MCP.

### 3.5 Raw country collaboration networks differ in scale and connectivity

Country co-authorship networks differed markedly in their raw scale and visible connectivity. The larger TMS and tDCS datasets produced networks containing more countries and co-authorship links, whereas the strict-core tFUS/TUS/LIFU network showed an intermediate raw scale and the TI network contained fewer countries and links. Because network size and visible connectivity are sensitive to publication volume, these panels were interpreted descriptively and together with the size- and year-matched resampling results rather than as directly standardized cross-modality indicators. Nevertheless, the TI network already contained several cross-national links, indicating that international collaboration has begun to develop despite the relatively small literature base (Figure 6). Descriptive institution- and author-string publication-output profiles are provided in Supplementary Figures S2, S3. Because institutional names were not consolidated across parent and affiliated systems and author names were not disambiguated, these outputs were not used for inferential cross-modality comparisons.

**Figure 6:**
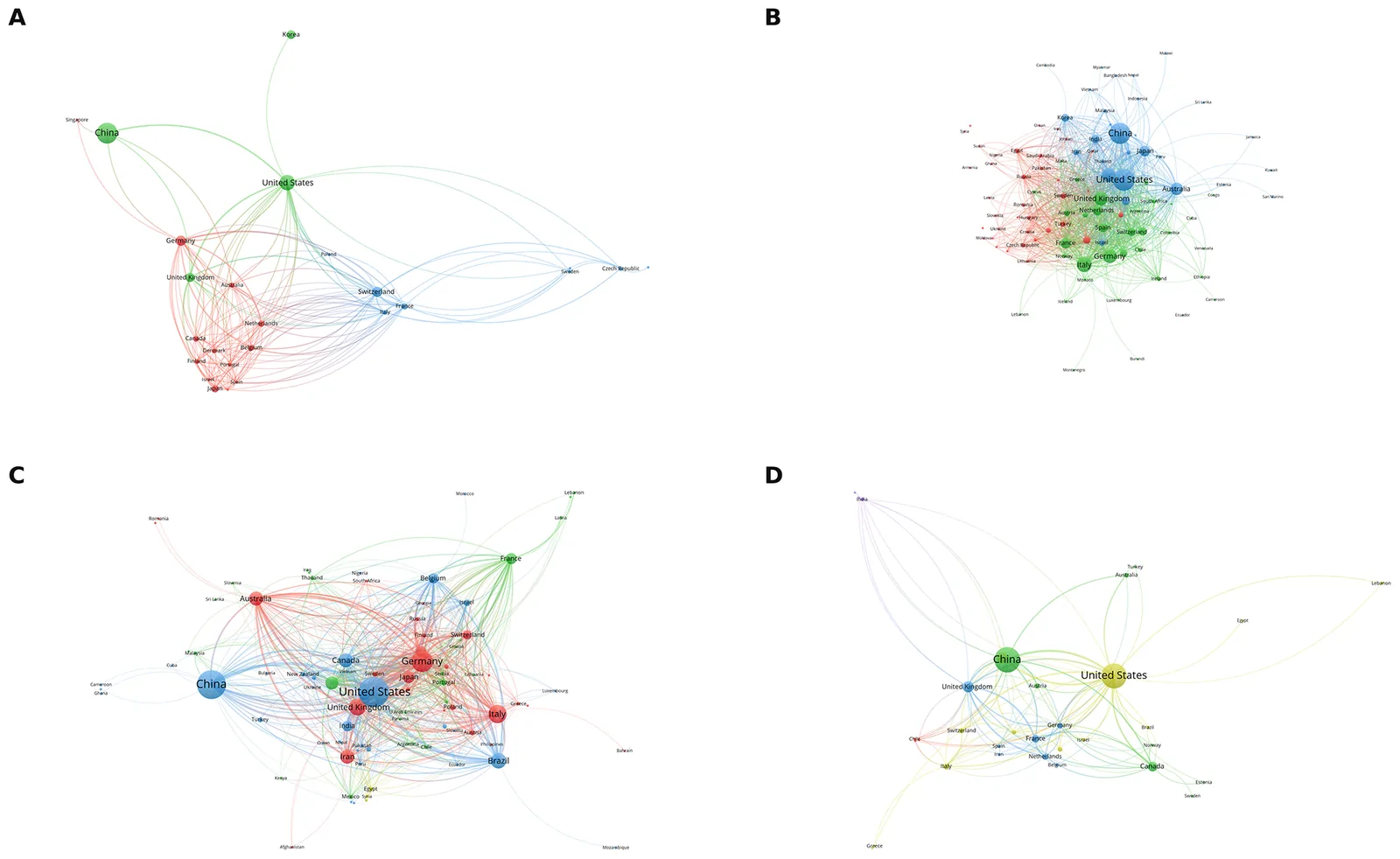
Country co-authorship networks for **(A)** TI, **(B)** TMS, **(C)** tDCS, and **(D)** strict-core tFUS/TUS/LIFU, visualized using VOSviewer. Node size indicates national publication output, and links indicate co-authorship relationships between countries. Colors represent VOSviewer clusters and do not imply a cross-modality developmental ordering. Because network scale and connectivity are affected by publication volume, the panels are interpreted descriptively rather than as directly standardized cross-modality comparisons.

### 3.6 Keyword co-occurrence networks reveal distinct thematic structures across modalities

Author keyword co-occurrence networks revealed clear differences in thematic structure across modalities. The TI network was more concentrated, mainly centered on technical mechanisms and deep-targeting themes such as temporal interference, non-invasive deep brain stimulation, electric fields, computational modeling, and human stimulation. This pattern indicates that TI research remains comparatively concentrated around technical principles, dose modeling, and human deep-target validation. In contrast, the TMS and tDCS networks were more diversified, covering clinical application and neuroplasticity-related themes such as depression, stroke, motor cortex, plasticity, and rehabilitation. The keyword structure of tFUS/TUS/LIFU (strict core set) showed a mixed profile centered on transcranial ultrasound stimulation, neuromodulation, transducers, and acoustics, together with disease-application terms such as Alzheimer's disease, depression, epilepsy, and Parkinson's disease. This profile combined acoustic/engineering foundations with emerging disease-oriented neuromodulation, rather than the blood–brain barrier and delivery themes of the broader focused-ultrasound field. Within the contemporary dataset, tFUS/TUS/LIFU therefore showed a mixed thematic structure combining engineering, mechanistic, and translational elements (Supplementary Figure S1).

### 3.7 Annual keyword heatmaps reveal thematic evolution trajectories

Whereas the co-occurrence networks summarized the overall thematic structure, the annual heatmaps were used to examine within-modality changes over time. For TI, early keywords were sparse and subsequently became increasingly concentrated around themes such as non-invasive deep brain stimulation, electric field, computational modeling, and human stimulation. This pattern suggests that the field has expanded from conceptual development and computational modeling toward human studies and deep-targeting validation. Given the small literature base of TI, however, year-to-year changes should be interpreted cautiously in the context of limited sample size (Figure 7A).

**Figure 7:**
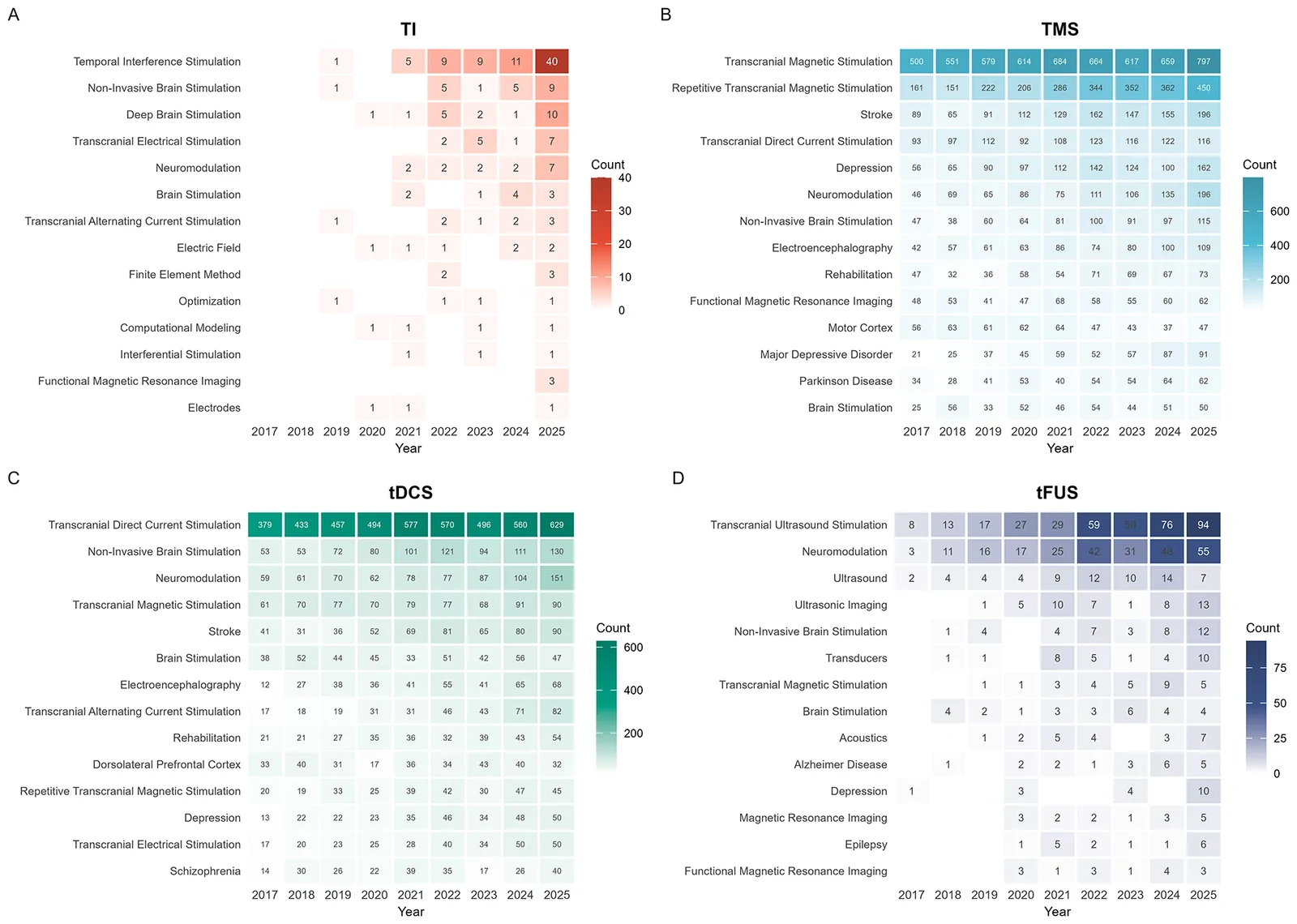
Annual heatmaps of standardized author keywords in **(A)** TI, **(B)** TMS, **(C)** tDCS, and **(D)** strict-core tFUS/TUS/LIFU from 2017 to 2025. Cell values represent annual frequencies of standardized author keywords, with modality-specific color scales. Selected modality-related terms were retained to show the co-evolution of technical terminology and application themes.

The annual keyword profile of TMS showed persistently high-frequency and highly diversified themes, with terms such as depression, motor cortex, plasticity, and rTMS appearing continuously across multiple years. tDCS similarly exhibited a diversified thematic structure with prominent clinical and rehabilitation-oriented topics, with recurring themes including stroke, depression, cognition, and rehabilitation. In contrast, tFUS/TUS/LIFU showed increasing prominence of terms such as transcranial ultrasound stimulation, neuromodulation, and transducers, together with disease-application terms including Alzheimer's disease, depression, and epilepsy, suggesting that its focus is expanding from acoustic-field physics and transduction toward disease-oriented neuromodulation (Figures 7B–D).

Overall, the annual keyword heatmaps were consistent with the keyword co-occurrence networks: TMS and tDCS displayed bibliometrically established and thematically diversified structures; tFUS/TUS/LIFU showed a mixed contemporary bibliometric profile with increasing translationally oriented research; and TI remained concentrated around a limited set of core technical and deep-targeting themes (Figure 7; Supplementary Figure S1).

### 3.8 Scopus-based co-citation networks reveal distinct knowledge-base structures

Cited-reference canonicalization reduced the number of distinct reference strings by 22.5% for TI, 41.4% for TMS, 37.7% for tDCS, and 31.8% for strict-core tFUS/TUS/LIFU. At the main minimum cited-reference occurrence thresholds, the TI, TMS, tDCS, and strict-core tFUS/TUS/LIFU networks contained 206, 253, 192, and 212 nodes, with 12,078, 26,448, 17,578, and 20,273 weighted co-citation edges, respectively. The corresponding highest-modularity Louvain partitions each contained four communities, with modularity values of 0.1106, 0.2450, 0.1158, and 0.0974, respectively (Figure 1; Supplementary Table S5).

Given the modest modularity values across the four main-threshold networks (0.0974–0.2450), these communities should be interpreted as coarse descriptive partitions of the co-citation structure rather than sharply separated intellectual modules. Within this constraint, the TI network remained centered on a relatively limited group of foundational biophysical, computational-modeling, dose-optimization, and human-validation references. The TMS network incorporated guideline, safety, theta-burst stimulation, and clinical-treatment foundations, whereas the tDCS network combined foundational physiological studies with safety, methodological, and application-oriented references. The strict-core tFUS/TUS/LIFU network showed a mixed knowledge structure combining foundational ultrasound-neuromodulation studies, mechanistic and parameter-oriented work, and human and non-human-primate validation.

Threshold sensitivity analyses supported the stability of the principal intellectual bases. For TI, 19 and 20 of the 20 references with the highest total co-citation link strength under the main threshold remained within the corresponding Top-20 rankings under the lower and higher thresholds, respectively. The corresponding retention counts were 19 and 19 for TMS, 20 and 19 for tDCS, and 19 and 20 for strict-core tFUS/TUS/LIFU. These results indicate that the highest-link-strength references were not dependent on a single threshold choice (Figure 1; Supplementary Table S5).

### 3.9 Core highly cited documents reveal modality-specific intellectual foundations

Whereas reference co-citation analysis maps the intellectual foundations cited by the included literature, source-document citation analysis identifies influential publications within each modality-specific corpus. Highly cited source documents further reflected differences in knowledge-base structure across modalities. To provide a single, internally consistent metric, the representative documents were ranked by Web of Science citation counts within the Web of Science–indexed subset and are listed in Table 2 and visualized in Supplementary Figure S4; core-adjacent records identified during manual relevance annotation are provided in Supplementary Table S7. For TI, the core highly cited documents mainly comprised foundational and modeling studies such as Grossman 2017, Mirzakhalili 2020, Rampersad 2019, and Esmaeilpour 2021. For tFUS/TUS/LIFU, the core documents were primarily reviews of ultrasound neuromodulation and human or non-human primate studies of deep-targeted stimulation, based on the strict neuromodulation core set (Table 2).

**Table 2:** Representative highly cited core documents in each modality-specific dataset.

Modality	First author (year)	Journal	Title	WoS citations
TI	Grossman N (2017)	*Cell*	Noninvasive deep brain stimulation via temporally interfering electric fields	720
TI	Mirzakhalili E (2020)	*Cell Systems*	Biophysics of temporal interference stimulation	144
TI	Rampersad S (2019)	*NeuroImage*	Prospects for transcranial temporal interference stimulation in humans: A computational study	126
TI	Esmaeilpour Z (2021)	*Brain Stimulation*	Temporal interference stimulation targets deep brain regions by modulating neural oscillations	107
TI	Lee S (2020)	*Scientific Reports*	Individually customized transcranial temporal interference stimulation for focused modulation of deep brain structures: A simulation study with different head models	85
TMS	Lefaucheur J (2020)	*Clinical Neurophysiology*	Evidence-based guidelines on the therapeutic use of repetitive transcranial magnetic stimulation (rTMS): An update (2014–2018)	1,761
TMS	Rossi S (2021)	*Clinical Neurophysiology*	Safety and recommendations for TMS use in healthy subjects and patient populations, with updates on training, ethical and regulatory issues: Expert guidelines	1,120
TMS	Blumberger D (2018)	*Lancet*	Effectiveness of theta burst vs. high-frequency repetitive transcranial magnetic stimulation in patients with depression (THREE-D): A randomized non-inferiority trial	961
TMS	Cole E (2020)	*American Journal of Psychiatry*	Stanford accelerated intelligent neuromodulation therapy for treatment-resistant depression	552
TMS	Cole E (2022)	*American Journal of Psychiatry*	Stanford neuromodulation therapy (SNT): A double-blind randomized controlled trial	523
tDCS	Lefaucheur J (2017)	*Clinical Neurophysiology*	Evidence-based guidelines on the therapeutic use of transcranial direct current stimulation (tDCS)	1,358
tDCS	Antal A (2017)	*Clinical Neurophysiology*	Low intensity transcranial electric stimulation: Safety, ethical, legal regulatory and application guidelines	992
tDCS	Vöröslakos M (2018)	*Nature Communications*	Direct effects of transcranial electric stimulation on brain circuits in rats and humans	533
tDCS	Fregni F (2021)	*International Journal of Neuropsychopharmacology*	Evidence-based guidelines and secondary meta-analysis for the use of transcranial direct current stimulation in neurological and psychiatric disorders	435
tDCS	Liu A (2018)	*Nature Communications*	Immediate neurophysiological effects of transcranial electrical stimulation	413
tFUS	Blackmore J (2019)	*Ultrasound in Medicine and Biology*	Ultrasound neuromodulation: A review of results, mechanisms and safety	421
tFUS	Folloni D (2019)	*Neuron*	Manipulation of subcortical and deep cortical activity in the primate brain using transcranial focused ultrasound stimulation	300
tFUS	Legon W (2018)	*Human Brain Mapping*	Neuromodulation with single-element transcranial focused ultrasound in human thalamus	258
tFUS	Darmani G (2022)	*Clinical Neurophysiology*	Non-invasive transcranial ultrasound stimulation for neuromodulation	250
tFUS	Legon W (2018)	*Scientific Reports*	Transcranial focused ultrasound neuromodulation of the human primary motor cortex	249

Citation counts are Web of Science Core Collection counts and were not combined with Scopus. Representative documents were selected from the Web of Science-indexed subset of each modality-specific corpus and ranked using the Web of Science TC field. For tFUS/TUS/LIFU, ranking used the strict neuromodulation core set (n = 843). Additional highly cited core and core-adjacent documents are reported in Supplementary Table S7.

Consistent with the broader contemporary thematic profiles of TMS and tDCS, the core-adjacent highly cited records for these modalities (Supplementary Table S7) included not only technical guidelines and safety-consensus documents but also numerous disease-oriented reviews and clinical guidelines, covering topics such as depression, bipolar disorder, Parkinson's disease, and traumatic brain injury. Because the TMS and tDCS literatures encompass broader application scenarios and disease coverage, their highly cited literature is more strongly influenced by adjacent disease fields and clinical evidence. By comparison, the highly cited documents of the emerging modalities (TI and tFUS/TUS/LIFU) remained focused on the technologies themselves (Table 2; Supplementary Table S7).

### 3.10 Size- and year-matched resampling distinguishes persistent thematic differences from scale-sensitive indicators

Across 10,000 size- and year-matched resamples based on the final TI corpus of 162 records, TI showed lower author-keyword Shannon diversity and higher Top-10 keyword concentration than matched TMS, tDCS, and strict-core tFUS/TUS/LIFU samples. All six thematic comparisons remained significant after joint Benjamini–Hochberg correction (adjusted empirical *P* < 0.001), indicating that TI's greater thematic concentration was not explained by corpus size or annual publication composition within the contemporary observation window.

Several non-thematic indicators showed weaker or inconsistent differences after matching. TI involved fewer contributing countries than matched TMS samples (adjusted *P* = 0.045), whereas the comparison with tDCS did not remain significant after multiple-testing adjustment (adjusted *P* = 0.051), and no difference was observed relative to strict-core tFUS/TUS/LIFU. Mean authors per publication differed only between TI and tDCS (adjusted *P* = 0.045). MCP ratio and mean author keywords per publication did not differ significantly between TI and any comparison modality after adjustment.

These findings indicate that TI's greater thematic concentration was the most consistent cross-modality difference after controlling for corpus size and annual composition, whereas geographic and collaboration-related indicators were more sensitive to the comparison modality and multiple-testing adjustment (Figure 8; Supplementary Table S10).

**Figure 8:**
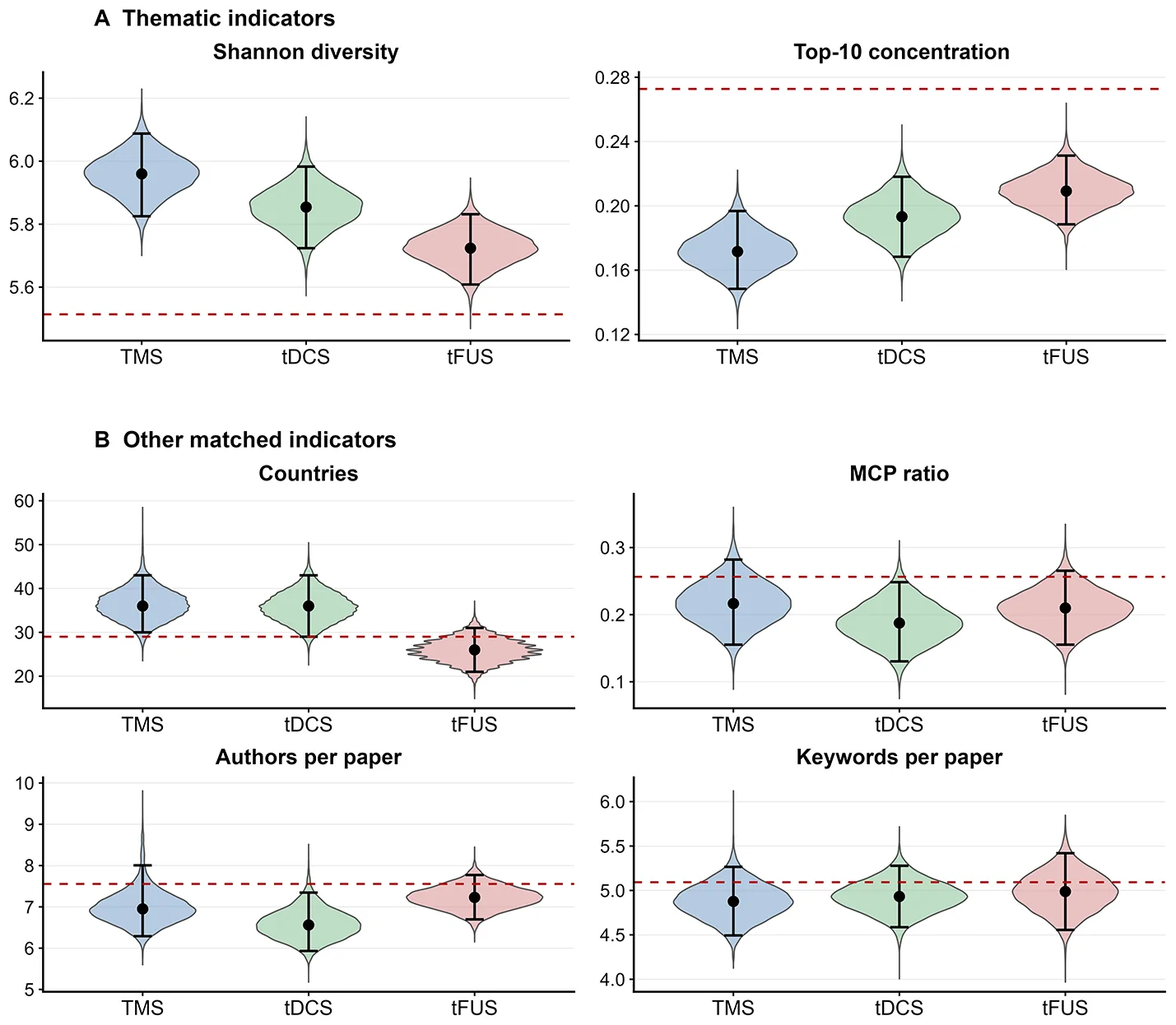
Size- and year-matched resampling analysis of bibliometric indicators across modalities. TI comprised all 162 eligible records. For TMS, tDCS, and strict-core tFUS/TUS/LIFU, 162 records were sampled without replacement within publication-year strata to reproduce the annual distribution of TI. The procedure was repeated independently 10,000 times using a fixed random seed. Violin distributions represent empirical resampling distributions; black points indicate medians, whiskers indicate the 2.5th−97.5th percentile intervals, and red dashed lines indicate observed TI values. **(A)** TI showed lower author-keyword Shannon diversity and higher Top-10 keyword concentration than all three comparison modalities, with all six thematic comparisons remaining significant after joint Benjamini–Hochberg correction (adjusted empirical *P* < 0.001). **(B)** TI involved fewer contributing countries than matched TMS samples (adjusted *P* = 0.045), whereas the comparison with tDCS did not remain significant after adjustment (adjusted *P* = 0.051), and no difference was observed relative to strict-core tFUS/TUS/LIFU. Mean authors per publication differed only between TI and tDCS (adjusted *P* = 0.045). MCP ratio and mean author keywords per publication did not differ significantly in any comparison.

### 3.11 Sensitivity analyses support the robustness of the thematic findings

The revised TI corpus comprised 162 unique records after expanded-search adjudication and targeted re-audit. Repeating the primary matched thematic analysis with this corpus preserved all six statistically significant contrasts: TI showed lower author-keyword Shannon diversity and higher Top-10 keyword concentration than TMS, tDCS, and strict-core tFUS/TUS/LIFU. The same directions and statistical significance were retained after harmonization of modality-family keyword variants and established subtype labels (Supplementary Table S12).

The thematic pattern was also preserved under alternative inclusion and measurement definitions. In the title-only sensitivity analysis, all six contrasts remained significant after Benjamini–Hochberg adjustment (Supplementary Table S16). When the same prespecified title-term procedure used in the historical analysis was applied to the contemporary corpora, TI again showed lower Shannon diversity and higher Top-10 concentration than all three matched comparators, indicating that the contemporary ordering was not dependent on author keywords as the thematic measurement instrument (Supplementary Table S17).

Expanding the comparator corpora to 20,356 TMS records and 850 strict-core tFUS/TUS/LIFU records, with tDCS unchanged at 10,576 records, likewise preserved all six thematic contrasts, with all adjusted *P* < 0.001 (Supplementary Table S19). Recalculation using two-sided empirical *P* values also left all six comparisons significant in both the primary and expanded-comparator analyses after correction across the corresponding 18-test families; the largest adjusted *P* was 0.002 (Supplementary Table S20).

Taken together, all five complementary contemporary robustness analyses preserved both the direction and statistical significance of the six thematic contrasts, including under an alternative inclusion definition in which the TI corpus was reduced from 162 to 121 title-defined records. The same conclusions were also retained under alternative thematic representations, including modality-family keyword harmonization and title-term measurement. Broadening the historical tFUS corpus from 76 strict-core records to 123 records by including core-adjacent studies likewise preserved the non-monotonic field-age pattern (Supplementary Table S18).

### 3.12 Manual relevance audit and inter-reviewer agreement

The publication-year-stratified manual relevance audit identified 81 modality-core records among 100 sampled TI records, 146 among 200 TMS records, and 147 among 200 tDCS records. The corresponding design-weighted core proportions were 81.2% for TI (95% CI, 76.8%−85.6%), 72.7% for TMS (95% CI, 66.5%−78.9%), and 73.4% for tDCS (95% CI, 67.3%−79.5%). The weighted and unweighted estimates were closely aligned. The unweighted core proportions were 81.0% (81/100; Wilson 95% CI, 72.2%−87.5%) for TI, 73.0% (146/200; Wilson 95% CI, 66.5%−78.7%) for TMS, and 73.5% (147/200; Wilson 95% CI, 67.0%−79.1%) for tDCS. Overall, 374 of the 500 audited records were classified as modality-core; this pooled value was reported descriptively because the three modality-specific samples had different sampling fractions.

The principal reasons for non-core classification differed across modalities. Incidental mention of the target modality was the most frequent reason among non-core TMS records, whereas classification as another stimulation modality was the most frequent reason among non-core tDCS records. Among the 19 non-core TI records, nine were classified as other-modality records, six involved substantive TI applications outside the prespecified transcranial brain-neuromodulation scope, two reflected incidental mention of TI, and two represented non-neuromodulation or terminology/abbreviation mismatches. The out-of-scope TI records mainly involved peripheral-nerve or ocular/retinal stimulation. Detailed TI exclusion categories, together with the modality-level audit and inter-reviewer agreement results, are reported in Supplementary Table S13.

Before consensus discussion, the two reviewers agreed on 87 of the 90 independently coded records, yielding an overall agreement of 96.7% and a Cohen's κ of 0.902 (95% CI, 0.793–1.000). The three disagreements concerned one tDCS record and two TI records. These disagreements were resolved through consensus using the predefined modality-specific scope rules and additional abstract, full-text, or publisher-record verification.

These findings indicate that modality-core records constituted the majority of each audited dataset. Nevertheless, residual scope heterogeneity remained in all three broad-scope datasets and was more pronounced in the audited TMS and tDCS samples than in the TI sample. The audit results were used to characterize residual dataset heterogeneity and refine the reporting of exclusion reasons; they were not used to proportionally remove records from, or recalculate, the full bibliometric datasets. Because the audit preceded the targeted corpus corrections introduced during revision, its weighted estimates characterize the pre-revision common-screening corpora rather than the final corrected modality-specific datasets.

### 3.13 Field-age-aligned sensitivity analysis reveals non-monotonic thematic structure

The strict field-age-aligned corpora contained 439 TMS, 158 tDCS, 76 tFUS, and 99 TI records. In the full corpora, Shannon diversity was 5.5959 for TMS, 5.3164 for tDCS, 5.2860 for tFUS, and 5.2716 for TI, while C10 was 0.2847, 0.2583, 0.1636, and 0.2053, respectively (Supplementary Table S14). Because these estimates remain sample-size dependent, the equal-N rarefaction was treated as the primary sensitivity comparison. At *n* = 76, median Shannon diversity was 5.1385 (2.5th−97.5th percentile: 5.0532–5.2121) for TI, 4.9918 (4.8300–5.1404) for TMS, 4.9802 (4.8376–5.1067) for tDCS, and 5.2860 for the fixed tFUS reference. Corresponding C10 values were 0.2100 (0.1890–0.2310), 0.2910 (0.2506–0.3333), 0.2668 (0.2353–0.3011), and 0.1636, respectively (Supplementary Figure S5). Thus, field-age and sample-size alignment did not yield a monotonic thematic ordering across field-age-aligned corpora. Early TMS and tDCS remained more concentrated than TI, whereas early tFUS showed the lowest Top-10 concentration. The Shannon resampling ranges for TI, TMS, and tDCS overlapped, further arguing against a simple monotonic field-age interpretation. Taken together, the two resampling analyses address different sources of non-comparability: the contemporary analysis in Figure 8 controls corpus size and annual composition within 2017–2026, whereas Supplementary Figure S5 additionally aligns field age using historically reconstructed modality-specific corpora.

Importantly, when the same title-term measurement instrument was applied to the contemporary corpora, TI remained more thematically concentrated than matched TMS, tDCS, and tFUS/TUS/LIFU samples, whereas the field-age-aligned historical comparison remained non-monotonic. The broader historical tFUS sensitivity analysis (*n* = 123 before equal-N resampling) produced the same qualitative historical pattern. Together, these findings indicate that the contrast between the contemporary and field-age-aligned results cannot be attributed simply to the use of different thematic measurement instruments or to the stricter historical tFUS screening boundary.

## 4 Discussion

This study systematically compared the literature on four non-invasive brain stimulation modalities—TI, TMS, tDCS, and tFUS/TUS/LIFU—from 2017 to 2026 and supplemented this contemporary comparison with a field-age-aligned historical sensitivity analysis. Across publication growth, geographic distribution, collaboration structure, thematic evolution, co-citation networks, and highly cited source documents, the four modalities showed distinct patterns of literature accumulation and knowledge organization. Importantly, although the contemporary analyses identified substantial cross-modality differences, the field-age-aligned analysis did not reproduce a monotonic field-age ordering. The findings therefore support a multidimensional interpretation of cross-modality bibliometric differences rather than a single common developmental ordering. The following sections discuss these modality-specific knowledge structures, the characteristics of TI as an emerging modality, and future directions for integration, standardization, and clinical translation.

### 4.1 Non-invasive brain stimulation modalities show distinct bibliometric knowledge structures

Within the common 2017–2026 observation window, the four modalities showed distinct bibliometric structures. TMS and tDCS had the largest and most diversified contemporary literatures, TI remained comparatively concentrated around technical principles, modeling, dose optimization, and deep-target validation, and strict-core tFUS/TUS/LIFU showed a mixed engineering–mechanistic–translational profile. These differences describe contemporary knowledge organization but should not by themselves be interpreted as successive positions along a single developmental axis.

The size- and year-matched contemporary analysis further showed that not all differences were attributable to corpus scale. After matching the comparison modalities to the 162-record annual distribution of TI, TI retained lower author-keyword Shannon diversity and higher Top-10 keyword concentration than TMS, tDCS, and strict-core tFUS/TUS/LIFU, whereas several geographic and collaboration indicators were attenuated. Thus, TI's contemporary thematic concentration is not explained by present-window publication volume alone. This pattern was also retained after expansion of the comparator corpora and after recalculating the thematic comparisons with two-sided empirical tests (Supplementary Tables S19, S20).

Importantly, the contemporary thematic pattern was also reproduced when the same prespecified title-term measurement procedure used for the historical analysis was applied to the contemporary corpora, indicating that the contrast between the contemporary and field-age-aligned results was not simply an artifact of using different thematic measurement instruments. However, the field-age-aligned analysis materially changes how these contemporary differences should be interpreted. When each modality was examined over the corresponding nine consecutive calendar-year window anchored to its field-defining modern introduction year and corpus size was standardized to *n* = 76, the modalities did not reproduce a monotonic field-age ordering. Early TMS and tDCS showed greater Top-10 concentration than TI, whereas early tFUS showed the lowest concentration, and the Shannon diversity ranges for TI, TMS, and tDCS overlapped. Field age therefore contributes to present-day bibliometric structure but does not by itself define a universal thematic trajectory across modalities. The contemporary and field-age-aligned analyses are complementary rather than contradictory: the former tests whether TI's present-day structure is explained by current corpus size and annual composition, whereas the latter tests whether the same age-related pattern is reproduced across modalities.

We therefore use the term bibliometric knowledge-structure spectrum as a neutral descriptor of the observed cross-modality differences. A modality's position within this spectrum reflects a combination of historical depth, publication volume, technical traditions, target populations, experimental paradigms, and translational pathways. This formulation preserves the robust descriptive differences identified in the contemporary analysis while avoiding the stronger assumption that all NIBS technologies follow the same bibliometric trajectory.

### 4.2 TI shows the highest relative growth from a small baseline and retains a concentrated thematic structure

TI showed the highest relative growth (CAGR, 64.6%) but the smallest absolute increase (53 publications), whereas TMS showed the lowest relative growth (7.4%) but the largest absolute increase (1,193 publications). This combination reflects rapid proportional expansion from a very small publication base and is consistent with TI's status as an emerging modality. At the same time, TI's keyword structure, co-citation network, and highly cited documents remained comparatively concentrated around a limited group of technical, biophysical, and deep-targeting themes (Figures 1, 4, 7; Supplementary Figure S1; Table 2).

This concentration should not be interpreted as evidence that TI remains confined to modeling or initial feasibility studies. Since the original demonstration of temporal interference stimulation, the field has expanded from computational and preclinical work to human deep-target studies involving the hippocampus and striatum, and more recent investigations have extended into patient populations, including Parkinson's disease and epilepsy (Lamoš et al., 2025; Yang et al., 2025, 2026; Missey et al., 2026; Xu et al., 2026). Some of these studies have incorporated direct deep-target physiological validation using intracranial or depth-electrode recordings. TI therefore remains an emerging and comparatively concentrated field, but its translational literature now extends beyond healthy-volunteer proof-of-concept work.

Several TI-related indicators should nevertheless be interpreted cautiously because of the small sample size. The 2017 baseline consisted of only one record, making the CAGR and normalized growth index sensitive to fluctuations in individual years (Figures 3, 4). Similarly, the relatively high proportion of unique TI records identified through expanded searching and PubMed gap-checking reflected a mixture of abbreviation-driven marginal matches, database-indexing differences, and genuinely relevant records not captured by the original main search (Supplementary Tables S2, S6). Substantively TI-relevant records identified through targeted adjudication were incorporated into the final revised corpus (Supplementary Tables S2, S3). These findings suggest that the interpretation of TI should focus on its structural characteristics and longitudinal context rather than on the absolute magnitude of individual numerical indicators. Despite these limitations, TI already showed an international collaboration proportion comparable to those observed in the much larger TMS and tDCS datasets, with an MCP of 25.6%, and had formed several cross-national collaboration links, suggesting increasing international engagement despite its relatively small literature base.

### 4.3 tFUS/TUS/LIFU shows a mixed contemporary bibliometric profile among deep-targeting strategies

Within the 2017–2026 contemporary dataset, 843 of the 1,100 broad-scope ultrasound records met the strict neuromodulation definition. After exclusion of blood–brain barrier opening, drug delivery, ablation, tumor treatment, and non-neuromodulation imaging or engineering records, the strict-core corpus retained a mixed profile combining sustained publication growth, acoustic engineering, neural-interaction mechanisms, targeting methodology, and disease-oriented applications. Its most highly cited core documents were also dominated by genuine neuromodulation reviews and human or non-human primate stimulation studies. These findings indicate that the mixed contemporary bibliometric profile of tFUS/TUS/LIFU was not an artifact of including the broader focused-ultrasound literature. The field-age-aligned analysis further showed that early tFUS did not occupy a simple intermediate position in thematic diversity or concentration, reinforcing interpretation of cross-modality differences as a spectrum of modality-specific knowledge structures rather than a uniform cross-modality pattern. The historical tFUS corpus was subjected to more intensive record-level screening than the other field-age corpora because ultrasound terminology overlaps extensively with non-neuromodulation applications. This asymmetry should be considered when interpreting cross-modality historical comparisons. However, broadening the historical tFUS definition to include core-adjacent ultrasound-neuromodulation records increased the corpus from 76 to 123 records without changing the qualitative non-monotonic field-age pattern.

### 4.4 Comparison with previous bibliometric studies

Previous bibliometric work on non-invasive brain stimulation has largely been organized around a single modality or a single disease. For the established modalities, whole-field analyses of TMS (Deng et al., 2025) and tDCS (Sun et al., 2022), together with disease-specific mappings such as TMS in depression (Yang et al., 2024) and tDCS in stroke (Zhou et al., 2023), show that these fields already support a multi-layered bibliometric literature extending from whole-field trends to individual clinical indications. For the emerging modalities, dedicated analyses have only recently appeared: a bibliometric analysis of transcranial ultrasound stimulation (Wang et al., 2025) and, for temporal interference, single-modality mappings emphasizing clinical translation and global research trends (Lin et al., 2026; Qi et al., 2026). Qi et al. (2026) was published after our database search (June 20–21, 2026) and therefore could not be included in the analytic dataset; it is discussed here only as the most recent prior work. Cross-technique studies remain scarce and have so far pooled multiple NIBS techniques within a single disease context, such as stroke (Li et al., 2025), rather than treating the modalities as independent comparison units.

The present study extends these earlier bibliometric mappings in four main respects: it directly compares four modalities within a common contemporary observation window; applies a strict ultrasound-neuromodulation core definition; formally evaluates contemporary corpus-size and annual-composition effects through matched resampling; and, importantly, reconstructs modality-specific early historical windows to examine field-age confounding. The present analysis therefore compares contemporary and field-age-aligned knowledge structures under complementary controls without assuming a common cross-modality trajectory.

### 4.5 Implications for future integration, standardization, and clinical translation

The cross-modality differences identified in this study imply distinct research priorities across NIBS modalities. For TMS and tDCS, the central challenge is no longer simply to expand publication volume or demonstrate technical feasibility, but to improve study quality and reproducibility within large and diversified evidence bases and to integrate application evidence across diseases. For TI, its contemporary literature structure points to different priorities: broadening the evidence base beyond a concentrated set of foundational studies, expanding collaborative networks, and improving methodological and reporting standardization. These differences reflect modality-specific research and translational contexts rather than a common temporal progression.

The structural findings of this study suggest several directions for the future integration of TI research. First, multicenter collaboration will be essential. The limited absolute geographic coverage of TI, despite an international collaboration proportion comparable to those of the larger modalities, supports broader multicenter participation to increase sample sizes and evaluate reproducibility across settings. Second, standardization is needed. As TI depends strongly on electric field interference and dose modeling, the standardization of stimulation parameters, modeling approaches, and safety assessment will improve the comparability of findings across studies. Third, clinical translation should be further developed. For TI, future priorities should increasingly shift from demonstrating that deep targets can be modulated toward establishing reproducibility, target specificity, dose–response relationships, multisession durability, safety, and clinically meaningful efficacy in adequately powered controlled patient cohorts. The emergence of Parkinson's disease and epilepsy studies, together with direct physiological validation of deep targets, indicates that the field has already begun this translational transition. The next challenge is therefore not simply to generate additional proof-of-concept studies, but to determine whether observed physiological or behavioral effects are reproducible across centers, stimulation configurations, and patient populations. Several patient trials incidentally identified during literature screening are also registered, including studies in major depressive disorder, bipolar depression, and drug-resistant epilepsy (e.g., NCT06461260, NCT06516991, NCT06708143, and NCT07021508). These registrations are cited as contextual examples of ongoing translational activity; they were not identified through a systematic trial-registry review, were not included in the bibliometric analytic corpus, and should not be interpreted as evidence of clinical efficacy. For tFUS/TUS/LIFU, its mixed contemporary bibliometric profile suggests that closer integration between neuromodulation-oriented research and translational therapeutic applications, together with standardized acoustic parameter reporting, image guidance, and targeting accuracy, will be important for its further development (Murphy et al., 2025).

It is also important to emphasize that standardization involves not only stimulation parameters and modeling procedures, but also terminology. The TI field still uses multiple expressions, including temporal interference, TI, TIS, tTIS, and transcranial temporal interference stimulation. In the present study, the inclusion of abbreviations such as tTIS and TIS in the expanded search indeed introduced additional marginal matches (Supplementary Table S2). Future convergence in terminology, stimulation-parameter reporting, electric field modeling, electrode configuration, safety assessment, and outcome reporting would improve comparability across studies and facilitate multicenter collaboration and clinical translation. On this basis, the longitudinal development of TI itself could be monitored. Potential indicators may include terminology convergence, increasing multicenter structure in collaboration networks, greater diversification of the co-citation knowledge base, growth in clinically relevant keywords, and the emergence of guideline or consensus documents.

The field-age analysis also argues against treating the historical development of TMS or tDCS as a default roadmap for TI. Early TMS and tDCS were more thematically concentrated than TI over its first 9 years, whereas early tFUS showed the opposite pattern, indicating that thematic diversification does not follow a fixed trajectory across stimulation modalities. Research priorities for TI should therefore be guided by its own technical and translational bottlenecks—particularly target-engagement validation, dose definition, multisession reproducibility, safety, and patient-level efficacy—rather than by an assumption that it will naturally reproduce the historical expansion pattern of established cortical stimulation technologies.

### 4.6 Limitations

This study has several limitations. First, bibliometric analyses depend on database coverage. Although WoS and Scopus provide broad coverage, conference papers, preprints, and non-English publications may still have been missed. Final PubMed gap-checking showed that 15,786 of 17,733 relevant PubMed records were covered by the WoS–Scopus datasets, corresponding to an overall coverage rate of 89.0%. Nevertheless, the influence of database indexing delays on the most recent studies cannot be completely excluded (Supplementary Table S6).

Second, relevance screening was based on title and author-keyword matching, which may have omitted records in which the target modality appeared only in the abstract and may have left residual scope heterogeneity in the retained corpora. Full-dataset screening and primary classification of the 500-record stratified audit were conducted by GW; a 90-record subset was independently classified by a second reviewer, yielding 96.7% agreement and Cohen's κ = 0.902. Although modality-core records constituted the majority of the audited TI, TMS, and tDCS samples, full manual screening of the much larger TMS and tDCS corpora was not feasible. In contrast, tFUS/TUS/LIFU underwent complete strict classification because of its substantial overlap with non-neuromodulation ultrasound applications, producing an unavoidable asymmetry in screening intensity across modalities. Historical tFUS records likewise required more extensive record-level adjudication; however, broadening the historical tFUS definition preserved the qualitative field-age pattern. All records added to or removed from the modality-specific corpora during revision underwent targeted record-level verification, but the original 500-record probabilistic audit was not repeated. Accordingly, the TI, TMS, and tDCS networks and thematic structures should be interpreted as modality-associated bibliometric landscapes rather than fully purified modality-core corpora.

Third, reference co-citation analysis was based on Scopus-only data, whereas other analyses were performed using the merged WoS–Scopus dataset. This means that the document scope differed slightly between co-citation analysis and the other bibliometric analyses. This choice was made to avoid node splitting caused by inconsistencies in cross-database cited-reference formatting, and the availability of structured cited-reference lists in Scopus was high across all four modalities (>98.7%). Nevertheless, the co-citation results should be interpreted within the context of Scopus-indexed reference data (Supplementary Tables S4, S5). Cited-reference canonicalization relied on the normalized first-author field, publication year, and normalized title information extracted from the Scopus cited-reference strings because standardized DOIs were not consistently available in the exported cited-reference fields. References whose title information could not be reliably extracted were conservatively retained using the normalized complete reference string rather than being automatically merged. Consequently, a small degree of residual reference-string fragmentation or rare false-positive consolidation cannot be completely excluded. In addition, highly cited source documents reflect citation influence within each modality-specific corpus and should not be interpreted as a definitive ranking of the most important or foundational contributions to that modality.

Fourth, the contemporary matched-resampling design controlled sample size and annual publication composition but did not eliminate all structural differences between the modality-specific literatures. The 162 TI records represent the complete contemporary TI corpus, whereas the matched TMS, tDCS, and tFUS/TUS/LIFU sets were subsamples drawn from substantially larger literatures with broader accumulated thematic and keyword pools. The resulting empirical comparisons are therefore conditional on the observed TI corpus and test whether equivalently sized samples drawn from the current comparator knowledge structures differ from TI; they do not reconstruct what the comparator fields looked like when their complete literatures were themselves of comparable size. This complementary question was addressed by the field-age-aligned analysis, in which early TMS and tDCS were more concentrated than TI over its first 9 years, whereas early tFUS was less concentrated. Thus, small corpus size alone cannot mechanically explain the observed contemporary thematic ordering. Nevertheless, the matched analysis did not control for differences in disease distribution, study design, research stage, or clinical/preclinical composition, and should therefore be interpreted jointly with the field-age analysis rather than as a complete control for cross-modality heterogeneity. In addition, the thematic indicators used in this study are vocabulary-based proxies rather than direct semantic measurements of research content. The contemporary title-term sensitivity analysis showed that the main ordering was not dependent on author keywords alone, but this cross-validation does not substitute for content-level topic coding or semantic topic modeling.

Finally, the common 2017 starting year remains an important limitation of the primary analysis because it truncates the earlier histories of TMS, tDCS, and tFUS/TUS/LIFU. We partly addressed this temporal asymmetry through the added 9-year field-age-aligned sensitivity analysis. However, the historical comparison itself has limitations. The selected temporal anchors represent field-defining modern introductions rather than uniquely determined “birth dates” of each technology; publication and indexing practices differ substantially across the 1985–2025 period; and terminology changed over time, particularly in early TMS and ultrasound research. Historical differences in title length and reporting conventions may also influence title-term diversity despite standardized preprocessing. Older indexed corpora may also incompletely represent research activity that was published outside the coverage of these databases. The contemporary and historical thematic analyses also differed initially in their primary textual source, with author keywords used for the contemporary analysis and title terms used for the historical analysis. We therefore repeated the contemporary analysis using the same prespecified title-term instrument, and the qualitative contemporary ordering was preserved. This sensitivity analysis reduces, but does not entirely remove, concerns related to differences in historical metadata availability and textual reporting conventions. We mitigated these issues by using historically expanded retrieval terminology, complete record-level relevance assessment, standardized title terms rather than incompletely indexed historical author keywords, and equal-N rarefaction. The field-age analysis should therefore be interpreted as a complementary sensitivity analysis rather than a replacement for the contemporary 2017–2026 dataset. In addition, 2026 was an incomplete publication year. Although growth indicators such as CAGR and absolute growth were calculated using complete publication years from 2017 to 2025, the inclusion of records indexed through June 2026 in the overall dataset size and cross-sectional analyses may still be affected by search timing and database-indexing delays.

## 5 Conclusions and perspectives

Through a comparative bibliometric analysis of TI, TMS, tDCS, and tFUS/TUS/LIFU, this study identified distinct modality-specific patterns of literature accumulation, geographic participation, thematic organization, and reference-based knowledge structure. Within the common 2017–2026 observation window, TMS and tDCS had the largest and most diversified bibliometric literatures, strict-core tFUS/TUS/LIFU showed a mixed engineering–mechanistic–translational profile, and TI showed the fastest relative growth from a small baseline together with the smallest absolute increase and a concentrated contemporary technical knowledge base. Contemporary size- and year-matched resampling confirmed that TI's thematic concentration was not explained by present-window corpus size alone. However, the field-age-aligned analysis did not support a monotonic relationship between field age and thematic concentration: early TMS and tDCS were more thematically concentrated than TI over its first 9 years, whereas early tFUS was less concentrated. This non-monotonic ordering argues against interpreting thematic concentration as a simple marker of bibliometric maturity. Accordingly, the historical development of TMS and tDCS should not be assumed to provide a default roadmap for the trajectory of TI. The findings position TI as a potentially complementary deep-targeting approach and highlight priorities in terminology and parameter standardization, dose modeling, safety evaluation, multicenter validation, and controlled patient-oriented translational research. Its clinical role and the extent to which it can provide reliable and selective modulation of deep brain targets remain to be established.

## Data Availability

The raw bibliographic data analyzed in this study were obtained from the Web of Science Core Collection and Scopus databases, both of which require subscription access. The primary 2017–2026 search strategies and record counts are provided in Supplementary Table S1, and the field-age-aligned historical search strategies and record-flow counts are provided in Supplementary Table S15. The derived data supporting the conclusions of this study and the R scripts used for data cleaning, analysis, and visualization are available from the corresponding author upon reasonable request.
