# Supplementary material for: Across established non-invasive neuromodulation and emerging deep-targeting strategies: a comparative bibliometric analysis of TI, TMS, tDCS, and tFUS/TUS/LIFU
Source: Front Neurosci. 2026 Oct 13;20:1922512. doi: 10.3389/fnins.2026.1922512 (PMC13710099; doi:10.3389/fnins.2026.1922512)
Supplement: Supplementary file 1 [file Table_1.docx]

**Supplementary Material**

# 1 **Supplementary Methods**

## **S1. Data sources and search strategy**

The Web of Science Core Collection (WoS) and Scopus were used as the primary bibliographic databases for constructing the main analytic dataset. PubMed was used only as an independent biomedical gap-check source and was not pooled into the main dataset. Four non-invasive brain stimulation modalities were examined: temporal interference stimulation (TI), transcranial magnetic stimulation (TMS), transcranial direct current stimulation (tDCS), and transcranial focused ultrasound and related low-intensity ultrasound stimulation (tFUS/TUS/LIFU).

For each modality, a main search strategy was constructed using standardized technical terms and commonly used modality abbreviations. For TI, the search focused on temporal interference stimulation and closely related expressions. For TMS and tDCS, the search included the full technical terms and commonly used abbreviations. For tFUS/TUS/LIFU, the search included transcranial focused ultrasound, transcranial ultrasound stimulation, low-intensity focused ultrasound, and related ultrasound neuromodulation terms. Additional neuroscience-context terms related to brain, neural systems, cortex, hippocampus, striatum, thalamus, and neuromodulation were used where appropriate to reduce non-neuroscience records. The full search strings and record counts are provided in Table S1.

The search window was restricted to publications from 2017 to 2026. The 2026 records represent partial-year data up to the search date and were therefore not used for compound annual growth rate calculation. For the Web of Science and Scopus main and sensitivity searches, document types were restricted to original research articles and reviews. PubMed was used only as an independent biomedical gap-check source and was not pooled into the main analytic dataset; therefore, the same document-type restriction was not imposed on the PubMed gap-check retrieval. A separate historical retrieval was conducted for the field-age-aligned sensitivity analysis outside the 2017–2026 primary observation window and is described in Supplementary Method S10; the complete historical search strings and record counts are provided in Supplementary Table S15.

## **S2. Expanded sensitivity searches**

To evaluate whether the main search strategies missed relevant records, expanded sensitivity searches were constructed for each modality. These expanded strategies included additional abbreviations and variant terms, such as tTIS and TIS for TI; theta-burst-related terms for TMS; high-definition tDCS variants for tDCS; and tFUS, TUS, LIFU, LIFUS, and LITUS for tFUS/TUS/LIFU.

For TMS, tDCS, and tFUS/TUS/LIFU, the expanded searches did not alter the primary contemporary corpora. During revision, sensitivity-only TMS and tFUS/TUS/LIFU records identified by the original rule-based screening were additionally subjected to cross-database deduplication and record-level adjudication to construct expanded comparator corpora for a separate sensitivity analysis (Supplementary Method S14; Table S19). No additional tDCS records were identified. For TI, however, the expanded searches recovered additional candidate records containing broader abbreviations and variant terminology. These records underwent record-level manual adjudication, and substantively TI-relevant records not already present in the main corpus were incorporated after DOI- and normalized-title-level deduplication. A separate targeted re-audit was also performed for records containing prominent tACS-related terminology because terminology overlap with alternating-current stimulation could have affected the original other-modality screening rule. The comparison between the main and expanded searches and the corresponding adjudication results is summarized in Table S2. Of the 12 records excluded during the original TI relevance screening, six contained prominent tACS-related terminology and were specifically re-audited. Five of these six records were judged substantively TI-relevant and were restored to the final TI corpus, whereas one remained excluded.

## **S3. Data integration, deduplication, and relevance screening**

WoS records were exported in plain-text format, and Scopus records were exported in CSV format. Records were imported into R and standardized using the bibliometrix package. Cross-database deduplication was performed primarily by DOI matching and secondarily by title matching. After deduplication, relevance screening was conducted to reduce cross-modality contamination and marginal records.

For the primary contemporary dataset, records were initially retained when the target modality term appeared in the title or author-keyword field. This common screening yielded a pre-revision corpus of 32,420 records: 152 TI, 20,297 TMS, 10,871 tDCS, and 1,100 tFUS/TUS/LIFU. During revision, targeted corpus quality-control procedures were applied to the modality-specific datasets. Additional substantively TI-relevant records recovered through expanded-search adjudication and targeted re-audit were incorporated after record-level verification and deduplication, yielding a final TI corpus of 162 records. Targeted false-positive terminology and duplicate/republication audits reduced the TMS and tDCS corpora to 20,179 and 10,576 records, respectively. The broad tFUS/TUS/LIFU corpus remained unchanged at 1,100 records. The resulting final broad contemporary corpus therefore contained 32,017 records. After strict ultrasound-neuromodulation screening, 843 tFUS/TUS/LIFU records were retained for modality-specific comparisons, yielding a strict comparative dataset of 31,760 records.

The title/author-keyword relevance rule described above applied to the primary 2017–2026 contemporary workflow. For the title-only inclusion sensitivity analysis, author-keyword occurrence was not permitted to satisfy the inclusion criterion. For the field-age-aligned historical analysis, final inclusion was based on complete record-level relevance assessment under Codebook v2.0 rather than on title-term presence, because standardized title terms were themselves used as thematic outcomes.

## **S4. Keyword standardization and exclusion rules**

Author keywords were standardized before keyword co-occurrence and annual keyword heatmap analyses. Standardization included case normalization, harmonization of abbreviations and full technical terms, spelling standardization, and merging of synonymous expressions where appropriate. For example, variant expressions of blood–brain barrier and focused ultrasound were standardized to improve consistency across records.

For author-keyword co-occurrence networks, generic document-type, methodological, and broad population terms were removed using shared prespecified exclusion lists. The principal modality self-referential term was additionally removed for TI, TMS, and tDCS to emphasize disease applications, mechanisms, anatomical targets, stimulation parameters, and translational themes. The corresponding ultrasound-neuromodulation self-term was retained in the strict-core tFUS/TUS/LIFU network because it remained analytically informative. For annual keyword heatmaps, modality-related terminology was retained where appropriate because the purpose of the heatmaps was to display the co-evolution of terminology use and research themes over time.

## **S5. Reference co-citation network construction**

Reference co-citation analysis was conducted exclusively using Scopus cited-reference data. The final modality-specific contemporary corpora were linked to their corresponding Scopus records to construct the co-citation analysis subsets. This yielded 150 Scopus-linked TI records, 17,352 TMS records, 10,253 tDCS records, and 784 strict-core tFUS/TUS/LIFU records. Among these, 148 TI records, 17,165 TMS records, 10,153 tDCS records, and 779 tFUS/TUS/LIFU records contained structured cited-reference data, corresponding to cited-reference completeness of 98.7%, 98.9%, 99.0%, and 99.4%, respectively. For tFUS/TUS/LIFU, linkage to the broad-scope Scopus dataset followed the hierarchical EID/UT, DOI, and restricted one-to-one normalized title-and-publication-year matching procedure described in the main Methods.

Raw cited-reference fields from Web of Science and Scopus were not combined because differences in author abbreviations, journal titles, page formatting, and other bibliographic components could fragment a single cited document into multiple nodes. Cited-reference strings were therefore canonicalized before network construction. Each reference was parsed into a normalized first-author field, publication year, and normalized title information extracted from the cited-reference string. Strings sharing these fields were merged, and the most complete raw reference string was retained as the display label. References whose title information could not be reliably extracted were retained using the normalized complete raw reference string rather than being automatically merged.

Canonicalization reduced the number of distinct cited-reference strings by 22.5% for TI, 41.4% for TMS, 37.7% for tDCS, and 31.8% for strict-core tFUS/TUS/LIFU. A binary source-document-by-cited-reference matrix was constructed for each modality, and its cross-product was used as the co-citation matrix. Diagonal values represented cited-reference occurrence, whereas off-diagonal values represented pairwise co-citation frequency.

Networks were constructed at lower, main, and higher minimum cited-reference occurrence thresholds. The thresholds were 3, 5, and 8 for TI; 80, 120, and 200 for TMS; 80, 120, and 180 for tDCS; and 11, 22, and 44 for strict-core tFUS/TUS/LIFU. These modality-specific thresholds were used as operational cutoffs to obtain interpretable networks despite the marked differences in corpus size and cited-reference frequency across modalities. They were not treated as directly comparable numerical parameters across modalities; robustness was therefore evaluated using the lower and higher threshold settings. Isolated nodes were removed after thresholding. Louvain community detection was evaluated across 100 random vertex-permutation restarts. Community memberships were mapped back to the original graph, and mapping accuracy was verified by confirming identical modularity before and after reverse mapping. The partition with the highest modularity was retained. When multiple partitions shared the same maximum modularity, the solution corresponding to the most frequent community count among the tied maximum-modularity partitions was selected. Main-threshold network layouts were rendered in VOSviewer, and community colors were imported from the selected igraph Louvain partitions.

## **S6. Co-citation threshold robustness analysis**

To assess whether the reference co-citation structures depended on a single threshold choice, each modality-specific network was reconstructed using lower, main, and higher minimum cited-reference occurrence thresholds. The thresholds were 3, 5, and 8 for TI; 80, 120, and 200 for TMS; 80, 120, and 180 for tDCS; and 11, 22, and 44 for the strict-core tFUS/TUS/LIFU dataset. This analysis was intended to evaluate threshold dependence within each modality rather than to compare the absolute threshold values across modalities. References were ranked by total co-citation link strength, defined as the sum of weighted pairwise co-citation links incident on each node. Of the 20 references with the highest total co-citation link strength under the main setting, 19 and 20 remained within the corresponding Top-20 rankings under the lower and higher settings for TI; the corresponding retention counts were 19 and 19 for TMS, 20 and 19 for tDCS, and 19 and 20 for strict-core tFUS/TUS/LIFU. Community counts varied by no more than one across the three threshold settings within each modality. Detailed node, edge, modularity, and community results are reported in Table S5.

## **S7. PubMed gap-checking**

PubMed was used as an independent biomedical gap-check source and was not pooled into the WoS–Scopus analytic corpus. The PubMed records retrieved at the prespecified search date were retained unchanged during revision. After completion of the final revised corpus quality-control procedures, the same PubMed relevance definitions were reapplied and relevant PubMed records were re-matched against the final broad WoS–Scopus modality corpora. Matching used normalized DOI exact matching, with normalized-title exact matching as a fallback. The PubMed relevance pools were identical to those used in the original gap-check analysis.

PubMed-only records were classified descriptively according to major sources of non-overlap, including recent publication year, non-English records, errata or comment-type records, and other edge cases related to broader PubMed indexing, terminology, or scope. These categories were not necessarily mutually exclusive and therefore could sum to more than the number of PubMed-only records. PubMed records were used only for omission assessment and were not incorporated into the analytic corpus. The final gap-check results are summarized in Table S6.

## **S8. Software environment**

The primary contemporary bibliometric analyses were performed in R version 4.5.2. The primary contemporary workflow used bibliometrix version 5.2.1, and RStudio 2026.05.1 Build 225 was used as the integrated development environment. Network visualization was performed using VOSviewer version 1.6.20, and graph-based network statistics and Louvain community detection used igraph version 2.2.2.

The added field-age-aligned historical workflow was performed in R version 4.5.2 under Windows 11. Historical bibliographic import and record processing used bibliometrix version 5.4.1 and stringdist version 0.9.17. Title-term preprocessing and standardization used dplyr 1.2.1, tidyr 1.3.2, stringr 1.6.0, tidytext 0.4.3, and SnowballC 0.7.1. Resampling and data manipulation additionally used purrr 1.2.2 and readr 2.2.0. Supplementary workbook outputs were generated using openxlsx 4.2.8.1.

## **S9. Manual relevance audit and inter-reviewer agreement**

To assess residual scope heterogeneity in the contemporary common-screening corpora available before the revision-specific corpus quality-control updates, a publication-year-stratified manual relevance audit was conducted. The audited source populations comprised 152 TI records, 20,297 TMS records, and 10,871 tDCS records. These populations correspond to the pre-revision common-screening corpora and were retained as the sampling frames for this completed audit; the audit was not retrospectively reweighted to the subsequently revised corpora. Proportional allocation across publication-year strata produced audit samples of 100 TI records, 200 TMS records, and 200 tDCS records. Sampling was conducted without replacement within each modality-year stratum. Records added to or removed from the modality-specific corpora during revision underwent targeted record-level verification, but the original 500-record stratified audit was not repeated after these corpus corrections.

Records were classified as modality-core or non-core using Codebook v2.0. Modality-core records included studies in which the target modality was used as an intervention, comparator, substantive multimodal component, measurement or analytical method, or explicit object of mechanistic, methodological, safety, modelling, protocol, guideline, or evidence-synthesis research. Non-core records included incidental mentions, other stimulation modalities, non-neuromodulation applications, abbreviation or terminology mismatches, and records outside the predefined modality scope. Records considered non-core or ambiguous were verified against abstracts, full texts, or publisher records.

For each modality, the design-weighted core proportion was calculated across publication-year strata as

$$\hat{p_{w}}=\sum_{h} W_{h}\hat{p_{h}}$$

where $W_{h}=N_{h}/N$, Nh was the number of records in publication-year stratum h, N was the modality-specific population size, and $\hat{p_{h}}=x_{h}/n_{h}$ was the observed core proportion within that stratum. The variance was estimated as

$$Var\left( \hat{p_{w}} \right)=\sum_{h} W_{h}^{2}\left( 1-\frac{n_{h}}{N_{h}} \right)\frac{\hat{p_{h}}\left( 1-\hat{p_{h}} \right)}{n_{h}-1}$$

For strata with nh=1, the variance contribution was set to zero according to the prespecified analysis rule. A sensitivity analysis assigning the maximum binary variance of 0.25 to the singleton TI 2019 stratum changed the weighted 95% confidence interval only from approximately 76.8%–85.6% to 76.5%–85.9%, indicating that the conclusion was insensitive to this assumption. Unweighted proportions were accompanied by Wilson 95% confidence intervals.

A stratified subset of 90 records, comprising 30 records from each modality, was independently classified by GW and SW using the final Codebook v2.0. Cohen’s κ was calculated using the reviewers’ original independent classifications before consensus. Three disagreements were resolved subsequently, but consensus classifications were not used to recalculate κ.

## **S10. Field-age-aligned historical sensitivity analysis**

To evaluate potential confounding by unequal field age, separate Web of Science and Scopus searches were conducted for nine consecutive calendar-year windows anchored to the modern introduction year of TMS (1985–1993), tDCS (2000–2008), tFUS (2010–2018), and TI (2017–2025). Nine years were used because TI contributed nine complete publication years from 2017 through 2025. Applying the same window length to the other modalities aligned field age while excluding the incomplete 2026 publication year. The temporal anchors were Barker et al. (1985), Nitsche and Paulus (2000), Tufail et al. (2010), and Grossman et al. (2017), respectively. These windows were anchored to field-defining modern introductions rather than to the first record meeting the Article/Review eligibility criteria. The Barker et al. (1985) report, classified as a Letter, therefore defined the TMS start year but was not included in the Article/Review analytic corpus. Historically expanded modality nomenclature and brain/neural context terms were used to reduce terminology-era omission. The historical Web of Science and Scopus searches were conducted on August 17, 2026, with document types restricted to Articles and Reviews. The complete historical search strings and modality-specific record-flow counts are provided in Supplementary Table S15.

After initial cross-database deduplication and candidate preparation, the pre-final-review historical set comprised 1,245 records: 123 TI, 549 TMS, 179 tDCS, and 394 tFUS records. Automated lexical rules were used for triage only. Because standardized title terms were the thematic outcome of this analysis, final inclusion was deliberately made independent of title-term presence. All historical candidate records underwent record-level relevance assessment under Codebook v2.0 using titles, author/index keywords, abstracts, and full text or publisher records where necessary. Additional duplicate or metadata-duplicate records identified during final review were removed. The resulting clean field-age corpora comprised 99 TI, 439 TMS, 158 tDCS, and 76 strict-core tFUS records.

English titles were used as the primary thematic text because title coverage was complete across the historical datasets, whereas author-keyword availability differed substantially across publication eras and was particularly limited in the early TMS literature. Trailing translated or bilingual title duplicates were removed before tokenization. Standard English stopwords, modality self-identifiers, and non-thematic reporting, design, and generic population terms were removed using one prespecified cross-modality dictionary applied identically to all modalities. Inflectional variants were normalized using English Snowball stemming, supplemented by two high-confidence concept merges (cortical→cortex and neuroplasticity→plasticity). Each standardized term contributed at most once per article, yielding a document–term incidence representation for thematic analysis.

Shannon diversity was calculated as H = −Σpᵢln(pᵢ), where pᵢ represents the proportion of all standardized document–term incidences contributed by term i. Top-10 concentration (C10) was calculated as the sum of document frequencies for the ten most frequent standardized terms divided by the total number of standardized document–term incidences. Because the four historical corpora differed in size, an equal-N sensitivity analysis was performed using 10,000 simple random subsamples without replacement at n = 76, corresponding to the smallest corpus. A fixed random seed was used for reproducibility (set.seed(20260818)). TMS, tDCS, and TI were independently resampled to n = 76; the strict-core tFUS corpus already contained 76 records and therefore served as the fixed reference. Medians and 2.5th–97.5th percentile resampling ranges are reported in Figure S5 and Table S14. These ranges are descriptive resampling intervals and were not treated as formal confidence intervals or hypothesis-test results.

## **S11. Title-only inclusion sensitivity analysis**

Because author keywords contributed both to the primary contemporary relevance-screening rule and to the thematic diversity outcomes, a title-only inclusion sensitivity analysis was conducted to assess potential circularity. Starting from the final contemporary modality corpora, records were retained only when the target modality was identifiable from the title; author-keyword occurrence alone was not sufficient for inclusion. The resulting title-only corpora comprised 121 TI, 7,835 TMS, 5,050 tDCS, and 473 strict-core tFUS/TUS/LIFU records.

The title-only TI corpus was used as the matching reference. Its publication-year distribution was 1, 0, 2, 4, 7, 12, 7, 15, 41, and 32 records from 2017 through 2026, respectively. For each comparison modality, 121 records were sampled without replacement within publication-year strata to reproduce this distribution. The procedure was repeated independently 10,000 times using the same matched-resampling framework as the primary analysis. Shannon diversity and Top-10 concentration were recalculated from author keywords. TI was tested directionally for lower Shannon diversity and higher Top-10 concentration. The six thematic comparisons formed part of the full 18-test title-only matched-resampling family, and Benjamini–Hochberg-adjusted P values from that full family are reported in Table S16.

## **S12. Contemporary title-term sensitivity analysis**

To align the contemporary thematic outcomes with the field-age-aligned historical analysis, Shannon diversity and Top-10 concentration were additionally recalculated from publication titles rather than author keywords. The same prespecified historical title-term preprocessing framework described in Supplementary Method S10 was applied without modification to the final contemporary corpora. English titles were standardized using the same stopword exclusions, modality self-identifier exclusions, non-thematic term dictionary, stemming procedure, high-confidence concept merges, and document–term incidence counting rule used for the historical analysis.

Full-corpus title-term metrics were calculated for the 162 TI, 20,179 TMS, 10,576 tDCS, and 843 strict-core tFUS/TUS/LIFU records. To control for corpus size and publication-year composition, TMS, tDCS, and tFUS/TUS/LIFU were additionally resampled to reproduce the publication-year distribution of the 162-record TI corpus. Sampling was performed without replacement over 10,000 iterations. TI was tested directionally for lower title-term Shannon diversity and higher Top-10 concentration, and the six resulting P values were adjusted together using the Benjamini–Hochberg procedure.

## **S13. Broader historical tFUS screening sensitivity analysis**

Because the field-age-aligned historical tFUS corpus underwent substantially stricter relevance screening than the corresponding TMS and tDCS historical corpora, an additional sensitivity analysis was performed using a broader historical ultrasound-neuromodulation definition. The original strict-core historical tFUS corpus contained 76 records. Forty-seven historical core-adjacent records identified during the field-age screening were added, yielding a broader historical tFUS corpus of 123 records. These historical core-adjacent records are distinct from the 39 core-adjacent records identified in the 2017–2026 contemporary broad tFUS/TUS/LIFU corpus reported in Table S9.

The same prespecified title-term preprocessing and document–term incidence framework described in Supplementary Method S10 was applied to all four corpora. Because the smallest corpus under the broader definition was now TI with 99 records, the equal-size sensitivity analysis used n = 99 rather than n = 76. TMS, tDCS, and broader tFUS were independently sampled without replacement to n = 99 over 10,000 iterations; the TI corpus contained exactly 99 records and therefore served as the fixed reference. Medians and 2.5th–97.5th percentile resampling ranges were treated as descriptive robustness summaries rather than formal confidence intervals or hypothesis tests.

This broader-screening analysis was intended to assess whether the qualitative historical thematic pattern depended on the stricter tFUS screening definition. It does not eliminate differences in screening intensity among modalities and should therefore be interpreted as a robustness analysis rather than as complete harmonization of historical eligibility criteria.

## **S14. Expanded comparator sensitivity analysis**

To assess whether records retrieved only by the broader comparator search strategies affected the matched thematic findings, sensitivity-only TMS and tFUS/TUS/LIFU records flagged by the original rule-based title/keyword screening underwent additional record-level review. After cross-database deduplication, overlap checks, and restriction to records not already represented in the primary corpora, 337 unique TMS records and 36 unique tFUS/TUS/LIFU records required adjudication. Of these, 177 TMS records were judged substantively TMS-relevant, while 20 tFUS/TUS/LIFU records were judged relevant to the broader ultrasound sensitivity-search scope.

The 20 additional tFUS/TUS/LIFU records were then classified using the same strict-core boundary as the primary contemporary analysis. Seven met the strict-core definition, 12 were classified as core-adjacent, and one as non-core. The resulting expanded comparator corpora contained 20,356 TMS records, 10,576 tDCS records, and 850 strict-core tFUS/TUS/LIFU records. The primary corpora were not replaced or modified.

The size- and year-matched analysis was repeated using the same 162-record TI corpus, exact 2017–2026 publication-year matching, 10,000 iterations, the same fixed seed value, author-keyword preprocessing, original synonym mapping, empirical test directions, and 18-test Benjamini–Hochberg correction family as in the primary analysis. The expanded-comparator analysis was executed as a new full simulation rather than by reusing the primary resampling draws. Because the comparator corpora entering the simulation were modified, the random-number state entering subsequent modality-specific resampling steps was not identical to that of the primary run; consequently, the resampling summaries for an unchanged comparator corpus such as tDCS were not expected to reproduce the primary values exactly. Results are reported in Table S19.

## **S15. Two-sided empirical-P sensitivity analysis**

Because the primary thematic hypotheses used prespecified directional empirical tests, we additionally evaluated whether the results were sensitive to test direction. The saved 10,000-iteration resampling distributions were reused; no additional resampling was performed. To preserve the multiplicity structure of the primary analysis, two-sided empirical P values were recalculated for all 18 matched-resampling comparisons within each analysis set. For each comparison, the two-sided empirical P value was calculated as P = min[1, 2 × (min(b_lower, b_upper) + 1)/(B + 1)], where B = 10,000, b_lower was the number of resampled values less than or equal to the observed TI value, and b_upper was the number greater than or equal to the observed value.

The procedure was applied separately to the primary analysis and the expanded-comparator sensitivity analysis. Benjamini–Hochberg correction was applied across the complete 18-test family within each analysis set. Table S20 reports the six thematic contrasts of interest.

# 2 **Supplementary Figures**

**Figure S1.** Author keyword co-occurrence networks for (A) TI, (B) TMS, (C) tDCS, and (D) tFUS/TUS/LIFU. Networks were reconstructed from the final contemporary corpora comprising 162 TI, 20,179 TMS, 10,576 tDCS, and 843 strict-core tFUS/TUS/LIFU records. Networks were generated after the prespecified keyword-standardization and exclusion procedures described in Supplementary Method S4 and Table S8. Node size represents keyword frequency, links represent keyword co-occurrence relationships, and colors denote network clusters.

**Figure S2.** Top contributing institutions across the final contemporary modality corpora. Panels show the ten institutions with the highest publication counts for (A) TI, (B) TMS, (C) tDCS, and (D) strict-core tFUS/TUS/LIFU. Institutional information was derived from bibliographic affiliation metadata using the same extraction and full-counting procedure across modalities. Counts represent indexed publication occurrences associated with each institution. Horizontal-axis scales are panel-specific to facilitate visualization across modalities with markedly different publication volumes.

**Figure S3.** Top author-name strings across the final contemporary modality corpora. Panels show the ten most frequently occurring author-name strings for (A) TI, (B) TMS, (C) tDCS, and (D) strict-core tFUS/TUS/LIFU. Author names were parsed from the bibliographic author field using the same counting procedure across modalities. Counts are descriptive publication-frequency counts and do not represent disambiguated individual-author identities. Horizontal-axis scales are panel-specific to facilitate visualization across modalities with markedly different publication volumes.

**Figure S4.** Representative highly cited core documents by modality. The figure visualizes the five core documents per modality listed in main-text Table 2. Documents were selected from the Web of Science-indexed subset of the final contemporary corpora and ranked using Web of Science Core Collection citation counts only; Web of Science and Scopus citation counts were not combined or summed. For tFUS/TUS/LIFU, documents were selected from the strict ultrasound-neuromodulation core set (n = 843). Additional highly cited core and core-adjacent documents are reported in Supplementary Table S7. Horizontal-axis scales are panel-specific to facilitate visualization across modalities with markedly different citation counts.

**Figure S5.** Field-age-aligned title-term thematic diversity and concentration after equal-size rarefaction. Historical corpora comprised 99 TI, 439 TMS, 158 tDCS, and 76 strict-core tFUS/TUS/LIFU records. To control for corpus-size differences, TI, TMS, and tDCS were independently rarefied to n = 76 over 10,000 iterations, whereas the strict-core tFUS/TUS/LIFU corpus already contained 76 records and therefore remained fixed. Points show the median resampled values for (A) title-term Shannon diversity and (B) Top-10 term concentration; error bars show the 2.5th–97.5th percentile ranges. The fixed tFUS/TUS/LIFU value is shown without a resampling interval. These ranges are descriptive resampling summaries rather than formal confidence intervals.

# 4 **Supplementary Tables**

## **Table S1. Search strategies and record counts.**

| Database | Modality | Strategy | Records | Search string |
| --- | --- | --- | --- | --- |
| Web of Science | TI | Main | 137 | TS= ("temporal interference stimulation" OR "temporally interfering electric fields" OR "transcranial temporal interference stimulation" OR "transcranial temporal interference electrical stimulation") AND TS= (transcranial OR neuromodulat* OR brain OR neural OR neuron* OR hippocamp* OR striat* OR cortex OR cortical) AND PY= (2017-2026) |
| Web of Science | TI | Sensitivity | 365 | TS=("temporal interference stimulation" OR "temporally interfering electric fields" OR "transcranial temporal interference stimulation" OR "transcranial temporal interference electrical stimulation" OR "tTIS" OR "TIS") AND TS=(transcranial OR neuromodulat* OR brain OR neural OR neuron* OR hippocamp* OR striat* OR cortex OR cortical) AND PY=(2017-2026) |
| Web of Science | TMS | Main | 15120 | TS= ("transcranial magnetic stimulation" OR "repetitive transcranial magnetic stimulation" OR "rTMS" OR "TMS") AND TS= (brain OR neural OR neuron* OR cortex OR cortical OR neuromodulat* OR stimulation) AND PY= (2017-2026) |
| Web of Science | TMS | Sensitivity | 15550 | TS=("transcranial magnetic stimulation" OR "repetitive transcranial magnetic stimulation" OR "rTMS" OR "TMS" OR "theta burst stimulation" OR "intermittent theta burst stimulation" OR "continuous theta burst stimulation" OR "iTBS" OR "cTBS") AND TS=(brain OR neural OR neuron* OR cortex OR cortical OR neuromodulat* OR stimulation) AND PY=(2017-2026) |
| Web of Science | tDCS | Main | 7030 | TS= ("transcranial direct current stimulation" OR "transcranial direct-current stimulation" OR "tDCS") AND PY= (2017-2026) |
| Web of Science | tDCS | Sensitivity | 7030 | TS=("transcranial direct current stimulation" OR "transcranial direct-current stimulation" OR "tDCS" OR "high-definition transcranial direct current stimulation" OR "HD-tDCS" OR "HD tDCS") AND PY=(2017-2026) |
| Web of Science | tFUS/TUS/LIFU | Main | 932 | TS= ("transcranial focused ultrasound" OR "transcranial focused ultrasound stimulation" OR "transcranial ultrasound stimulation" OR "low-intensity focused ultrasound" OR "low intensity focused ultrasound" OR "low-intensity transcranial ultrasound stimulation" OR "low intensity transcranial ultrasound stimulation" OR "focused ultrasound neuromodulation" OR "ultrasound neuromodulation") AND TS= (brain OR neural OR neuron* OR neuromodulat* OR cortex OR cortical OR thalam* OR hippocamp* OR striat* OR "central nervous system") AND PY=(2017-2026) |
| Web of Science | tFUS/TUS/LIFU | Sensitivity | 1027 | TS=("transcranial focused ultrasound" OR "transcranial focused ultrasound stimulation" OR "transcranial ultrasound stimulation" OR "low-intensity focused ultrasound" OR "low intensity focused ultrasound" OR "low-intensity transcranial ultrasound stimulation" OR "low intensity transcranial ultrasound stimulation" OR "focused ultrasound neuromodulation" OR "ultrasound neuromodulation" OR "tFUS" OR "TUS" OR "LIFU" OR "LIFUS" OR "LITUS") AND TS=(brain OR neural OR neuron* OR neuromodulat* OR cortex OR cortical OR thalam* OR hippocamp* OR striat* OR "central nervous system") AND PY=(2017-2026) |
| Scopus | TI | Main | 157 | TITLE-ABS-KEY ("temporal interference stimulation" OR "temporally interfering electric fields" OR "transcranial temporal interference stimulation" OR "transcranial temporal interference electrical stimulation") AND TITLE-ABS-KEY (transcranial OR neuromodulat* OR brain OR neural OR neuron* OR hippocamp* OR striat* OR cortex OR cortical) AND PUBYEAR > 2016 AND PUBYEAR < 2027 |
| Scopus | TI | Sensitivity | 463 | TITLE-ABS-KEY("temporal interference stimulation" OR "temporally interfering electric fields" OR "transcranial temporal interference stimulation" OR "transcranial temporal interference electrical stimulation" OR "tTIS" OR "TIS") AND TITLE-ABS-KEY(transcranial OR neuromodulat* OR brain OR neural OR neuron* OR hippocamp* OR striat* OR cortex OR cortical) AND PUBYEAR > 2016 AND PUBYEAR < 2027 |
| Scopus | TMS | Main | 17704 | TITLE-ABS-KEY ("transcranial magnetic stimulation" OR "repetitive transcranial magnetic stimulation" OR "rTMS" OR "TMS") AND TITLE-ABS-KEY (brain OR neural OR neuron* OR cortex OR cortical OR neuromodulat* OR stimulation) AND PUBYEAR > 2016 AND PUBYEAR < 2027 |
| Scopus | TMS | Sensitivity | 18059 | TITLE-ABS-KEY("transcranial magnetic stimulation" OR "repetitive transcranial magnetic stimulation" OR "rTMS" OR "TMS" OR "theta burst stimulation" OR "intermittent theta burst stimulation" OR "continuous theta burst stimulation" OR "iTBS" OR "cTBS") AND TITLE-ABS-KEY(brain OR neural OR neuron* OR cortex OR cortical OR neuromodulat* OR stimulation) AND PUBYEAR > 2016 AND PUBYEAR < 2027 |
| Scopus | tDCS | Main | 10895 | TITLE-ABS-KEY ("transcranial direct current stimulation" OR "transcranial direct-current stimulation" OR "tDCS") AND PUBYEAR > 2016 AND PUBYEAR < 2027 |
| Scopus | tDCS | Sensitivity | 10895 | TITLE-ABS-KEY("transcranial direct current stimulation" OR "transcranial direct-current stimulation" OR "tDCS" OR "high-definition transcranial direct current stimulation" OR "HD-tDCS" OR "HD tDCS") AND PUBYEAR > 2016 AND PUBYEAR < 2027 |
| Scopus | tFUS/TUS/LIFU | Main | 1034 | TITLE-ABS-KEY("transcranial focused ultrasound" OR "transcranial focused ultrasound stimulation" OR "transcranial ultrasound stimulation" OR "low-intensity focused ultrasound" OR "low intensity focused ultrasound" OR "low-intensity transcranial ultrasound stimulation" OR "low intensity transcranial ultrasound stimulation" OR "focused ultrasound neuromodulation" OR "ultrasound neuromodulation") AND TITLE-ABS-KEY(brain OR neural OR neuron* OR neuromodulat* OR cortex OR cortical OR thalam* OR hippocamp* OR striat* OR "central nervous system") AND PUBYEAR > 2016 AND PUBYEAR < 2027 |
| Scopus | tFUS/TUS/LIFU | Sensitivity | 1155 | TITLE-ABS-KEY("transcranial focused ultrasound" OR "transcranial focused ultrasound stimulation" OR "transcranial ultrasound stimulation" OR "low-intensity focused ultrasound" OR "low intensity focused ultrasound" OR "low-intensity transcranial ultrasound stimulation" OR "low intensity transcranial ultrasound stimulation" OR "focused ultrasound neuromodulation" OR "ultrasound neuromodulation" OR "tFUS" OR "TUS" OR "LIFU" OR "LIFUS" OR "LITUS") AND TITLE-ABS-KEY(brain OR neural OR neuron* OR neuromodulat* OR cortex OR cortical OR thalam* OR hippocamp* OR striat* OR "central nervous system") AND PUBYEAR > 2016 AND PUBYEAR < 2027 |
| PubMed | TI | Gap-check | 234 | ("temporal interference stimulation"[TIAB] OR "temporal interference"[TIAB] OR (temporally[TIAB] AND interfering[TIAB] AND ("electric field"[TIAB] OR "electric fields"[TIAB])) OR "transcranial temporal interference stimulation"[TIAB] OR "transcranial temporal interference electrical stimulation"[TIAB]) AND (stimulation[TIAB] OR neuromodulation[TIAB] OR brain[TIAB] OR neural[TIAB] OR transcranial[TIAB] OR cortex[TIAB] OR cortical[TIAB]) AND 2017/01/01:2026/12/31[PDAT] |
| PubMed | TMS | Gap-check | 13694 | ("transcranial magnetic stimulation"[TIAB] OR "repetitive transcranial magnetic stimulation"[TIAB] OR rTMS[TIAB] OR (TMS[TIAB] AND (transcranial[TIAB] OR magnetic[TIAB] OR stimulation[TIAB]))) AND (brain OR neural OR neuron OR neurons OR cortex OR cortical OR neuromodulation OR stimulation)[TIAB] AND 2017/01/01:2026/12/31[PDAT]; exported in two date blocks |
| PubMed | tDCS | Gap-check | 7709 | ("transcranial direct current stimulation"[TIAB] OR "transcranial direct-current stimulation"[TIAB] OR tDCS[TIAB]) AND 2017/01/01:2026/12/31[PDAT] |
| PubMed | tFUS/TUS/LIFU | Gap-check | 972 | ("transcranial focused ultrasound"[TIAB] OR "transcranial focused ultrasound stimulation"[TIAB] OR "transcranial ultrasound stimulation"[TIAB] OR "low-intensity focused ultrasound"[TIAB] OR "low intensity focused ultrasound"[TIAB] OR "low-intensity transcranial ultrasound stimulation"[TIAB] OR "low intensity transcranial ultrasound stimulation"[TIAB] OR "focused ultrasound neuromodulation"[TIAB] OR "ultrasound neuromodulation"[TIAB]) AND (brain[TIAB] OR neural[TIAB] OR neuron[TIAB] OR neurons[TIAB] OR neuromodulation[TIAB] OR cortex[TIAB] OR cortical[TIAB] OR thalamus[TIAB] OR thalamic[TIAB] OR hippocampus[TIAB] OR hippocampal[TIAB] OR striatum[TIAB] OR striatal[TIAB] OR "central nervous system"[TIAB]) AND 2017/01/01:2026/12/31[PDAT] |

Search strategies and record counts. Complete main and sensitivity search strings are reported for each modality in the Web of Science Core Collection and Scopus. Complete PubMed gap-check strings are also reported. For the Web of Science and Scopus main and sensitivity searches, document types were restricted to original research articles and reviews using the corresponding database filters. PubMed was used only as an independent biomedical gap-check source and was not subjected to the same document-type restriction or pooled into the main analytic dataset. The search window was 2017–2026. TIAB = Title/Abstract; PDAT = Publication Date.

## **Table S2. Comparison of main versus sensitivity search strategies.**

| Database | Modality | Main | Sensitivity | Additional | Rule-screen-positive additional | % rule-screen-positive of additional | Rule-screen-positive additional / main (%) | Note |
| --- | --- | --- | --- | --- | --- | --- | --- | --- |
| web of science | TI | 137 | 365 | 228 | 18 | 7.9 | 13.1 | Expanded strategy; relevant sensitivity-only records underwent record-level adjudication during revision |
| web of science | TMS | 15120 | 15550 | 430 | 259 | 60.2 | 1.7 | Not pooled into the primary corpus; adjudicated for the expanded-comparator sensitivity analysis |
| web of science | tDCS | 7030 | 7030 | 0 | 0 | — | 0.0 | Expanded strategy applied; no additional records beyond main (full overlap) |
| web of science | tFUS | 932 | 1027 | 95 | 39 | 41.1 | 4.2 | Not pooled into the primary corpus; adjudicated for the expanded-comparator sensitivity analysis |
| scopus | TI | 157 | 463 | 306 | 26 | 8.5 | 16.6 | Expanded strategy; relevant sensitivity-only records underwent record-level adjudication during revision |
| scopus | TMS | 17704 | 18059 | 355 | 186 | 52.4 | 1.1 | Not pooled into the primary corpus; adjudicated for the expanded-comparator sensitivity analysis |
| scopus | tDCS | 10895 | 10895 | 0 | 0 | — | 0.0 | Expanded strategy applied; no additional records beyond main (full overlap) |
| scopus | tFUS | 1034 | 1155 | 122 | 37 | 30.3 | 3.6 | Not pooled into the primary corpus; adjudicated for the expanded-comparator sensitivity analysis |

The rule-screen-positive counts shown above are database-level sensitivity-only records identified by the original title/keyword screening and should not be interpreted as final manually adjudicated relevant records. For TMS and tFUS/TUS/LIFU, records identified through the expanded searches did not alter the primary contemporary corpora. After cross-database deduplication and overlap checks, 337 unique TMS records and 36 unique tFUS/TUS/LIFU records underwent record-level adjudication for the expanded-comparator sensitivity analysis. Of these, 177 TMS records were judged substantively TMS-relevant, while 20 tFUS/TUS/LIFU records were retained as relevant to the broader ultrasound sensitivity-search scope. The 20 tFUS/TUS/LIFU records were then assessed against the same strict-core definition used in the primary analysis: seven met the strict-core definition, 12 were classified as core-adjacent, and one as non-core. Because database-specific sensitivity results overlapped, the database-level counts in this table should not be summed to infer unique additions. “Additional” denotes records uniquely retrieved by the sensitivity strategy after database-level record-identity matching and therefore does not necessarily equal the arithmetic difference between the Sensitivity and Main counts.

## **Table S3. Relevance-screening summary.**

| Modality | Before screening | Initially excluded | Other modality | Term absent | Pre-revision retained | Revision adjustment | Final corpus |
| --- | --- | --- | --- | --- | --- | --- | --- |
| TI | 164 | 12 | 12 | 0 | 152 | +10 | 162 |
| TMS | 20,504 | 207 | 186 | 21 | 20,297 | −118 | 20,179 |
| tDCS | 11,226 | 355 | 334 | 21 | 10,871 | −295 | 10,576 |
| tFUS | 1,132 | 32 | 32 | 0 | 1,100 | 0 | 1,100 |
| Total | 33,026 | 606 | 564 | 42 | 32,420 | −403 | 32,017 |

The first five data columns describe the original common relevance-screening workflow applied before the revision-specific corpus audits. This screening retained 32,420 records from 33,026 deduplicated records. During revision, targeted corpus quality-control procedures produced a net adjustment of −403 records, yielding the final broad contemporary corpus of 32,017 records. For TI, the net revision adjustment of +10 comprised five records newly incorporated through expanded-search adjudication and five records restored through the targeted tACS-related re-audit; all additions underwent record-level verification and deduplication. For TMS and tDCS, the revision adjustments reflect removal of false-positive terminology matches and duplicate or republication records. The broad tFUS/TUS/LIFU corpus remained unchanged.

## **Table S4. Availability of structured cited-reference data in the Scopus analysis sets.**

| Modality | Scopus-linked records | With structured cited references | % with references |
| --- | --- | --- | --- |
| TI | 150 | 148 | 98.7 |
| TMS | 17,352 | 17,165 | 98.9 |
| tDCS | 10,253 | 10,153 | 99.0 |
| tFUS/TUS/LIFU | 784 | 779 | 99.4 |

Values refer to the Scopus-linked subsets of the final modality-specific corpora used for reference co-citation analysis. For tFUS/TUS/LIFU, the 843-record strict ultrasound-neuromodulation core set was linked back to the broad-scope Scopus dataset using hierarchical EID/UT, DOI, and restricted one-to-one normalized title-and-publication-year matching. Of the 784 uniquely matched strict-core records, 779 (99.4%) contained structured cited-reference data.

## **Table S5. Threshold sensitivity of the reference co-citation networks.**

| Modality | Level | Minimum cited-reference occurrence | Nodes | Edges | Louvain communities | Best modularity |
| --- | --- | --- | --- | --- | --- | --- |
| TI | Lower | 3 | 441 | 29,119 | 5 | 0.1593 |
| TI | Main | 5 | 206 | 12,078 | 4 | 0.1106 |
| TI | Higher | 8 | 108 | 4,636 | 4 | 0.0903 |
| TMS | Lower | 80 | 554 | 98,506 | 4 | 0.2785 |
| TMS | Main | 120 | 253 | 26,448 | 4 | 0.2450 |
| TMS | Higher | 200 | 92 | 4,038 | 3 | 0.2125 |
| tDCS | Lower | 80 | 410 | 69,790 | 4 | 0.1464 |
| tDCS | Main | 120 | 192 | 17,578 | 4 | 0.1158 |
| tDCS | Higher | 180 | 91 | 4,074 | 4 | 0.0853 |
| tFUS/TUS/LIFU | Lower | 11 | 434 | 60,816 | 4 | 0.1294 |
| tFUS/TUS/LIFU | Main | 22 | 212 | 20,273 | 4 | 0.0974 |
| tFUS/TUS/LIFU | Higher | 44 | 83 | 3,393 | 3 | 0.0651 |

Threshold sensitivity and community-detection results for the reference co-citation networks. Reference co-citation networks were reconstructed at modality-specific lower, main, and higher minimum cited-reference occurrence thresholds after cited-reference canonicalization and removal of isolated nodes. Each main-threshold network shown in Figure 7 represents the complete thresholded network rather than a fixed-size Top-N subset. Louvain community detection was evaluated across 100 random vertex-permutation restarts. Community memberships were mapped back to the original graph, and mapping accuracy was verified by confirming identical modularity before and after reverse mapping. The partition with the highest modularity was retained. When multiple partitions shared the same maximum modularity, the solution corresponding to the most frequent community count among the tied maximum-modularity partitions was selected. The 20 references with the highest co-citation strength in each main network showed high stability across the alternative thresholds: 19/20 and 20/20 for TI, 19/20 and 19/20 for TMS, 20/20 and 19/20 for tDCS, and 19/20 and 20/20 for strict-core tFUS/TUS/LIFU in the lower- and higher-threshold networks, respectively. At the main thresholds, modularity ranged from 0.0974 to 0.2450; these partitions were therefore interpreted as coarse descriptive communities rather than sharply separated intellectual modules.

## **Table S6. PubMed gap-check results against the final broad Web of Science–Scopus corpora.**

| Modality | PubMed relevant | In main dataset | PubMed-only | PubMed-only % | Non-English | Errata/comment | Published 2024–2026 | Other |
| --- | --- | --- | --- | --- | --- | --- | --- | --- |
| TI | 165 | 107 | 58 | 35.2 | 4 | 1 | 44 | 13 |
| TMS | 10,589 | 9,594 | 995 | 9.4 | 38 | 54 | 375 | 559 |
| tDCS | 6,268 | 5,454 | 814 | 13.0 | 15 | 66 | 286 | 477 |
| tFUS/TUS/LIFU | 711 | 631 | 80 | 11.3 | 0 | 3 | 64 | 16 |

PubMed was used as a supplementary biomedical gap-check source and was not pooled into the analytic corpus. Relevant PubMed records were re-matched against the final broad WoS–Scopus corpora using normalized DOI matching with normalized-title matching as a fallback. Overall, 15,786 of 17,733 PubMed-relevant records (89.0%) were covered by the final WoS–Scopus corpora. PubMed-only records reflected a mixture of database-indexing differences, recent publication timing, non-English records, errata or comment-type records, broader query scope, terminology-driven false-positive matches, and other edge cases. The right-hand attribution categories are descriptive and not mutually exclusive, and their counts may therefore sum to more than the number of PubMed-only records. For tFUS/TUS/LIFU, this search-coverage check used the broad 1,100-record corpus rather than the 843-record strict comparative subset.

## **Table S7. Additional highly cited core and core-adjacent source documents by modality.**

| Modality | First author (year) | Journal | Title | WoS citations | Relevance | DOI |
| --- | --- | --- | --- | --- | --- | --- |
| TI | Cao J (2020) | Ieee Transactions on Biomedical Engineering | Stimulus: Noninvasive dynamic patterns of neurostimulation using spatio-temporal interference | 66 | Core | 10.1109/TBME.2019.2919912 |
| TI | Ma R (2022) | Frontiers in Neuroscience | High gamma and beta temporal interference stimulation in the human motor cortex improves motor functions | 65 | Core | 10.3389/fnins.2021.800436 |
| TI | Vassiliadis P (2024) | Nature Human Behaviour | Non-invasive stimulation of the human striatum disrupts reinforcement learning of motor skills | 64 | Core | 10.1038/s41562-024-01901-z |
| TI | Von C J (2021) | Scientific Reports | Interindividual variability of electric fields during transcranial temporal interference stimulation (ttis) | 58 | Core | 10.1038/s41598-021-99749-0 |
| TI | Vassiliadis P (2024) | Journal of Neural Engineering | Safety, tolerability and blinding efficiency of non-invasive deep transcranial temporal interference stimulation: First experience from more than 250 sessions | 44 | Core | 10.1088/1741-2552/ad2d32 |
| TI | Yang C (2025) | Movement Disorders | Transcranial temporal interference stimulation of the right globus pallidus in Parkinson's disease | 43 | Core | 10.1002/mds.29967 |
| TI | Zhang Y (2022) | Frontiers in Human Neuroscience | Temporal interference stimulation targeting right frontoparietal areas enhances working memory in healthy individuals | 41 | Core | 10.3389/fnhum.2022.918470 |
| TI | Lee S (2022) | Computers in Biology and Medicine | Multipair transcranial temporal interference stimulation for improved focalized stimulation of deep brain regions: A simulation study | 37 | Core | 10.1016/j.compbiomed.2022.105337 |
| TI | Vieira P (2024) | Nature Communications | Temporal interference stimulation disrupts spike timing in the primate brain | 36 | Core | 10.1038/s41467-024-48962-2 |
| TI | Sunshine M (2021) | Communications Biology | Restoration of breathing after opioid overdose and spinal cord injury using temporal interference stimulation | 33 | Core-adjacent | 10.1038/s42003-020-01604-x |
| TI | Zhu X (2019) | Ieee Access | Multi-point temporal interference stimulation by using each electrode to carry different frequency currents | 31 | Core | 10.1109/ACCESS.2019.2947857 |
| TI | Von C J (2022) | Cortex | Benchmarking the effects of transcranial temporal interference stimulation (ttis) in humans | 31 | Core | 10.1016/j.cortex.2022.05.017 |
| TI | Lamos M (2025) | Movement Disorders | Noninvasive temporal interference stimulation of the subthalamic nucleus in Parkinson's disease reduces beta activity | 31 | Core | 10.1002/mds.30134 |
| TI | Wang B (2022) | Journal of Neural Engineering | Responses of model cortical neurons to temporal interference stimulation and related transcranial alternating current stimulation modalities | 30 | Core | 10.1088/1741-2552/acab30 |
| TI | Karimi F (2019) | Physics in Medicine and Biology | Computational analysis of non-invasive deep brain stimulation based on interfering electric fields | 30 | Core | 10.1088/1361-6560/ab5229 |
| TI | Liu R (2024) | NeuroImage | Temporal interference stimulation targets deep primate brain | 30 | Core | 10.1016/j.neuroimage.2024.120581 |
| TI | Gomez-Tames J (2021) | Frontiers in Neuroscience | Multiscale computational model reveals nerve response in a mouse model for temporal interference brain stimulation | 27 | Core | 10.3389/fnins.2021.684465 |
| TI | Su X (2021) | Ieee Transactions on Neural Systems and Rehabilitation Engineering | Computational modeling of spatially selective retinal stimulation with temporally interfering electric fields | 24 | Core-adjacent | 10.1109/TNSRE.2021.3055203 |
| TI | Wang M (2023) | NeuroImage | Multi-objective optimization via evolutionary algorithm (movea) for high-definition transcranial electrical stimulation of the human brain | 23 | Core-adjacent | 10.1016/j.neuroimage.2023.120331 |
| TI | Budde R (2023) | Journal of Neural Engineering | Temporal interference current stimulation in peripheral nerves is not driven by envelope extraction | 22 | Core-adjacent | 10.1088/1741-2552/acc6f1 |
| TI | Qi S (2024) | Brain Stimulation | Temporally interfering electric fields brain stimulation in primary motor cortex of mice promotes motor skill through enhancing neuroplasticity | 20 | Core | 10.1016/j.brs.2024.02.014 |
| TI | Xin Z (2021) | Frontiers in Human Neuroscience | Magnetically induced temporal interference for focal and deep-brain stimulation | 20 | Core-adjacent | 10.3389/fnhum.2021.693207 |
| TI | Zheng S (2024) | Journal of Neuroengineering and Rehabilitation | Repetitive temporal interference stimulation improves jump performance but not the postural stability in young healthy males: A randomized controlled trial | 19 | Core | 10.1186/s12984-024-01336-7 |
| TI | Stoupis D (2022) | Journal of Neural Engineering | Non-invasive stimulation with temporal interference: Optimization of the electric field deep in the brain with the use of a genetic algorithm | 19 | Core | 10.1088/1741-2552/ac89b3 |
| TMS | Drysdale A (2017) | Nature Medicine | Resting-state connectivity biomarkers define neurophysiological subtypes of depression | 1631 | Core-adjacent | 10.1038/nm.4246 |
| TMS | Polania R (2018) | Nature Neuroscience | Studying and modifying brain function with non-invasive brain stimulation | 774 | Core-adjacent | 10.1038/s41593-017-0054-4 |
| TMS | Fox M (2018) | New England Journal of Medicine | Mapping symptoms to brain networks with the human connectome | 529 | Core-adjacent | 10.1056/NEJMra1706158 |
| TMS | Mcclintock S (2018) | Journal of Clinical Psychiatry | Consensus recommendations for the clinical application of repetitive transcranial magnetic stimulation (rTMS) in the treatment of depression | 485 | Core | 10.4088/JCP.16cs10905 |
| tDCS | Knotkova H (2021) | Lancet | Neuromodulation for chronic pain | 423 | Core-adjacent | 10.1016/S0140-6736(21)00794-7 |
| tDCS | Thair H (2017) | Frontiers in Neuroscience | Transcranial direct current stimulation (tDCS): A beginner's guide for design and implementation | 388 | Core | 10.3389/fnins.2017.00641 |
| tDCS | Stagg C (2018) | Journal of Ect | Physiology of transcranial direct current stimulation | 367 | Core | 10.1097/YCT.0000000000000510 |
| tDCS | Fertonani A (2017) | Neuroscientist | Transcranial electrical stimulation: What we know and do not know about mechanisms | 361 | Core | 10.1177/1073858416631966 |
| tDCS | Huang Y (2017) | Clinical Neurophysiology | Plasticity induced by non-invasive transcranial brain stimulation: A position paper | 312 | Core-adjacent | 10.1016/j.clinph.2017.09.007 |
| tDCS | Jamil A (2017) | Journal of Physiology-london | Systematic evaluation of the impact of stimulation intensity on neuroplastic after-effects induced by transcranial direct current stimulation | 311 | Core | 10.1113/JP272738 |
| tDCS | Brunoni A (2017) | New England Journal of Medicine | Trial of electrical direct-current therapy versus escitalopram for depression | 297 | Core | 10.1056/NEJMoa1612999 |
| tDCS | Kronberg G (2017) | Brain Stimulation | Direct current stimulation modulates ltp and ltd: Activity dependence and dendritic effects | 279 | Core | 10.1016/j.brs.2016.10.001 |
| tDCS | Begemann M (2020) | Psychological Medicine | Efficacy of non-invasive brain stimulation on cognitive functioning in brain disorders: A meta-analysis | 275 | Core-adjacent | 10.1017/S0033291720003670 |
| tDCS | Buch E (2017) | Clinical Neurophysiology | Effects of tDCS on motor learning and memory formation: A consensus and critical position paper | 274 | Core | 10.1016/j.clinph.2017.01.004 |
| tDCS | Chase H (2020) | Molecular Psychiatry | Transcranial direct current stimulation: A roadmap for research, from mechanism of action to clinical implementation | 225 | Core | 10.1038/s41380-019-0499-9 |
| tDCS | Ekhtiari H (2019) | Neuroscience and Biobehavioral Reviews | Transcranial electrical and magnetic stimulation (tes and TMS) for addiction medicine: A consensus paper on the present state of the science and the road ahead | 220 | Core-adjacent | 10.1016/j.neubiorev.2019.06.007 |
| tDCS | Mutz J (2018) | Neuroscience and Biobehavioral Reviews | Efficacy and acceptability of non-invasive brain stimulation for the treatment of adult unipolar and bipolar depression: A systematic review and meta-analysis of randomised sham-controlled trials | 201 | Core-adjacent | 10.1016/j.neubiorev.2018.05.015 |
| tDCS | Nielsen J (2018) | NeuroImage | Automatic skull segmentation from mr images for realistic volume conductor models of the head: Assessment of the state-of-the-art | 196 | Core-adjacent | 10.1016/j.neuroimage.2018.03.001 |
| tDCS | Nejati V (2018) | Neuroscience | Interaction of the left dorsolateral prefrontal cortex (l-dlpfc) and right orbitofrontal cortex (ofc) in hot and cold executive functions: Evidence from transcranial direct current stimulation (tDCS) | 187 | Core | 10.1016/j.neuroscience.2017.10.042 |
| tDCS | Ladenbauer J (2017) | Journal of Neuroscience | Promoting sleep oscillations and their functional coupling by transcranial stimulation enhances memory consolidation in mild cognitive impairment | 185 | Core | 10.1523/JNEUROSCI.0260-17.2017 |
| tDCS | Fonteneau C (2019) | Brain Stimulation | Sham tDCS: A hidden source of variability? reflections for further blinded, controlled trials | 179 | Core | 10.1016/j.brs.2018.12.977 |
| tFUS | Jiang X (2019) | Ieee Transactions on Biomedical Engineering | A review of low-intensity pulsed ultrasound for therapeutic applications | 308 | Core-adjacent | 10.1109/TBME.2018.2889669 |
| tFUS | Verhagen L (2019) | Elife | Offline impact of transcranial focused ultrasound on cortical activation in primates | 249 | Core | 10.7554/eLife.40541 |
| tFUS | Krishna V (2018) | Jama Neurology | A review of the current therapies, challenges, and future directions of transcranial focused ultrasound technology advances in diagnosis and treatment | 248 | Core-adjacent | 10.1001/jamaneurol.2017.3129 |
| tFUS | Fomenko A (2018) | Brain Stimulation | Low-intensity ultrasound neuromodulation: An overview of mechanisms and emerging human applications | 239 | Core | 10.1016/j.brs.2018.08.013 |
| tFUS | Tyler W (2018) | Current Opinion in Neurobiology | Ultrasonic modulation of neural circuit activity | 190 | Core | 10.1016/j.conb.2018.04.011 |
| tFUS | Rabut C (2020) | Neuron | Ultrasound technologies for imaging and modulating neural activity | 183 | Core-adjacent | 10.1016/j.neuron.2020.09.003 |
| tFUS | Yu K (2021) | Nature Communications | Intrinsic functional neuron-type selectivity of transcranial focused ultrasound neuromodulation | 176 | Core | 10.1038/s41467-021-22743-7 |
| tFUS | Sanguinetti J (2020) | Frontiers in Human Neuroscience | Transcranial focused ultrasound to the right prefrontal cortex improves mood and alters functional connectivity in humans | 156 | Core | 10.3389/fnhum.2020.00052 |
| tFUS | Sarica C (2022) | Brain Stimulation | Human studies of transcranial ultrasound neuromodulation: A systematic review of effectiveness and safety | 151 | Core | 10.1016/j.brs.2022.05.002 |
| tFUS | Darrow D (2019) | Neurotherapeutics | Focused ultrasound for neuromodulation | 150 | Core | 10.1007/s13311-018-00691-3 |
| tFUS | Pasquinelli C (2019) | Brain Stimulation | Safety of transcranial focused ultrasound stimulation: A systematic review of the state of knowledge from both human and animal studies | 147 | Core | 10.1016/j.brs.2019.07.024 |
| tFUS | Kubanek J (2018) | Neurosurgical Focus | Neuromodulation with transcranial focused ultrasound | 147 | Core | 10.3171/2017.11.FOCUS17621 |
| tFUS | Dallapiazza R (2018) | Journal of Neurosurgery | Noninvasive neuromodulation and thalamic mapping with low-intensity focused ultrasound | 143 | Core | 10.3171/2016.11.JNS16976 |
| tFUS | Fouragnan E (2019) | Nature Neuroscience | The macaque anterior cingulate cortex translates counterfactual choice value into actual behavioral change | 143 | Core | 10.1038/s41593-019-0375-6 |
| tFUS | Yaakub S (2023) | Nature Communications | Transcranial focused ultrasound-mediated neurochemical and functional connectivity changes in deep cortical regions in humans | 142 | Core | 10.1038/s41467-023-40998-0 |
| tFUS | Di B L (2019) | Frontiers in Neurology | Transcranial focused ultrasound (tFUS) and transcranial unfocused ultrasound (TUS) neuromodulation: From theoretical principles to stimulation practices | 137 | Core | 10.3389/fneur.2019.00549 |
| tFUS | Prieto M (2018) | Ultrasound in Medicine and Biology | Activation of Piezo1 but not nav1.2 channels by ultrasound at 43 mhz | 137 | Core-adjacent | 10.1016/j.ultrasmedbio.2017.12.020 |
| tFUS | Mohammadjavadi M (2019) | Brain Stimulation | Elimination of peripheral auditory pathway activation does not affect motor responses from ultrasound neuromodulation | 134 | Core | 10.1016/j.brs.2019.03.005 |
| tFUS | Zhu J (2023) | Proceedings of the National Academy of Sciences of the United States of America | The mechanosensitive ion channel Piezo1 contributes to ultrasound neuromodulation | 127 | Core | 10.1073/pnas.2300291120 |
| tFUS | Mueller J (2017) | Journal of Neural Engineering | Numerical evaluation of the skull for human neuromodulation with transcranial focused ultrasound | 125 | Core | 10.1088/1741-2552/aa843e |
| tFUS | Fomenko A (2020) | Elife | Systematic examination of low-intensity ultrasound parameters on human motor cortex excitability and behavior | 120 | Core | 10.7554/eLife.54497 |
| tFUS | Badran B (2020) | Brain Stimulation | Sonication of the anterior thalamus with MRI-guided transcranial focused ultrasound (tFUS) alters pain thresholds in healthy adults: A double-blind, sham-controlled study | 113 | Core | 10.1016/j.brs.2020.10.007 |
| tFUS | Zeng K (2022) | Annals of Neurology | Induction of human motor cortex plasticity by theta burst transcranial ultrasound stimulation | 113 | Core | 10.1002/ana.26294 |

Documents were ranked using Web of Science Core Collection citation counts only. Web of Science and Scopus citation counts were not combined or summed. This table presents additional highly cited core and core-adjacent documents not included among the five representative core documents shown in main-text Table 2. Relevance categories were assigned through manual review. For tFUS/TUS/LIFU, documents were assessed within the strict ultrasound-neuromodulation core-screening framework.

## **Table S8. Keyword standardization and exclusion rules.**

| Category | Examples | Action |
| --- | --- | --- |
| Case normalization | Transcranial Magnetic Stimulation; transcranial magnetic stimulation | Converted to a standardized lower-case form where appropriate |
| Abbreviation harmonization | rTMS; repetitive transcranial magnetic stimulation | Harmonized or merged where the abbreviation and full term referred to the same concept |
| Modality term harmonization | transcranial focused ultrasound; focused ultrasound neuromodulation; ultrasound neuromodulation | Standardized according to modality-specific terminology where appropriate |
| Spelling and punctuation standardization | blood brain barrier; blood–brain barrier; blood-brain barrier | Standardized to a consistent spelling form |
| Anatomical term harmonization | hippocampal; hippocampus; striatal; striatum | Harmonized when used as equivalent target-region descriptors |
| Generic document-type terms removed | review; systematic review; meta-analysis; article | Removed from keyword co-occurrence networks to reduce non-thematic noise |
| Generic methodological terms removed | randomized controlled trial; clinical trial; bibliometric analysis | Removed when they did not represent modality-specific research themes |
| Modality self-referential terms | temporal interference stimulation; transcranial magnetic stimulation; transcranial direct current stimulation | Removed from the TI, TMS, and tDCS co-occurrence networks; the corresponding tFUS/TUS/LIFU modality self-term was retained in the strict-core ultrasound network |
| Selected modality-related terms retained for heatmaps | temporal interference; rTMS; focused ultrasound; ultrasound stimulation | Retained in annual keyword heatmaps to show terminology evolution over time |
| Disease and application terms retained | depression; stroke; Parkinson’s disease; Alzheimer’s disease; rehabilitation | Retained as thematic indicators of clinical or translational application |
| Mechanism and parameter terms retained | plasticity; electric field; computational modeling; theta burst stimulation | Retained as indicators of mechanistic, modeling, or stimulation-parameter themes |

Keyword standardization was performed to improve consistency across records and to reduce redundancy in co-occurrence analyses. Categories were not mutually exclusive. The exclusion of terms was applied according to the purpose of each analysis: co-occurrence networks emphasized thematic structure, whereas annual heatmaps retained selected modality-related terms to show the co-evolution of terminology and research themes.

## **Table S9. Eligibility criteria and classification results for the strict ultrasound-neuromodulation core set.**

| Classification | Operational definition | Representative inclusion/exclusion criteria | Records |
| --- | --- | --- | --- |
| Strict core | Direct ultrasound neuromodulation | mechanism, targeting, safety, human/animal stimulation | 843 |
| Core-adjacent | Mixed or related ultrasound technology | mixed imaging/modulation, indirect mechanisms | 39 |
| Non-core | Non-neuromodulation | BBB, delivery, ablation, tumor, imaging-only | 218 |

The broad-scope tFUS/TUS/LIFU dataset contained 1,100 records. Records were classified into three mutually exclusive categories using a prespecified inclusion/exclusion term dictionary followed by manual verification of borderline and ambiguous records. Strict-core records directly addressed ultrasound neuromodulation, including its neural mechanisms, acoustic parameters, targeting, modeling, safety, or in-vivo and human neuromodulation applications. Core-adjacent records addressed mixed or closely related ultrasound technologies but were not primarily focused on neuromodulation; these records were excluded from the strict comparative analyses but retained in the broad-scope dataset for supplementary characterization. Non-core records primarily addressed blood–brain barrier opening, drug delivery, thermal ablation, tumor treatment, imaging-only applications, or engineering applications not directly related to neuromodulation. Classification and manual verification were performed by GW using the prespecified operational criteria. The three categories were mutually exclusive and summed to the full broad-scope dataset: 843 strict-core, 39 core-adjacent, and 218 non-core records.

## **Table S10. Size- and year-matched resampling analysis.**

Table S10A. Thematic indicators in the size- and year-matched resampling analysis

| Indicator | Comparison modality | Test direction | TI observed | Median | 2.5th percentile | 97.5th percentile | b | Raw empirical P | BH-adjusted P |
| --- | --- | --- | --- | --- | --- | --- | --- | --- | --- |
| Author-keyword Shannon diversity | TMS | One-sided, lower | 5.5137 | 5.9597 | 5.8252 | 6.0876 | 0 | 0.0001 | 0.0004 |
| Author-keyword Shannon diversity | tDCS | One-sided, lower | 5.5137 | 5.8541 | 5.7238 | 5.9827 | 0 | 0.0001 | 0.0004 |
| Author-keyword Shannon diversity | Strict-core tFUS/TUS/LIFU | One-sided, lower | 5.5137 | 5.7238 | 5.6084 | 5.8315 | 2 | 0.0003 | 0.0009 |
| Top-10 keyword concentration | TMS | One-sided, upper | 0.2727 | 0.1716 | 0.1484 | 0.1968 | 0 | 0.0001 | 0.0004 |
| Top-10 keyword concentration | tDCS | One-sided, upper | 0.2727 | 0.1933 | 0.1683 | 0.2181 | 0 | 0.0001 | 0.0004 |
| Top-10 keyword concentration | Strict-core tFUS/TUS/LIFU | One-sided, upper | 0.2727 | 0.2092 | 0.1885 | 0.2313 | 0 | 0.0001 | 0.0004 |

Table S10B. Geographic, collaboration, and record-level indicators in the size- and year-matched resampling analysis

| Indicator | Comparison modality | Test direction | TI observed | Median | 2.5th percentile | 97.5th percentile | b | Raw empirical P | BH-adjusted P |
| --- | --- | --- | --- | --- | --- | --- | --- | --- | --- |
| Unique contributing countries | TMS | One-sided, lower | 29 | 36 | 30 | 43 | 182 | 0.0183 | 0.0450 |
| Unique contributing countries | tDCS | One-sided, lower | 29 | 36 | 29 | 43 | 282 | 0.0283 | 0.0509 |
| Unique contributing countries | Strict-core tFUS/TUS/LIFU | One-sided, lower | 29 | 26 | 21 | 31 | 9137 | 0.9137 | 0.9137 |
| MCP ratio | TMS | Two-sided | 0.2564 | 0.2166 | 0.1553 | 0.2821 | 1077 | 0.2156 | 0.2985 |
| MCP ratio | tDCS | Two-sided | 0.2564 | 0.1875 | 0.1304 | 0.2484 | 127 | 0.0256 | 0.0509 |
| MCP ratio | Strict-core tFUS/TUS/LIFU | Two-sided | 0.2564 | 0.2099 | 0.1553 | 0.2654 | 452 | 0.0906 | 0.1482 |
| Authors per publication | TMS | Two-sided | 7.5556 | 6.9506 | 6.2901 | 8.0062 | 776 | 0.1554 | 0.2331 |
| Authors per publication | tDCS | Two-sided | 7.5556 | 6.5617 | 5.9321 | 7.3458 | 99 | 0.0200 | 0.0450 |
| Authors per publication | Strict-core tFUS/TUS/LIFU | Two-sided | 7.5556 | 7.2284 | 6.6974 | 7.7716 | 1210 | 0.2422 | 0.3114 |
| Author keywords per publication | TMS | Two-sided | 5.0926 | 4.8765 | 4.4937 | 5.2654 | 1394 | 0.2790 | 0.3348 |
| Author keywords per publication | tDCS | Two-sided | 5.0926 | 4.9321 | 4.5864 | 5.2778 | 1810 | 0.3622 | 0.4074 |
| Author keywords per publication | Strict-core tFUS/TUS/LIFU | Two-sided | 5.0926 | 4.9877 | 4.5556 | 5.4198 | 3219 | 0.6439 | 0.6818 |

TI comprised all 162 eligible records in the final contemporary corpus. For TMS, tDCS, and the strict-core tFUS/TUS/LIFU dataset, 162 records were sampled without replacement within publication-year strata to reproduce the year-by-year distribution of TI. The procedure was repeated independently 10,000 times using a fixed random seed (set.seed(20260724)). Values for the comparison modalities are presented as the median and 2.5th–97.5th percentile empirical interval across the resamples. Empirical P values for author-keyword Shannon diversity, Top-10 keyword concentration, and contributing countries were one-sided and directional, whereas P values for MCP ratio, authors per publication, and author keywords per publication were two-sided. Empirical P values were calculated using the add-one correction. The 18 primary P values arising from six indicators across three comparison modalities were adjusted jointly using the Benjamini–Hochberg false discovery rate procedure. A raw empirical P value of 0.0001 indicates that no resampled value was as extreme as the observed TI value in the prespecified direction after application of the add-one correction.

## **Table S11. Author-keyword availability and rule-based standardization across modalities.**

Table S11A. Author-keyword field completeness in the full modality-specific datasets

| Modality | Total records | Records with author keywords | Non-empty rate, % | Median keywords per non-empty record | Mean keywords per non-empty record |
| --- | --- | --- | --- | --- | --- |
| TI | 162 | 135 | 83.3 | 5 | 6.14 |
| TMS | 20,179 | 17,473 | 86.6 | 5 | 5.49 |
| tDCS | 10,576 | 9,333 | 88.2 | 5 | 5.40 |
| Strict-core tFUS/TUS/LIFU | 843 | 706 | 83.7 | 5 | 5.86 |

Table S11B. Author-keyword field completeness in the size- and year-matched resamples

| Modality | Median completeness, % | 2.5th percentile, % | 97.5th percentile, % |
| --- | --- | --- | --- |
| TI | 83.3 | — | — |
| TMS | 88.3 | 82.7 | 92.6 |
| tDCS | 90.1 | 85.2 | 94.4 |
| Strict-core tFUS/TUS/LIFU | 84.0 | 78.4 | 88.3 |

Table S11C. Rule-based author-keyword standardization used in the contemporary analyses

| Rule category | Final implementation | Analysis scope |
| --- | --- | --- |
| Prespecified synonym and abbreviation mapping | Author-keyword variants were standardized using a single prespecified mapping dictionary shared across modalities. | Figure 6 and Figure S1 |
| Orthographic normalization | Trivial spelling, hyphenation, punctuation, and singular/plural variants were normalized consistently. | Figure 6 and Figure S1 |
| Document-type terms | A shared exclusion list removed generic document-type terms, including review, systematic review, meta-analysis, article, original article, case report, letter, and editorial. | Primarily co-occurrence preprocessing |
| Generic methodological and population terms | A shared exclusion list removed generic terms such as randomized controlled trial, controlled trial, clinical trial, bibliometric analysis, statistical analysis, human(s), animals, adult, male, and female. | Primarily co-occurrence preprocessing |
| Modality self-referential terms | The principal modality self-term was removed for TI, TMS, and tDCS co-occurrence networks; no corresponding self-term was removed from the strict-core tFUS/TUS/LIFU network. | Figure S1 only |
| Modality terminology in annual heatmaps | Modality-related terminology was retained where needed to display temporal evolution of terminology and research themes. | Figure 6 only |

Non-empty rates in Table S11A were calculated using all records in each final modality-specific dataset as the denominator. Median and mean keyword counts were calculated only among records with non-empty author-keyword fields. Table S11B reports author-keyword field completeness within 10,000 size- and year-matched resamples using the year distribution of the final 162-record TI corpus; the TI value represents the observed completeness across all 162 TI records and is therefore not accompanied by a resampling interval. Table S11C summarizes the prespecified rule-based keyword-standardization framework used in the final contemporary analyses. A shared mapping dictionary and generic exclusion rules were applied across modalities, with analysis-specific handling of modality self-referential terms for co-occurrence networks and annual heatmaps.

## **Table S12. Sensitivity analysis after harmonization of modality-family keyword variants.**

| Indicator | Comparison modality | Test direction | TI observed | Median | 2.5th percentile | 97.5th percentile | b | Raw empirical P | BH-adjusted P |
| --- | --- | --- | --- | --- | --- | --- | --- | --- | --- |
| Author-keyword Shannon diversity | TMS | One-sided, lower | 5.2437 | 5.8063 | 5.6494 | 5.9521 | 0 | 0.0001 | 0.0001 |
| Author-keyword Shannon diversity | tDCS | One-sided, lower | 5.2437 | 5.7207 | 5.5761 | 5.8636 | 0 | 0.0001 | 0.0001 |
| Author-keyword Shannon diversity | Strict-core tFUS/TUS/LIFU | One-sided, lower | 5.2437 | 5.4353 | 5.2983 | 5.5580 | 43 | 0.0044 | 0.0044 |
| Top-10 keyword concentration | TMS | One-sided, upper | 0.3273 | 0.2024 | 0.1755 | 0.2313 | 0 | 0.0001 | 0.0001 |
| Top-10 keyword concentration | tDCS | One-sided, upper | 0.3273 | 0.2258 | 0.1971 | 0.2550 | 0 | 0.0001 | 0.0001 |
| Top-10 keyword concentration | Strict-core tFUS/TUS/LIFU | One-sided, upper | 0.3273 | 0.2751 | 0.2520 | 0.3001 | 0 | 0.0001 | 0.0001 |

Modality-family variants and established subtype labels were additionally harmonized across all four final contemporary corpora before repeating the size- and year-matched analysis. The harmonization rules were preserved from the original sensitivity analysis and included, for example, temporal interference stimulation, temporal interference, transcranial temporal interference stimulation, tTIS, TIS, TI, and related predefined variants within the TI family; transcranial magnetic stimulation, TMS, repetitive transcranial magnetic stimulation, rTMS, and theta-burst stimulation variants within the TMS family; transcranial direct current stimulation, tDCS, and high-definition tDCS variants within the tDCS family; and transcranial focused ultrasound, transcranial ultrasound stimulation, low-intensity focused ultrasound, tFUS, TUS, and LIFU variants within the ultrasound-neuromodulation family. TI comprised all 162 final TI records. For TMS, tDCS, and strict-core tFUS/TUS/LIFU, 162 records were sampled without replacement within publication-year strata to reproduce the TI year distribution. The procedure was repeated independently 10,000 times using a fixed random seed (set.seed(20260724)). Empirical P values were calculated using the add-one correction, P=(b+1)/(B+1), and the six resulting P values were adjusted jointly using the Benjamini–Hochberg false discovery rate procedure. After modality-family harmonization, TI continued to show lower author-keyword Shannon diversity and higher Top-10 keyword concentration than all three comparison modalities.

## **Table S13. Publication-year-stratified manual relevance audit and inter-reviewer agreement.**

Table S13A. Manual relevance audit: overall results

| Modality | Population N | Audited n | Core | Non-core | Unweighted core proportion, % (Wilson 95% CI) | Design-weighted core proportion, % (95% CI) |
| --- | --- | --- | --- | --- | --- | --- |
| TI | 152 | 100 | 81 | 19 | 81.0 (72.2–87.5) | 81.2 (76.8–85.6) |
| TMS | 20,297 | 200 | 146 | 54 | 73.0 (66.5–78.7) | 72.7 (66.5–78.9) |
| tDCS | 10,871 | 200 | 147 | 53 | 73.5 (67.0–79.1) | 73.4 (67.3–79.5) |
| Total, descriptive only | — | 500 | 374 | 126 | 74.8 (70.8–78.4) | — |

Table S13B. Year-stratified manual relevance audit details

| Modality | Year | Population N_h | Sample n_h | Core x_h | Non-core | Stratum core proportion p_h | Sampling fraction f_h | Stratum weight W_h | Design weight d_h | Weighted contribution | Variance contribution | Variance note |
| --- | --- | --- | --- | --- | --- | --- | --- | --- | --- | --- | --- | --- |
| TI | 2017 | 1 | 1 | 1 | 0 | 1.0000 | 1.0000 | 0.0066 | 1.0000 | 0.0066 | 0.000000 | n_h = 1; variance contribution set to zero under the prespecified analysis rule |
| TI | 2018 | 1 | 1 | 1 | 0 | 1.0000 | 1.0000 | 0.0066 | 1.0000 | 0.0066 | 0.000000 | n_h = 1; variance contribution set to zero under the prespecified analysis rule |
| TI | 2019 | 3 | 1 | 1 | 0 | 1.0000 | 0.3333 | 0.0197 | 3.0000 | 0.0197 | 0.000000 | n_h = 1; variance contribution set to zero under the prespecified analysis rule |
| TI | 2020 | 4 | 2 | 2 | 0 | 1.0000 | 0.5000 | 0.0263 | 2.0000 | 0.0263 | 0.000000 |  |
| TI | 2021 | 8 | 5 | 3 | 2 | 0.6000 | 0.6250 | 0.0526 | 1.6000 | 0.0316 | 0.000062 |  |
| TI | 2022 | 13 | 8 | 6 | 2 | 0.7500 | 0.6154 | 0.0855 | 1.6250 | 0.0641 | 0.000075 |  |
| TI | 2023 | 13 | 8 | 5 | 3 | 0.6250 | 0.6154 | 0.0855 | 1.6250 | 0.0535 | 0.000094 |  |
| TI | 2024 | 18 | 11 | 10 | 1 | 0.9091 | 0.6111 | 0.1184 | 1.6364 | 0.1077 | 0.000045 |  |
| TI | 2025 | 53 | 38 | 29 | 9 | 0.7632 | 0.7170 | 0.3487 | 1.3947 | 0.2661 | 0.000168 |  |
| TI | 2026 | 38 | 25 | 23 | 2 | 0.9200 | 0.6579 | 0.2500 | 1.5200 | 0.2300 | 0.000066 |  |
| TMS | 2017 | 1554 | 15 | 10 | 5 | 0.6667 | 0.0097 | 0.0766 | 103.6000 | 0.0510 | 0.000092 |  |
| TMS | 2018 | 1676 | 16 | 13 | 3 | 0.8125 | 0.0095 | 0.0826 | 104.7500 | 0.0671 | 0.000069 |  |
| TMS | 2019 | 1735 | 17 | 12 | 5 | 0.7059 | 0.0098 | 0.0855 | 102.0588 | 0.0603 | 0.000094 |  |
| TMS | 2020 | 1923 | 18 | 11 | 7 | 0.6111 | 0.0094 | 0.0947 | 106.8333 | 0.0579 | 0.000124 |  |
| TMS | 2021 | 2125 | 20 | 15 | 5 | 0.7500 | 0.0094 | 0.1047 | 106.2500 | 0.0785 | 0.000107 |  |
| TMS | 2022 | 2259 | 22 | 14 | 8 | 0.6364 | 0.0097 | 0.1113 | 102.6818 | 0.0708 | 0.000135 |  |
| TMS | 2023 | 2253 | 22 | 15 | 7 | 0.6818 | 0.0098 | 0.1110 | 102.4091 | 0.0757 | 0.000126 |  |
| TMS | 2024 | 2474 | 24 | 16 | 8 | 0.6667 | 0.0097 | 0.1219 | 103.0833 | 0.0813 | 0.000142 |  |
| TMS | 2025 | 2757 | 31 | 27 | 4 | 0.8710 | 0.0112 | 0.1358 | 88.9355 | 0.1183 | 0.000068 |  |
| TMS | 2026 | 1541 | 15 | 13 | 2 | 0.8667 | 0.0097 | 0.0759 | 102.7333 | 0.0658 | 0.000047 |  |
| tDCS | 2017 | 746 | 13 | 12 | 1 | 0.9231 | 0.0174 | 0.0686 | 57.3846 | 0.0633 | 0.000027 |  |
| tDCS | 2018 | 846 | 15 | 11 | 4 | 0.7333 | 0.0177 | 0.0778 | 56.4000 | 0.0571 | 0.000083 |  |
| tDCS | 2019 | 922 | 16 | 12 | 4 | 0.7500 | 0.0174 | 0.0848 | 57.6250 | 0.0636 | 0.000088 |  |
| tDCS | 2020 | 996 | 18 | 11 | 7 | 0.6111 | 0.0181 | 0.0916 | 55.3333 | 0.0560 | 0.000115 |  |
| tDCS | 2021 | 1221 | 22 | 16 | 6 | 0.7273 | 0.0180 | 0.1123 | 55.5000 | 0.0817 | 0.000117 |  |
| tDCS | 2022 | 1265 | 23 | 18 | 5 | 0.7826 | 0.0182 | 0.1164 | 55.0000 | 0.0911 | 0.000103 |  |
| tDCS | 2023 | 1197 | 22 | 14 | 8 | 0.6364 | 0.0184 | 0.1101 | 54.4091 | 0.0701 | 0.000131 |  |
| tDCS | 2024 | 1432 | 26 | 20 | 6 | 0.7692 | 0.0182 | 0.1317 | 55.0769 | 0.1013 | 0.000121 |  |
| tDCS | 2025 | 1487 | 32 | 25 | 7 | 0.7813 | 0.0215 | 0.1368 | 46.4688 | 0.1069 | 0.000101 |  |
| tDCS | 2026 | 759 | 13 | 8 | 5 | 0.6154 | 0.0171 | 0.0698 | 58.3846 | 0.0430 | 0.000095 |  |

Table S13C. Reasons for non-core classification among audited TI records

| Modality | Exclusion reason | Count | % among non-core | % among all audited records |
| --- | --- | --- | --- | --- |
| TI | Other modality | 9 | 47.4% | 9.0% |
| TI | Out-of-scope non-transcranial TI | 6 | 31.6% | 6.0% |
| TI | Incidental mention | 2 | 10.5% | 2.0% |
| TI | Non-neuromodulation / terminology mismatch | 2 | 10.5% | 2.0% |

Table S13D. Inter-reviewer agreement under Codebook v2.0

| Measure | Result |
| --- | --- |
| Independently double-coded records | 90 |
| Agreements | 87 |
| Disagreements | 3 |
| Overall agreement | 96.7% |
| Cohen’s κ | 0.902 |
| Asymptotic SE | 0.056 |
| 95% CI | 0.793–1.000 |

| GW \ SW | Core | Non-core | Total |
| --- | --- | --- | --- |
| Core | 69 | 1 | 70 |
| Non-core | 2 | 18 | 20 |
| Total | 71 | 19 | 90 |

The manual relevance audit included 100 TI, 200 TMS, and 200 tDCS records sampled proportionally across publication-year strata without replacement. Design-weighted core proportions were calculated using population year-stratum weights, and variance estimates incorporated the finite population correction. Unweighted proportions are accompanied by Wilson 95% confidence intervals. The pooled value of 374/500 is descriptive only because the modality-specific samples had different sampling fractions; no cross-modality design-weighted total was calculated. Cohen’s κ was calculated from the original independent classifications of the 90 double-coded records before consensus discussion. The population sizes reported in Tables S13A–B refer to the pre-revision common-screening corpora from which the 500-record audit was originally sampled, rather than to the subsequently revised modality corpora. The weighted estimates therefore characterize those pre-revision screening populations and were not recomputed after the revision-specific record-level corpus updates.

## **Table S14. Field-age-aligned title-term diversity and concentration metrics across TMS, tDCS, tFUS, and TI.**

| Modality | Field-age window | Final N | Full-corpus H | Full-corpus C10 | Equal-N H, median [2.5th–97.5th] | Equal-N C10, median [2.5th–97.5th] |
| --- | --- | --- | --- | --- | --- | --- |
| TMS | 1985–1993 | 439 | 5.5959 | 0.2847 | 4.9918 [4.8300–5.1404] | 0.2910 [0.2506–0.3333] |
| tDCS | 2000–2008 | 158 | 5.3164 | 0.2583 | 4.9802 [4.8376–5.1067] | 0.2668 [0.2353–0.3011] |
| tFUS | 2010–2018 | 76 | 5.2860 | 0.1636 | 5.2860 [5.2860–5.2860] | 0.1636 [0.1636–0.1636] |
| TI | 2017–2025 | 99 | 5.2716 | 0.2053 | 5.1385 [5.0532–5.2121] | 0.2100 [0.1890–0.2310] |

Full-corpus metrics are descriptive because corpus sizes differ. Equal-N estimates are based on 10,000 simple random subsamples without replacement at n = 76. Bracketed values are 2.5th–97.5th percentile resampling ranges rather than formal confidence intervals. The strict-core tFUS corpus contained exactly 76 records and was therefore retained as a fixed reference. Barker et al. (1985) defined the TMS temporal anchor but, as a Letter, was not included in the Article/Review analytic corpus.

## **Table S15. Search strategies and record flow for the field-age-aligned historical sensitivity analysis.**

Table S15A. Historical retrieval and record-flow counts

| Modality | Field-age window | WoS exported N | WoS after field-year QC | Scopus exported N | Scopus after field-year QC | Combined before cross-database deduplication | Pre-final-review candidate set | Final clean corpus |
| --- | --- | --- | --- | --- | --- | --- | --- | --- |
| TMS | 1985–1993 | 387 | 387 | 452 | 452 | 839 | 549 | 439 |
| tDCS | 2000–2008 | 164 | 164 | 168 | 168 | 332 | 179 | 158 |
| tFUS | 2010–2018 | 311 | 311 | 322 | 322 | 633 | 394 | 76 |
| TI | 2017–2025 | 111 | 108 | 116 | 116 | 224 | 123 | 99 |

The historical Web of Science Core Collection and Scopus searches were conducted on August 17, 2026, with document types restricted to Articles and Reviews. The “combined before cross-database deduplication” column represents records retained after field-year quality control and before cross-database duplicate removal. For TI, 111 Web of Science records were exported, of which three records were assigned to 2026 during metadata quality control and were therefore removed from the predefined 2017–2025 field-age window, leaving 108 Web of Science records and 224 records across the two databases before deduplication. The pre-final-review candidate set represents the record set entering complete record-level adjudication. Additional metadata duplicates identified during final review were removed before construction of the final clean corpora. The final clean tFUS corpus represents the strict-core ultrasound-neuromodulation set.

Table S15B. Complete historical search strings

| Database | Modality | Field-age window | Search string |
| --- | --- | --- | --- |
| Web of Science | TMS | 1985–1993 | TS=("transcranial magnetic stimulation" OR "repetitive transcranial magnetic stimulation" OR "magnetic stimulation" OR "non-invasive magnetic stimulation" OR "noninvasive magnetic stimulation" OR "magnetic brain stimulation" OR "magnetic cortical stimulation") AND TS=(brain OR cortex OR cortical OR "motor cortex" OR corticospinal OR neural OR neuron* OR "central nervous system") AND PY=(1985-1993) |
| Scopus | TMS | 1985–1993 | TITLE-ABS-KEY("transcranial magnetic stimulation" OR "repetitive transcranial magnetic stimulation" OR "magnetic stimulation" OR "non-invasive magnetic stimulation" OR "noninvasive magnetic stimulation" OR "magnetic brain stimulation" OR "magnetic cortical stimulation") AND TITLE-ABS-KEY(brain OR cortex OR cortical OR "motor cortex" OR corticospinal OR neural OR neuron* OR "central nervous system") AND PUBYEAR > 1984 AND PUBYEAR < 1994 |
| Web of Science | tDCS | 2000–2008 | TS=("transcranial direct current stimulation" OR "transcranial direct-current stimulation" OR "transcranial DC stimulation" OR "transcranial direct current" OR "tDCS") AND TS=(brain OR cortex OR cortical OR cerebral OR neural OR neuron* OR neuromodulat* OR excitability) AND PY=(2000-2008) |
| Scopus | tDCS | 2000–2008 | TITLE-ABS-KEY("transcranial direct current stimulation" OR "transcranial direct-current stimulation" OR "transcranial DC stimulation" OR "transcranial direct current" OR "tDCS") AND TITLE-ABS-KEY(brain OR cortex OR cortical OR cerebral OR neural OR neuron* OR neuromodulat* OR excitability) AND PUBYEAR > 1999 AND PUBYEAR < 2009 |
| Web of Science | tFUS | 2010–2018 | TS=("transcranial focused ultrasound" OR "transcranial focused ultrasound stimulation" OR "transcranial ultrasound stimulation" OR "transcranial pulsed ultrasound" OR "transcranial ultrasound" OR "low-intensity focused ultrasound" OR "low intensity focused ultrasound" OR "low-intensity transcranial ultrasound stimulation" OR "low intensity transcranial ultrasound stimulation" OR "focused ultrasound neuromodulation" OR "ultrasound neuromodulation" OR "ultrasonic neuromodulation") AND TS=(brain OR neural OR neuron* OR neuromodulat* OR cortex OR cortical OR thalam* OR hippocamp* OR striat* OR "central nervous system") AND PY=(2010-2018) |
| Scopus | tFUS | 2010–2018 | TITLE-ABS-KEY("transcranial focused ultrasound" OR "transcranial focused ultrasound stimulation" OR "transcranial ultrasound stimulation" OR "transcranial pulsed ultrasound" OR "transcranial ultrasound" OR "low-intensity focused ultrasound" OR "low intensity focused ultrasound" OR "low-intensity transcranial ultrasound stimulation" OR "low intensity transcranial ultrasound stimulation" OR "focused ultrasound neuromodulation" OR "ultrasound neuromodulation" OR "ultrasonic neuromodulation") AND TITLE-ABS-KEY(brain OR neural OR neuron* OR neuromodulat* OR cortex OR cortical OR thalam* OR hippocamp* OR striat* OR "central nervous system") AND PUBYEAR > 2009 AND PUBYEAR < 2019 |
| Web of Science | TI | 2017–2025 | TS=("temporal interference stimulation" OR "temporally interfering electric fields" OR "transcranial temporal interference stimulation" OR "transcranial temporal interference electrical stimulation") AND TS=(transcranial OR neuromodulat* OR brain OR neural OR neuron* OR hippocamp* OR striat* OR cortex OR cortical) AND PY=(2017-2025) |
| Scopus | TI | 2017–2025 | TITLE-ABS-KEY("temporal interference stimulation" OR "temporally interfering electric fields" OR "transcranial temporal interference stimulation" OR "transcranial temporal interference electrical stimulation") AND TITLE-ABS-KEY(transcranial OR neuromodulat* OR brain OR neural OR neuron* OR hippocamp* OR striat* OR cortex OR cortical) AND PUBYEAR > 2016 AND PUBYEAR < 2026 |

Searches were conducted on August 17, 2026. Document types were restricted to Articles and Reviews using the corresponding database filters. Historical queries were intentionally broader than the contemporary modality queries where necessary to capture terminology used during early field development. Automated lexical rules were used for triage only; final eligibility was determined by complete record-level review under Codebook v2.0.

## **Table S16. Title-only inclusion sensitivity analysis.**

Table S16A. Title-only corpus sizes

| Modality | Title-only corpus N |
| --- | --- |
| TI | 121 |
| TMS | 7,835 |
| tDCS | 5,050 |
| Strict-core tFUS/TUS/LIFU | 473 |

Table S16B. Author-keyword thematic indicators in the title-only size- and year-matched sensitivity analysis

| Indicator | Comparison modality | Test direction | TI observed | Median | 2.5th percentile | 97.5th percentile | b | Raw empirical P | BH-adjusted P |
| --- | --- | --- | --- | --- | --- | --- | --- | --- | --- |
| Author-keyword Shannon diversity | TMS | One-sided, lower | 5.2253 | 5.4854 | 5.3374 | 5.6242 | 2 | 0.0003 | 0.0013 |
| Author-keyword Shannon diversity | tDCS | One-sided, lower | 5.2253 | 5.3828 | 5.2528 | 5.5060 | 90 | 0.0091 | 0.0182 |
| Author-keyword Shannon diversity | Strict-core tFUS/TUS/LIFU | One-sided, lower | 5.2253 | 5.5063 | 5.3876 | 5.6145 | 0 | 0.0001 | 0.0009 |
| Top-10 keyword concentration | TMS | One-sided, upper | 0.2940 | 0.2399 | 0.2126 | 0.2697 | 1 | 0.0002 | 0.0012 |
| Top-10 keyword concentration | tDCS | One-sided, upper | 0.2940 | 0.2535 | 0.2282 | 0.2808 | 14 | 0.0015 | 0.0045 |
| Top-10 keyword concentration | Strict-core tFUS/TUS/LIFU | One-sided, upper | 0.2940 | 0.2158 | 0.1938 | 0.2394 | 0 | 0.0001 | 0.0009 |

The title-only sensitivity analysis excluded records that met the primary relevance criterion solely through author-keyword occurrence. TI comprised all 121 title-only records, and comparison modalities were sampled without replacement within publication-year strata to reproduce the TI year distribution over 10,000 iterations. The six Shannon-diversity and Top-10-concentration comparisons shown here represent the thematic contrasts of interest within the full 18-test title-only matched-resampling analysis; the reported Benjamini–Hochberg-adjusted P values therefore reflect correction across all 18 tests. All six thematic comparisons retained the same direction as the primary analysis and remained significant after multiplicity correction.

## **Table S17. Contemporary title-term sensitivity analysis using the prespecified historical preprocessing framework.**

Table S17A. Full-corpus contemporary title-term diversity and concentration metrics

| Modality | N | Shannon H | C10 | Unique terms | Total term incidences |
| --- | --- | --- | --- | --- | --- |
| TI | 162 | 5.7524 | 0.1603 | 459 | 886 |
| TMS | 20,179 | 7.0213 | 0.1250 | 8,735 | 159,169 |
| tDCS | 10,576 | 7.0100 | 0.1076 | 6,284 | 79,414 |
| Strict-core tFUS/TUS/LIFU | 843 | 6.5796 | 0.1073 | 1,454 | 4,968 |

Table S17B. Size- and year-matched contemporary title-term comparisons

| Indicator | Comparison | Direction | TI observed | Median | 2.5th | 97.5th | b | Raw P | BH-adjusted P |
| --- | --- | --- | --- | --- | --- | --- | --- | --- | --- |
| Title-term Shannon diversity | TMS | One-sided, lower | 5.7524 | 6.1267 | 6.0175 | 6.2325 | 0 | 0.0001 | 0.0001 |
| Title-term Shannon diversity | tDCS | One-sided, lower | 5.7524 | 6.1065 | 6.0017 | 6.2095 | 0 | 0.0001 | 0.0001 |
| Title-term Shannon diversity | Strict-core tFUS/TUS/LIFU | One-sided, lower | 5.7524 | 6.0378 | 5.9565 | 6.1159 | 0 | 0.0001 | 0.0001 |
| Title-term Top-10 concentration | TMS | One-sided, upper | 0.1603 | 0.1316 | 0.1138 | 0.1503 | 12 | 0.0013 | 0.0013 |
| Title-term Top-10 concentration | tDCS | One-sided, upper | 0.1603 | 0.1189 | 0.1017 | 0.1376 | 0 | 0.0001 | 0.0001 |
| Title-term Top-10 concentration | Strict-core tFUS/TUS/LIFU | One-sided, upper | 0.1603 | 0.1080 | 0.0941 | 0.1237 | 0 | 0.0001 | 0.0001 |

Contemporary title terms were processed using exactly the same prespecified dictionary, preprocessing rules, and document–term incidence definition used in the field-age-aligned historical analysis. Full-corpus values in Table S17A are descriptive because corpus sizes differ substantially. Table S17B reports 10,000 size- and publication-year-matched resamples using the final 162-record TI corpus as the reference. Empirical P values were calculated using the add-one correction, and the six title-term comparisons were adjusted jointly using the Benjamini–Hochberg procedure. TI retained lower title-term Shannon diversity and higher Top-10 concentration than all three matched comparison modalities.

## **Table S18. Broader historical tFUS screening sensitivity analysis.**

Table S18A. Full-corpus historical title-term metrics using the broader tFUS definition

| Modality | N | Shannon H | C10 | Unique terms | Total incidences |
| --- | --- | --- | --- | --- | --- |
| TMS | 439 | 5.5959 | 0.2847 | 747 | 2,624 |
| tDCS | 158 | 5.3164 | 0.2583 | 370 | 786 |
| Broader tFUS | 123 | 5.5975 | 0.1597 | 357 | 601 |
| TI | 99 | 5.2716 | 0.2053 | 273 | 487 |

Table S18B. Equal-N historical sensitivity analysis using the broader tFUS definition

| Modality | Equal N | Shannon median | Shannon 2.5th | Shannon 97.5th | C10 median | C10 2.5th | C10 97.5th |
| --- | --- | --- | --- | --- | --- | --- | --- |
| TMS | 99 | 5.1115 | 4.9678 | 5.2459 | 0.2889 | 0.2534 | 0.3249 |
| tDCS | 99 | 5.1116 | 5.0019 | 5.2120 | 0.2631 | 0.2393 | 0.2884 |
| Broader tFUS | 99 | 5.4811 | 5.4177 | 5.5383 | 0.1618 | 0.1481 | 0.1746 |
| TI | 99 | 5.2716 | 5.2716 | 5.2716 | 0.2053 | 0.2053 | 0.2053 |

The broader historical tFUS corpus combined the 76 strict-core historical records used in the primary field-age analysis with 47 historical core-adjacent records, yielding 123 records. These historical core-adjacent records are distinct from the 39 core-adjacent records identified in the 2017–2026 contemporary broad tFUS/TUS/LIFU corpus reported in Table S9. Because TI (n = 99) became the smallest corpus under this broader definition, the equal-N sensitivity analysis used n = 99. TMS, tDCS, and broader tFUS were independently resampled without replacement 10,000 times; TI served as the fixed 99-record reference. The broader tFUS corpus continued to show lower Top-10 concentration than both TMS and tDCS and higher Shannon diversity than both modalities. Thus, the qualitative non-monotonic historical pattern was preserved when the tFUS eligibility definition was broadened. Resampling ranges are descriptive and do not eliminate differences in screening intensity across historical modalities.

## **Table S19. Sensitivity analysis using expanded comparator corpora after record-level adjudication.**

| Indicator | Comparison modality | Primary corpus N | Expanded corpus N | TI observed | Matched median | 2.5th percentile | 97.5th percentile | Empirical P | BH-adjusted P | Direction preserved |
| --- | --- | --- | --- | --- | --- | --- | --- | --- | --- | --- |
| Author-keyword Shannon diversity | TMS | 20,179 | 20,356 | 5.5137 | 5.9594 | 5.8295 | 6.0900 | <0.001 | <0.001 | Yes |
| Author-keyword Shannon diversity | tDCS | 10,576 | 10,576 | 5.5137 | 5.8538 | 5.7235 | 5.9825 | <0.001 | <0.001 | Yes |
| Author-keyword Shannon diversity | Strict-core tFUS/TUS/LIFU | 843 | 850 | 5.5137 | 5.7263 | 5.6147 | 5.8344 | <0.001 | <0.001 | Yes |
| Top-10 keyword concentration | TMS | 20,179 | 20,356 | 0.2727 | 0.1711 | 0.1475 | 0.1954 | <0.001 | <0.001 | Yes |
| Top-10 keyword concentration | tDCS | 10,576 | 10,576 | 0.2727 | 0.1933 | 0.1683 | 0.2181 | <0.001 | <0.001 | Yes |
| Top-10 keyword concentration | Strict-core tFUS/TUS/LIFU | 843 | 850 | 0.2727 | 0.2088 | 0.1877 | 0.2306 | <0.001 | <0.001 | Yes |

Notes: Expanded comparator corpora incorporated 177 additional TMS records and seven additional tFUS/TUS/LIFU records meeting the same strict-core definition used in the primary analysis; the tDCS corpus was unchanged. The seven additional strict-core tFUS/TUS/LIFU records were identified after reclassification of 20 broader-search records retained as relevant to the broader ultrasound sensitivity-search scope; 12 of the remaining records were classified as core-adjacent and one as non-core. For each comparator, 10,000 samples of 162 records were drawn without replacement to reproduce the 2017–2026 publication-year distribution of the TI corpus. Author-keyword Shannon diversity and Top-10 keyword concentration were calculated using the same preprocessing and synonym mapping as in the primary matched-resampling analysis. Empirical P values used the prespecified directional tests applied in the primary analysis (lower for Shannon diversity and upper for Top-10 keyword concentration). P values were adjusted using the Benjamini–Hochberg procedure across the complete family of 18 matched-resampling comparisons. With 10,000 iterations and add-one correction, the minimum attainable one-sided empirical P value was 1/10,001 (approximately 0.00010). Although the tDCS corpus was unchanged, the expanded-comparator analysis was rerun as a new full simulation rather than by reusing the primary resampling draws. The resulting tDCS resampling distributions therefore differed slightly from those of the primary analysis. The Top-10 concentration summaries coincide at four decimal places because this frequency-based index takes discrete values over the keyword-occurrence denominator; the corresponding full-precision summaries nevertheless differed slightly between the two runs.

Abbreviations: TI, temporal interference stimulation; TMS, transcranial magnetic stimulation; tDCS, transcranial direct current stimulation; tFUS, transcranial focused ultrasound; TUS, transcranial ultrasound stimulation; LIFU, low-intensity focused ultrasound; BH, Benjamini–Hochberg.

## **Table S20. Two-sided empirical-P sensitivity analysis of thematic diversity and keyword concentration.**

| Analysis | Indicator | Comparison modality | TI observed | Matched median | 2.5th percentile | 97.5th percentile | Two-sided empirical P | Two-sided BH-adjusted P |
| --- | --- | --- | --- | --- | --- | --- | --- | --- |
| Primary analysis | Author-keyword Shannon diversity | TMS | 5.5137 | 5.9597 | 5.8252 | 6.0876 | <0.001 | <0.001 |
| Primary analysis | Author-keyword Shannon diversity | tDCS | 5.5137 | 5.8541 | 5.7238 | 5.9827 | <0.001 | <0.001 |
| Primary analysis | Author-keyword Shannon diversity | Strict-core tFUS/TUS/LIFU | 5.5137 | 5.7238 | 5.6084 | 5.8315 | <0.001 | 0.002 |
| Primary analysis | Top-10 keyword concentration | TMS | 0.2727 | 0.1716 | 0.1484 | 0.1968 | <0.001 | <0.001 |
| Primary analysis | Top-10 keyword concentration | tDCS | 0.2727 | 0.1933 | 0.1683 | 0.2181 | <0.001 | <0.001 |
| Primary analysis | Top-10 keyword concentration | Strict-core tFUS/TUS/LIFU | 0.2727 | 0.2092 | 0.1885 | 0.2313 | <0.001 | <0.001 |
| Expanded comparator sensitivity | Author-keyword Shannon diversity | TMS | 5.5137 | 5.9594 | 5.8295 | 6.0900 | <0.001 | <0.001 |
| Expanded comparator sensitivity | Author-keyword Shannon diversity | tDCS | 5.5137 | 5.8538 | 5.7235 | 5.9825 | <0.001 | <0.001 |
| Expanded comparator sensitivity | Author-keyword Shannon diversity | Strict-core tFUS/TUS/LIFU | 5.5137 | 5.7263 | 5.6147 | 5.8344 | <0.001 | 0.002 |
| Expanded comparator sensitivity | Top-10 keyword concentration | TMS | 0.2727 | 0.1711 | 0.1475 | 0.1954 | <0.001 | <0.001 |
| Expanded comparator sensitivity | Top-10 keyword concentration | tDCS | 0.2727 | 0.1933 | 0.1683 | 0.2181 | <0.001 | <0.001 |
| Expanded comparator sensitivity | Top-10 keyword concentration | Strict-core tFUS/TUS/LIFU | 0.2727 | 0.2088 | 0.1877 | 0.2306 | <0.001 | <0.001 |

Notes: Two-sided empirical P values were recalculated from the existing 10,000-iteration matched-resampling distributions; no additional resampling was performed. The analysis was conducted separately for the primary comparison and the expanded-comparator sensitivity analysis. For each comparison, the two-sided empirical P value was calculated as P = min[1, 2 × (min(b_lower, b_upper) + 1)/(B + 1)], where B = 10,000, b_lower was the number of resampled values less than or equal to the observed TI value, and b_upper was the number greater than or equal to the observed value. Benjamini–Hochberg correction was applied separately across the complete 18-test family within each analysis. All six thematic comparisons remained significant in both analyses after multiplicity correction; the largest BH-adjusted P value was 0.002. This analysis was performed as a robustness check and does not replace the prespecified directional empirical tests used in the primary analysis.

Abbreviations: TI, temporal interference stimulation; TMS, transcranial magnetic stimulation; tDCS, transcranial direct current stimulation; tFUS, transcranial focused ultrasound; TUS, transcranial ultrasound stimulation; LIFU, low-intensity focused ultrasound; BH, Benjamini–Hochberg.
